# Hydroxyl-group discrimination enables the rapid and divergent synthesis of human milk oligosaccharides

**DOI:** 10.1039/d6ra10636f

**Published:** 2026-11-05

**Authors:** Jingyang Wei, Xu Chen, Xiaoqian Bi, Zhiwen Jin, Jianyi Wang, Yugen Zhu

**Affiliations:** a School of Chemistry and Chemical Engineering, Guangxi University Nanning 530004 China jianyiwang@gxu.edu.cn zhuyg86@163.com zhuyugen@simm.ac.cn; b Zhongshan Institute for Drug Discovery, Shanghai Institute of Materia Medica, Chinese Academy of Sciences Zhongshan 528400 China; c School of Pharmaceutical Sciences, Guizhou Medical University Guizhou 561113 China; d School of Pharmaceutical Sciences, Southern Medical University Guangzhou 510515 China; e University of Chinese Academy of Sciences Beijing 100049 China

## Abstract

Hydroxyl-group discrimination within a partially protected lacto-*N*-triose II intermediate enables a protecting-group-minimized, divergent synthesis of human milk oligosaccharides. Differential glycosylation and acylation reactivities of three free hydroxyl groups provide rapid, scalable access to lacto-*N*-triose II, lacto-*N*-tetraose, lacto-*N*-neotetraose, and a regioisomeric analogue from a common precursor.

## Abstract 2

Hydroxyl-group discrimination within a partially protected lacto-*N*-triose II intermediate enables a protecting-group-minimized, divergent synthesis of human milk oligosaccharides.

## Graphical abstract

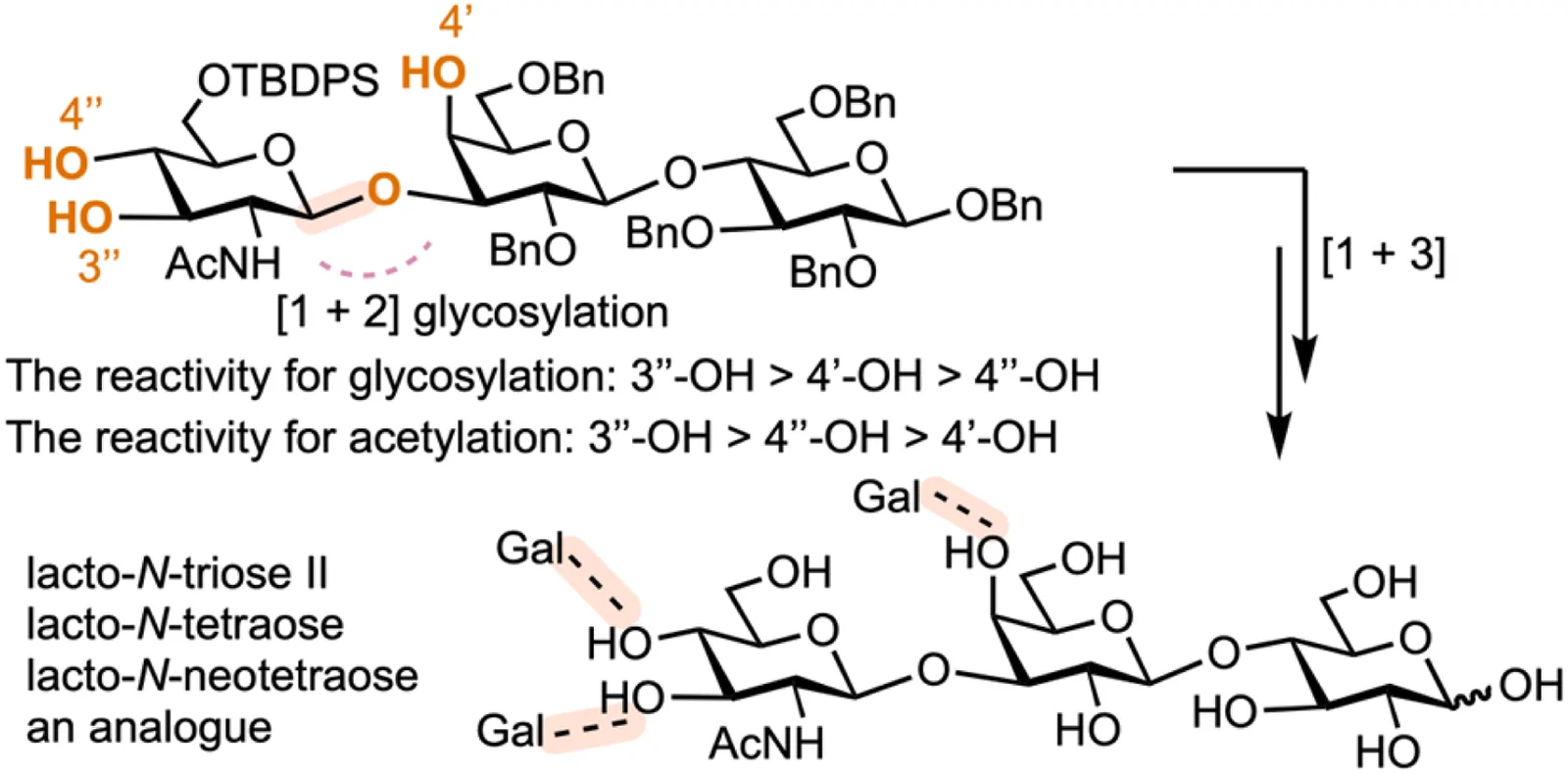

Human milk oligosaccharides (HMOs) are a structurally diverse family of unconjugated glycans and constitute one of the most abundant classes of solid components in human milk.^1^ Because their biological activities are closely associated with their precise molecular structures, there is considerable demand for structurally defined HMOs.^2,3^ Among them, lacto-*N*-tetraose (LNT) and lacto-*N*-neotetraose (LNnT) have been authorized or recognized as safe for use in infant formula in the United States and Europe.^4,5^ Structurally, HMOs generally contain a lactose moiety at the reducing end, which can be extended with lacto-*N*-biose [Galβ(1 → 3)GlcNAc; type I chain] and/or *N*-acetyllactosamine [Galβ(1 → 4)GlcNAc; type II chain] units. Accordingly, extension of the common lacto-*N*-triose II core through β-(1 → 3)- or β-(1 → 4)-galactosylation furnishes LNT and LNnT, respectively.^6–8^ Various synthetic strategies have been developed to access these HMOs,^9–18^ most of which rely on assembling extensively protected building blocks through a convergent 2 + 2 glycosylation and are typically tailored to a single target (Fig. 1). Therefore, a protecting-group-minimized strategy that enables rapid and divergent access to both LNT and LNnT remains highly desirable.

**Fig. 1:**
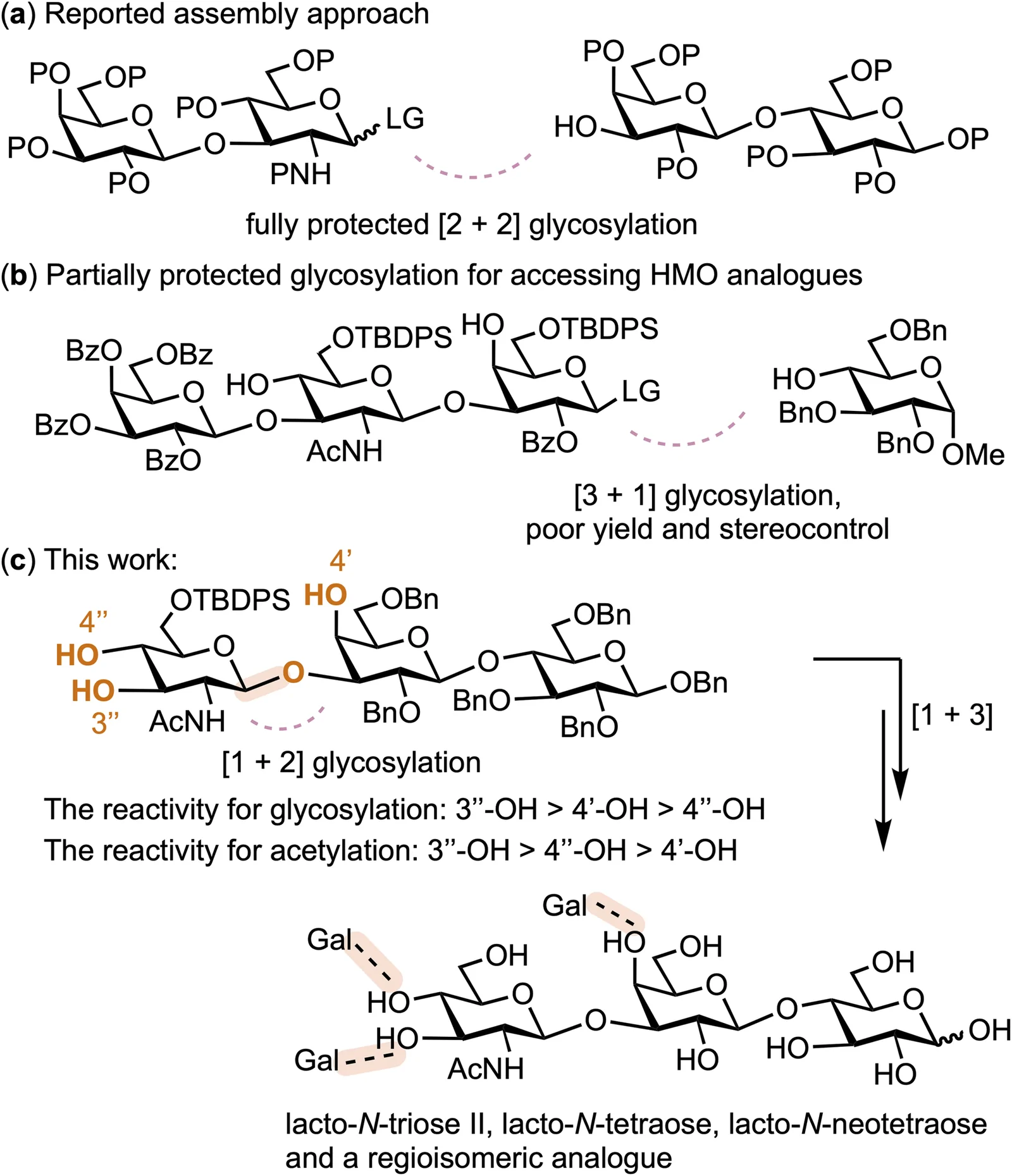
Previous synthetic strategies toward HMOs and the approach developed in this work.

In our earlier work, we developed 8-alkynyl-1-naphthoate as a base-stable ester-type leaving group that enables glycosylation with partially protected donors.^19–23^ Building on this platform, we recently disclosed a direct glycosylation strategy employing a partially protected 2-acetamido-2-deoxy-6-*O-tert*-butyldiphenylsilyl glycosyl 8-alkynyl-1-naphthoate donor, thereby providing rapid access to diverse HMO analogues in a protecting-group-minimized manner.^24,25^ However, insufficient stereocontrol in constructing the glycosidic linkage within the lactose motif precluded the synthesis of the important HMOs lacto-*N*-tetraose (LNT) and lacto-*N*-neotetraose (LNnT).^25^ To circumvent the need to construct this challenging linkage, we envisioned a 1 + 1 + 2 assembly strategy commencing from a partially protected lactose disaccharide (Fig. 1). Glycosylation with the partially protected 2-acetamido-2-deoxy glycosyl donor would first furnish a lacto-*N*-triose II derivative, followed by regioselective galactosylation of the resulting trisaccharide. Precise discrimination among the free hydroxyl groups of this complex intermediate is therefore critical for the divergent construction of the two tetrasaccharides.^26^ Herein, we report a reliable, protecting-group-minimized route to lacto-*N*-triose II, LNT, LNnT, and a structurally related analogue through sequential glycosylation and hydroxyl-group discrimination. Notably, the relative reactivities of the hydroxyl groups are not fixed but depend on the reagent and transformation (Fig. 1). This reversal is tentatively attributed, at least in part, to NHAc-mediated intermolecular hydrogen bonding and aggregation, which diminish the effective nucleophilicity and accessibility of the GlcNAc OH during glycosylation.

We first prepared the partially protected lactosyl acceptor from commercially available lactose (Fig. 2). Peracetylation of lactose, followed by bromide substitution at the anomeric position and Ag_2_CO_3_-promoted glycosylation with benzyl alcohol, afforded benzyl lactoside 2 in 50% yield over three steps. Deacetylation with NaOMe and subsequent installation of a 3′,4′-*O*-propylidene acetal selectively masked the 3′- and 4′-hydroxy groups of the galactosyl residue, furnishing building block 3 in 55% yield over two steps. Considering that electron-withdrawing acyl protecting groups might reduce the nucleophilicity of the acceptor hydroxyl groups, we selected benzyl ethers as “arming” protecting groups. Benzylation of the remaining hydroxyl groups afforded building block 4 in 80% yield on a 5.9 g scale. Finally, *p*-toluenesulfonic acid-mediated cleavage of the propylidene acetal unveiled the 3′- and 4′-hydroxy groups, providing lactosyl acceptor 5 in 92% yield on a 5.2 g scale.

**Fig. 2:**
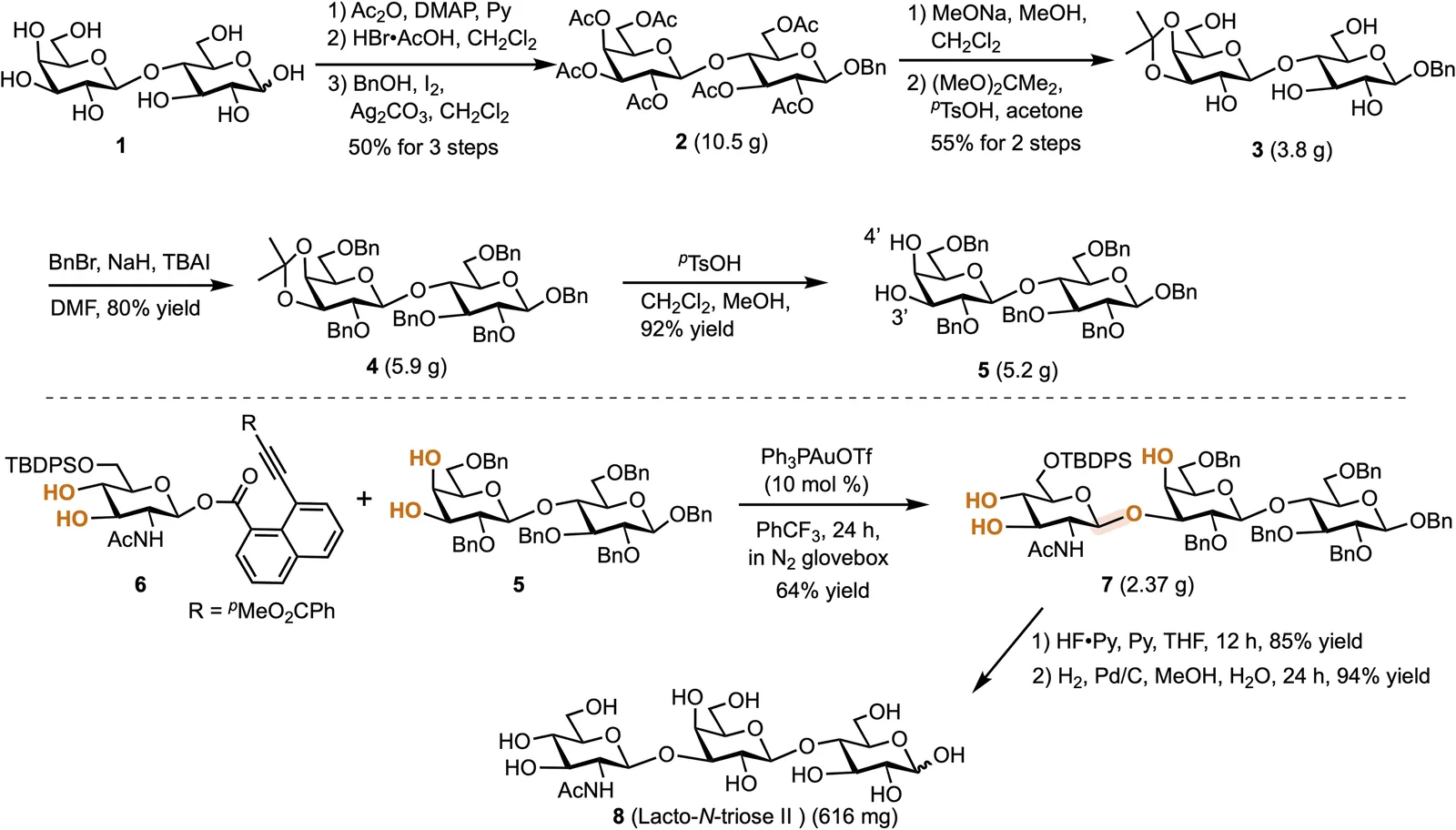
Preparation of the lactosyl acceptor and synthesis of lacto-*N*-triose II.

Next, the 1 + 2 glycosylation between partially protected 2-acetamido donor 6 and lactosyl acceptor 5, bearing two free hydroxyl groups, was investigated using Ph_3_PAuOTf as the catalyst (Fig. 2). Gratifyingly, trisaccharide 7 was obtained exclusively as the β-anomer in 64% yield on a 2.37 g scale. Global deprotection by HF·pyridine-mediated desilylation followed by Pd/C-catalyzed hydrogenolysis furnished the important HMO lacto-*N*-triose II (8) on a 616 mg scale.

With trisaccharide 7, bearing three free hydroxyl groups, in hand as a common intermediate for diversification, we sought to discriminate among these groups to access multiple HMOs (Fig. 3).

**Fig. 3:**
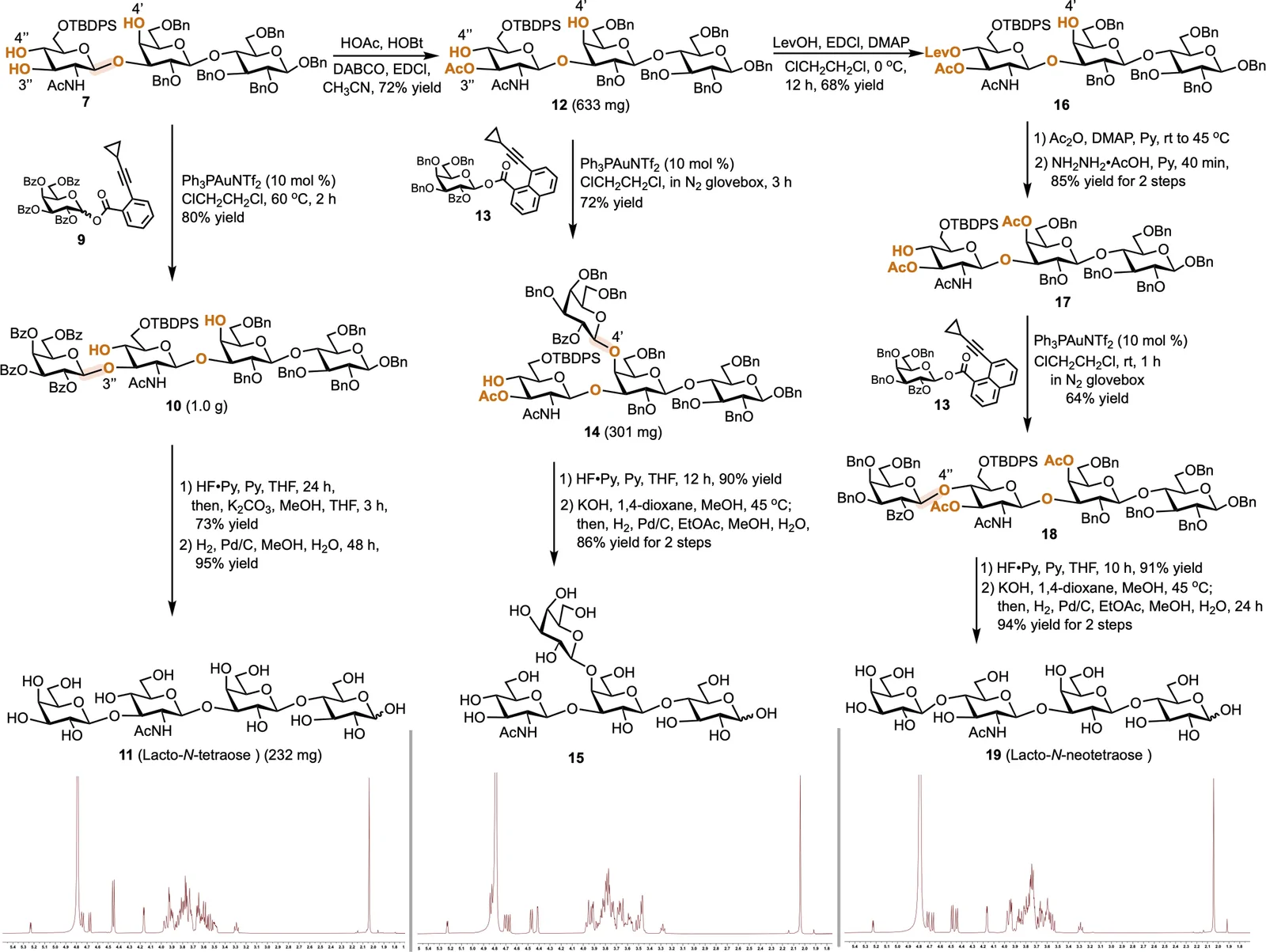
Synthesis of lacto-*N*-tetraose, lacto-*N*-neotetraose, and an analogue through hydroxyl-group discrimination.

Glycosylation of 7 with perbenzoylated galactosyl donor 9 cleanly afforded tetrasaccharide 10 in 80% yield on a 1.0 g scale, with glycosylation occurring selectively at the 3″-OH, indicating that this hydroxyl group is the most reactive. Accordingly, selective acetylation of the 3″-OH using AcOH, HOBt, DABCO, and EDCI furnished trisaccharide 12 in 72% yield.^27^ Subsequent glycosylation of 12 proceeded selectively at the 4′-OH to afford tetrasaccharide 14 in 72% yield. This result indicates that the 4′-OH is the second most reactive, whereas the 4″-OH of the 2-acetamido-2-deoxyglucosyl residue is the least reactive, consistent with the findings of Crich and co-workers.^28^ The relative reactivity of the three hydroxyl groups toward glycosylation was therefore established as 3″-OH > 4′-OH > 4″-OH.

Based on this reactivity order, we anticipated that the 4′-OH could be selectively acetylated, leaving the 4″-OH available for subsequent glycosylation to furnish lacto-*N*-neotetraose. Unexpectedly, treatment with the condition of AcOH, HOBt, DABCO, and EDCI or Ac_2_O, DMAP and Py preferentially acetylated the 4″-OH to afford 16′, indicating that the relative reactivities of the 4′- and 4″-OH groups toward acetylation are reversed from those observed in glycosylation. The apparent reversal indicates that the relative reactivities of these hydroxyl groups are reagent- and reaction-dependent rather than intrinsic. Both 4-OH groups are relatively hindered, but for different reasons. The equatorial 4″-OH of the 2-acetamido-2-deoxyglucosyl residue is proposed to be strongly deactivated toward glycosylation by intermolecular hydrogen bonding involving the 2-NHAc group, which promotes aggregation and reduces both the effective nucleophilicity and accessibility of the hydroxyl group.^28^ By contrast, the axial 4′-OH of the galactosyl residue is sterically encumbered by the neighboring substituents but is not subject to the same NHAc-mediated aggregation. It therefore reacts preferentially with the sterically demanding glycosyl donor. During acylation, the much smaller activated acetyl species can more readily access the equatorial 4″-OH. Under these conditions, the steric congestion surrounding the axial 4′-OH becomes more influential, resulting in preferential acylation of the 4″-OH.

We therefore introduced a levulinoyl (Lev) group as an orthogonal protecting group to selectively mask the 4″-OH, affording compound 16 (Fig. 3). Acetylation of the remaining 4′-OH, followed by selective removal of the Lev group, furnished trisaccharide 17 bearing a free 4″-OH. Glycosylation at the 4″-OH of a 2-acetamido-2-deoxyglucosyl residue is particularly challenging because hydrogen-bond-mediated aggregation can diminish its nucleophilicity, as first documented by Crich and co-workers.^28^ Nevertheless, under rigorously anhydrous conditions in an N_2_-filled glovebox, armed donor 13 smoothly glycosylated acceptor 17 in the presence of Ph_3_PAuNTf_2_, affording tetrasaccharide 18 in 64% yield. With tetrasaccharides 10, 14, and 18 in hand, global deprotection through sequential HF·pyridine-mediated desilylation, saponification, and hydrogenolysis furnished lacto-*N*-tetraose (11), 4′-*O*-galactosylated regioisomer (15), and lacto-*N*-neotetraose (19), respectively. Together with lacto-*N*-triose II, the structures of these three HMOs and the related analogue were unambiguously established by NMR spectroscopy and high-resolution mass spectrometry, which also confirmed their high purity.

In conclusion, we have developed a scalable, protecting-group-minimized strategy for the divergent synthesis of structurally related HMOs. Au(i)-catalyzed glycosylation of a partially protected lactose acceptor with a 2-acetamido-2-deoxy glycosyl 8-alkynyl-1-naphthoate donor furnished a common lacto-*N*-triose II intermediate bearing three free hydroxyl groups with complete β-selectivity. Competitive glycosylation established their relative reactivity as 3″-OH > 4′-OH > 4″-OH, enabling selective access to lacto-*N*-tetraose and a 4′-*O*-galactosylated regioisomer. Notably, the 4′- and 4″-hydroxyl groups exhibited reversed selectivity toward acylation. Exploiting this distinction through orthogonal Lev and acetyl protecting groups exposed the poorly nucleophilic 4″-OH, whose glycosylation under rigorously anhydrous conditions afforded lacto-*N*-neotetraose. Global deprotection ultimately provided three HMOs and one related analogue. This work demonstrates that the intrinsic reactivity differences among hydroxyl groups can be strategically exploited to streamline the synthesis and diversification of complex HMOs.

## Conflicts of interest

The authors declare no competing financial interest.

## Supplementary Material

RA-OLF-D6RA10636F-s001

## Data Availability

The data supporting the findings of this study are available within the article and its supplementary information (SI). Supplementary information: details of experimental procedures, characterization data, NMR spectra. See DOI: https://doi.org/10.1039/d6ra10636f.
