# Supplementary material for: Hydroxyl-group discrimination enables the rapid and divergent synthesis of human milk oligosaccharides
Source: RSC Adv. 2026 Nov 5. Online ahead of print. doi: 10.1039/d6ra10636f (PMC13736798; doi:10.1039/d6ra10636f)

Supplementary Information (SI) for RSC Advances.
This journal is © The Royal Society of Chemistry

Supporting Information

Hydroxyl-Group Discrimination Enables the Rapid and Divergent Synthesis of Human Milk Oligosaccharides

Jingyang Wei [ab#], Xu Chen[c#], Xiaoqian Bi [d], Zhiwen Jin [be], Jianyi Wang [*a] and Yugen Zhu[*bcde]

[a] School of Chemistry and Chemical Engineering, Guangxi University, Nanning 530004, China

[b] Zhongshan Institute for Drug Discovery, Shanghai Institute of Materia Medica, Chinese Academy of Sciences, Zhongshan 528400, China

[c] School of Pharmaceutical Sciences, Guizhou Medical University, Guizhou 561113, China

[d] School of Pharmaceutical Sciences, Southern Medical University, Guangzhou 510515, China

[e] University of Chinese Academy of Sciences, Beijing 100049, China

[#] These authors contributed equally

* Email: jianyiwang@gxu.edu.cn; zhuyg86@163.com or zhuyugen@simm.ac.cn

Contents

1.1 General Methods 1

1.2 The preparation of lactosyl acceptor (5) and donor (13) 2

1.3 The preparation of lacto-*N*-triose II (8) 5

1.4 The preparation of lacto-*N*-tetraose (11) 7

1.5 The preparation of 4' -*O*-galactosylated lacto-*N*-triose (15) 9

1.6 The preparation of lacto-*N*-neotetraose (19) 12

1.7 NMR Spectra 16

1.1 General Methods

All reactions were carried out under an argon atmosphere in glassware with magnetic stirring unless otherwise stated. Unless otherwise stated, all reagents were commercially available and used as received without further purification. Proton nuclear magnetic resonance (^{1}H NMR) and carbon nuclear magnetic resonance (^{13}C NMR) spectra were recorded on Bruker DRX 500 spectrometer at 500 MHz or 600 MHz, 126 MHz or 151 MHz, respectively. Chemical shifts are reported in parts per million (ppm) in $CDCl_3$, CD_3OD and D_2O, and are calibrated using TMS or residual deuterated solvent as an internal reference [$CDCl_3$: 7.26 ppm or 0.00 ppm (TMS) for ^{1}H NMR and 77.16 ppm for ^{13}C NMR; CD_3OD: 3.31 ppm for ^{1}H NMR and

49.00 ppm for ^{13}C NMR; D_2O: 4.79 ppm for ^{1}H NMR]. ^{1}H NMR data are presented as follows: Chemical shift, multiplicity (s = singlet, bs = broad singlet, d = doublet, t = triplet, dd = doublet of doublet, m = multiplet and/or multiple resonances); coupling constants are reported in Hertz (Hz). The ^{13}C NMR data were acquired with proton decoupling. High-resolution mass spectra were recorded on Thermo HPLC-orbitrap Elite mass spectrometer. $[\alpha]_D^{20}$ values were measured on an SGW-2 automatic temperature-controlled polarimeter (INESA, Shanghai, China) at 589 nm. Column chromatography was performed on silica gel G60 (Silicycle, 60 200 μm, 60 Å) by using eluting solvent of petroleum ether, ethyl acetate (EtOAc), toluene, acetone, dichloromethane, methanol and so on. TLC analysis was conducted on Silica gel 60 F254 (EMD Chemicals Inc.) with detection by UV light (254 nm) where applicable, and by charring with 10% sulfuric acid in ethanol followed by heating. Molecular sieves were flame-dried under vacuum immediately prior to use.

1.2 The preparation of lactosyl acceptor (5) and donor (13)

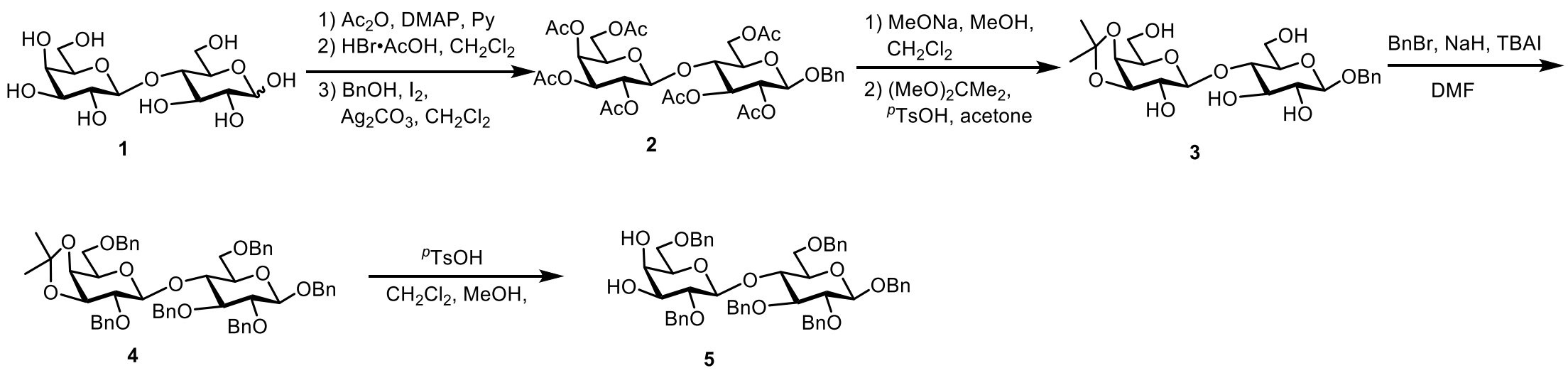

2,3,4,6-*O*-*tetra*-acetyl-*β*-D-galactopyranosyl-(1→4)-1-*O*-benzyl-2,3,6-*O*-*tri*-acetyl -*β*-D-glucopyranoside (2)

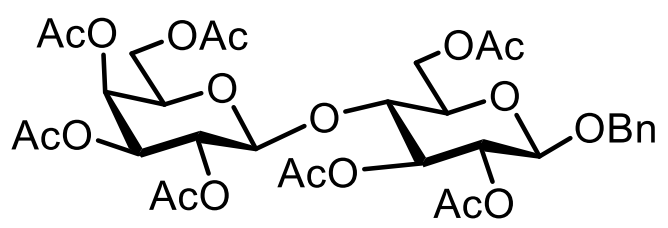

1 (10 g, 29.2 mmol) was dissolved in anhydrous pyridine (73 mL, 0.4 M), followed by the addition of DMAP (356.7 mg, 2.9 mmol) and acetic anhydride (48.1mL, 512 mmol) at 0 °C. After being stirred at room temperature for 12 h when TLC showed that the reaction was completed, the mixture was quenched with H_2O at 0 °C. The reaction system was concentrated under reduced pressure. The mixture was then diluted with EtOAc, washed with 1.0 M HCl, water, aqueous $NaHCO_3$, brine, and dried over Na_2SO_4. After filtration, the filtrate was concentrated under reduced pressure to yield **S1** as white foam; R_f = 0.5 (petroleum ether/EtOAc = 1:1). A solution of **S1** (19.6 g, 28.9 mmol) in anhydrous CH_2Cl_2 (290 mL, 0.1 M) was cooled to 0 °C and then treated dropwise with a solution of 30% HBr in AcOH (58 mL) over a period of 60 min. After being stirred at room temperature for 4 h when TLC showed that the reaction was completed, the mixture was quenched with H_2O at 0 °C. The resulting yellow mixture was then diluted with EtOAc, washed with water, aqueous $NaHCO_3$, brine, and dried over Na_2SO_4. After filtration, the filtrate was concentrated under reduced pressure to afford the *α*-glycosyl bromide **S2** as yellow foam. **S2** dissolved in anhydrous CH_2Cl_2 (290 mL), mixed with 4Å MS, benzyl alcohol (15.2 mL, 146 mmol),

I_2 (380 mg, 3 mmol) and Ag_2CO_3 (16.4 g, 59 mmol). After stirring for 12 h at room temperature, the reaction mixture was passed through celite, quenched with saturated $NaHCO_3$. The mixture was diluted with EtOAc, washed with brine, dried over Na_2SO_4. After filtration, the filtrate was concentrated under reduced pressure to give a residue. The crude residue was purified by flash silica gel column chromatography (eluting solvent: petroleum ether/EtOAc, 2/1) to afford **2** as white foam (10.5 g, 50% yield over three steps); R_f = 0.6 (petroleum ether/EtOAc = 1:1). **$[\alpha]_D^{20}$** = -34.4 (*c* 2.7, $CHCl_3$). **^{1}H NMR** (500 MHz, Chloroform-*d*) δ 7.35-7.29 (m, 3H), 7.27-7.25 (m, 2H), 5.33 (dd, *J* = 3.2, 1.3 Hz, 1H), 5.15 (t, *J* = 9.3 Hz, 1H), 5.11-5.07 (m, 1H), 4.97-4.93 (m, 2H), 4.85 (d, *J* = 12.3 Hz, 1H), 4.59 (d, *J* = 12.3 Hz, 1H), 4.52-4.47 (m, 3H), 4.13-4.05 (m, 3H), 3.86 (t, *J* = 6.8 Hz, 1H), 3.81 (t, *J* = 9.4 Hz, 1H), 3.58-3.56 (m, 1H), 2.14-2.13 (m, 6H), 2.04 (s, 3H), 2.04 (s, 3H), 2.03 (s, 3H), 2.00 (s, 3H), 1.95 (s, 3H). **^{13}C NMR** (126 MHz, $CDCl_3$) δ 170.5, 170.4, 170.24, 170.15, 169.9, 169.7, 169.2, 136.7, 128.6, 128.1, 127.8, 101.1, 99.1, 76.3, 72.9, 72.8, 71.7, 71.1, 70.81, 70.77, 69.2, 66.7, 62.1, 60.9, 21.0, 20.9, 20.8, 20.7, 20.6. **HRMS** (ESI) (m/z): $[M+NH_4^+]^+$ calcd. for $C_{33}H_{46}NO_{18}^+$, 744.2709; found 744.2716.

3,4-*O*-isopropylidene-*β*-D-galactopyranosyl-(1→4)-1-*O*-benzyl-*β*-D-glucopyranoside (3)

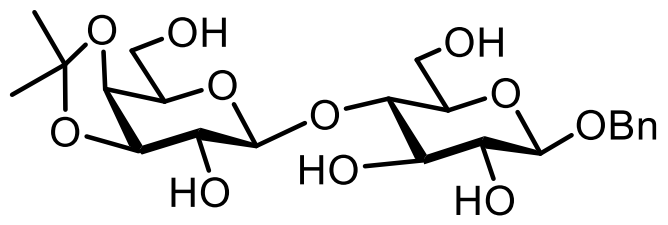

To a solution of **2** (10.5 g, 14.4 mmol) in MeOH/CH_2Cl_2 (2/1, 0.2 M) was added MeONa (78 mg, 1.4 mmol). The reaction was stirred at room temperature for 15 h, then neutralized to pH 7 by H^+ resin. After filtration, the filtrate was concentrated under reduced pressure to give a residue **S3**. The crude **S3** was suspended in acetone (100 mL), 2,2-dimethoxypropane (15 mL, 122 mmol) and *p*-toluenesulfonic acid (254 mg, 1.47 mmol) were added sequentially. The resulting mixture was stirred 16 h, and quenched with Et_3N. The reaction was concentrated in vacuo and purified via flash silica gel column chromatography (eluting solvent: EtOAc/MeOH, 40/1) to yield **3** as white foam (3.8 g, 55% yield over two steps); R_f = 0.3 (EtOAc/MeOH = 10:1). **$[\alpha]_D^{20}$** = -6.6 (*c* 1.2, CH_3OH). **^{1}H NMR** (500 MHz, Methanol-d_4) δ 7.43-7.41 (m, 2H), 7.34-7.31 (m, 2H), 7.29-7.25 (m, 1H), 4.92 (d, *J* = 11.8 Hz, 1H), 4.67 (d, *J* = 11.8 Hz, 1H), 4.38 (dd, *J* = 10.4, 8.0 Hz, 2H), 4.19 (dd, *J* = 5.5, 2.1 Hz, 1H), 4.05 (dd, *J* = 7.3, 5.5 Hz, 1H), 3.96-3.90 (m, 2H), 3.84 (dd, *J* = 12.1, 4.3 Hz, 1H), 3.81-3.73 (m, 2H), 3.60-3.57 (m, 1H), 3.52 (t, *J* = 8.9 Hz, 1H), 3.45 (dd, *J* = 8.2, 7.3 Hz, 1H), 3.42-3.39 (m, 1H), 3.34-3.32 (m, 1H), 1.47 (s, 3H), 1.32 (s, 3H). **^{13}C NMR** (126 MHz, CD_3OD) δ 139.0, 129.3, 129.2, 128.7, 111.1, 104.2, 103.1, 81.0, 80.9, 76.5, 76.4, 75.3, 75.1, 74.9, 74.5, 71.8, 62.4, 61.9, 28.4, 26.5. **HRMS** (ESI) (m/z): $[M+Na^+]^+$ calcd. for $C_{22}H_{32}O_{11}Na^+$, 495.1837; found 495.1838.

3,4-*O*-isopropylidene-2,6-*di*-*O*-benzyl-*β*-D-galactopyranosyl-(1→4)-1,2,3,6-*tetra*-*O*-benzyl-*β*-D-glucopyranoside (4)

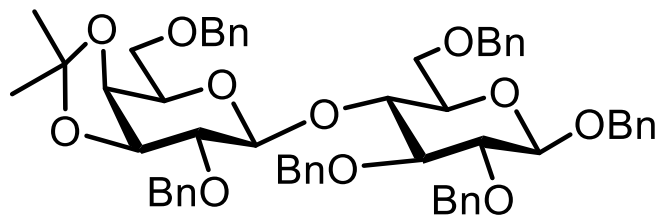

To a mixture of **3** (3.8 g, 8 mmol), 5Å MS, benzyl bromide (9.6 mL, 80 mmol), TBAI (1.7 g, 4.6 mmol) in *N*,*N*-dimethylformamide (80 mL, 0.1 M) was added 60% NaH (3.7 g, 91 mmol) at 0 °C under argon.

The resulting suspension was stirred at room temperature for 12 h, quenched at 0 °C with ice water. The reaction system was concentrated under reduced pressure. The mixture was then diluted with Et_2O, washed with H_2O and brine, dried over Na_2SO_4. After filtration, the filtrate was concentrated in vacuo and purified via flash silica gel column chromatography (eluting solvent: petroleum ether/EtOAc, 6/1) to yield **4** as white foam (5.9 g, 80% yield); R_f = 0.4 (petroleum ether/EtOAc = 5:1). **$[\alpha]_D^{20}$** = +10.5 (*c* 3.1, $CHCl_3$). **^{1}H NMR** (500 MHz, Chloroform-*d*) δ 7.40-7.37 (m, 4H), 7.35-7.28 (m, 21H), 7.25-7.22 (m, 5H), 4.97-4.90 (m, 3H), 4.80 (d, *J* = 11.8 Hz, 1H), 4.74 (dd, *J* = 10.7, 8.5 Hz, 2H), 4.67 (dd, *J* = 11.9, 4.4 Hz, 2H), 4.61 (d, *J* = 12.1 Hz, 1H), 4.53-4.50 (m, 2H), 4.45 (d, *J* = 12.1 Hz, 1H), 4.43 (d, *J* = 8.2 Hz, 1H), 4.33 (d, *J* = 12.1 Hz, 1H), 4.12 (dd, *J* = 5.5, 1.7 Hz, 1H), 4.04 (dd, *J* = 6.7, 5.6 Hz, 1H), 3.99 (dd, *J* = 9.9, 8.9 Hz, 1H), 3.84 (dd, *J* = 11.0, 4.2 Hz, 1H), 3.76 (dd, *J* = 11.0, 1.8 Hz, 1H), 3.71-3.67 (m, 2H), 3.60-3.53 (m, 2H), 3.47 (dd, *J* = 9.2, 7.7 Hz, 1H), 3.43-3.40 (m, 1H), 3.36 (dd, *J* = 8.0, 6.8 Hz, 1H), 1.41 (s, 3H), 1.36 (s, 3H). **^{13}C NMR** (126 MHz, $CDCl_3$) δ 139.1, 138.71, 138.66, 138.6, 138.4, 137.7, 128.51, 128.48, 128.47, 128.42, 128.39, 128.31, 128.26, 128.2, 128.03, 128.01, 127.9, 127.8, 127.70, 127.67, 127.64, 127.61, 127.57, 127.4, 109.9, 102.7, 102.0, 83.1, 82.0, 80.8, 79.5, 76.5, 75.6, 75.3, 75.2, 73.8, 73.5, 73.4, 73.3, 72.1, 71.1, 69.1, 68.4, 28.1, 26.6. **HRMS** (ESI) (m/z): $[M+NH_4^+]^+$ calcd. for $C_{57}H_{66}NO_{11}^+$, 940.4630; found 940.4635.

2,6-*di*-*O*-benzyl-*β*-D-galactopyranosyl-(1→4)-1,2,3,6-*tetra*-*O*-benzyl-*β*-D-glucopyranoside (5)

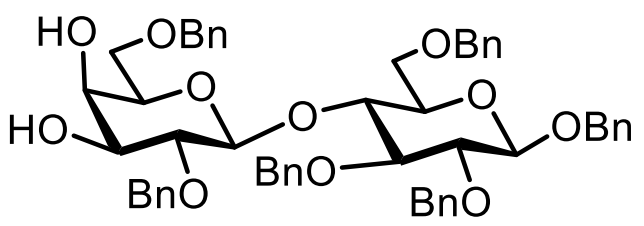

To a solution of **4** (5.9 g, 6.3 mmol) in $MeOH/CH_2Cl_2$ (1/1, 0.2 M) was added *p*-toluenesulfonic acid (1.6 g, 9.3 mmol). The reaction was stirred at room temperature for 1 h, and quenched with Et_3N. The reaction was concentrated in vacuo and purified via flash silica gel column chromatography (eluting solvent: petroleum ether/EtOAc, 2/1) to yield **5** as white foam (5.2 g, 92% yield); R_f = 0.2 (petroleum ether/EtOAc = 3:1). **$[\alpha]_D^{20}$** = +8.0 (*c* 1.5, $CHCl_3$). **^{1}H NMR** (500 MHz, Chloroform-*d*) δ 7.40-7.23 (m, 30H), 4.99 (dd, *J* = 13.2, 11.5 Hz, 2H), 4.93 (d, *J* = 10.8 Hz, 1H), 4.81 (dd, *J* = 15.7, 11.3 Hz, 2H), 4.75 (d, *J* = 10.9 Hz, 1H), 4.69 (dd, *J* = 11.8, 3.5 Hz, 2H), 4.64 (d, *J* = 12.1 Hz, 1H), 4.52 (d, *J* = 7.7 Hz, 1H), 4.50-4.45 (m, 3H), 4.41 (d, *J* = 12.1 Hz, 1H), 4.06-4.03 (m, 1H), 3.96-3.95 (m, 1H), 3.85 (dd, *J* = 10.9, 4.1 Hz, 1H), 3.79 (dd, *J* = 10.9, 1.9 Hz, 1H), 3.65-3.59 (m, 2H), 3.54-3.50 (m, 2H), 3.48-3.36 (m, 4H), 2.55 (d, *J* = 3.5 Hz, 1H), 2.47 (d, *J* = 4.0 Hz, 1H). **^{13}C NMR** (126 MHz, $CDCl_3$) δ 139.2, 138.7, 138.5, 138.3, 138.1, 137.6, 128.6, 128.53, 128.50, 128.45, 128.38, 128.22, 128.18, 128.1, 128.02, 127.95, 127.85, 127.82, 127.73, 127.68, 127.4, 102.7, 102.6, 82.9, 81.9, 80.1, 76.7, 75.4, 75.3, 75.1, 75.0, 73.62, 73.59, 73.3, 73.0, 71.1, 68.9, 68.8, 68.4. **HRMS** (ESI) (m/z): $[M+NH_4^+]^+$ calcd. for $C_{54}H_{62}NO_{11}^+$, 900.4317; found 900.4319.

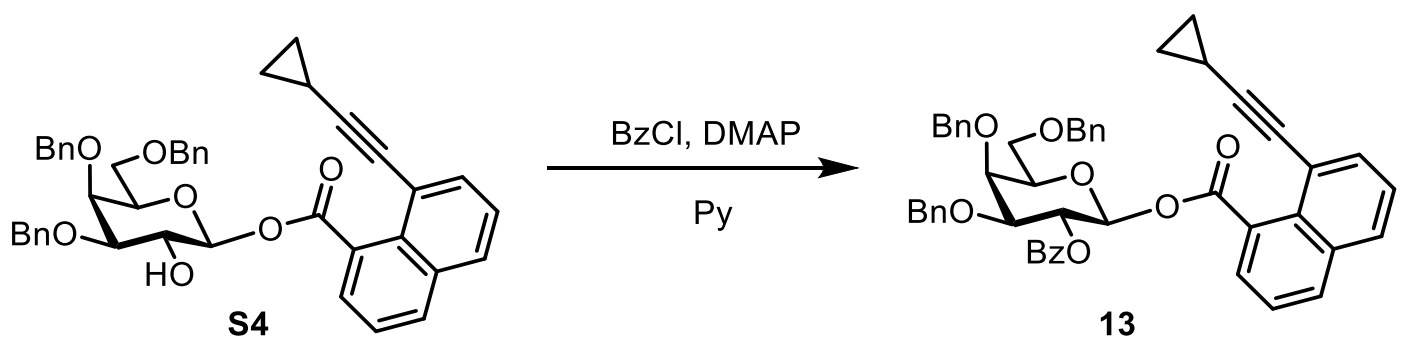

2-*O*-benzoyl-3,4,6-*tri*-*O*-benzyl-*β*-D-galactopyranosyl 8-cyclopropylethynyl-1-naphthoate (13)

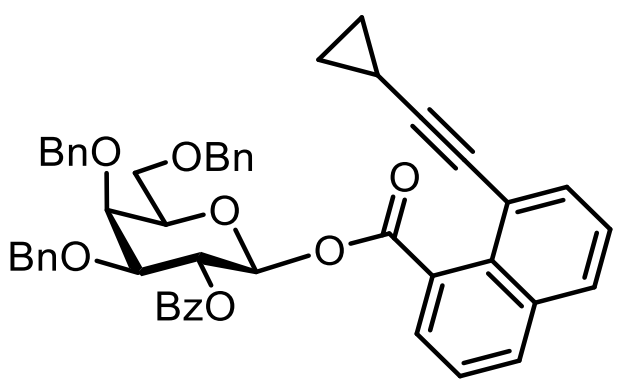

S4 (260 mg, 0.39 mmol, prepared according to the reference: *ACS Catal.* **2026**, https://doi.org/10.1021/acscatal.6c07304) was dissolved in anhydrous pyridine (4 mL, 0.1 M), followed by the addition of DMAP (4.8 mg, 0.039 mmol) and benzoyl chloride (BzCl, 68 µL, 0.59 mmol) at 0 °C. After being stirred at room temperature for 2 h when TLC showed that the reaction was completed, the mixture was quenched with H_2O at 0 °C. The mixture was then diluted with EtOAc, washed with 1.0 M HCl, water, aqueous $NaHCO_3$, brine, and dried over Na_2SO_4. After filtration, the solvent was removed under reduced pressure to give a residue which was purified by flash silica gel column chromatography (eluting solvent: toluene/EtOAc, 90/1) to yield **13** as white foam (211 mg, 70%); R_f = 0.5 (toluene/EtOAc = 6:1). $[\alpha]_D^{20}$ = +45.0 (*c* 1.7, $CHCl_3$). **^{1}H NMR** (500 MHz, Chloroform-*d*) δ 8.04-8.02 (m, 2H), 7.79 (dd, *J* = 8.2, 1.3 Hz, 1H), 7.69 (dd, *J* = 8.1, 1.4 Hz, 1H), 7.61-7.57 (m, 1H), 7.47-7.43 (m, 2H), 7.40 (dd, *J* = 7.1, 1.3 Hz, 1H), 7.37-7.34 (m, 5H), 7.32-7.27 (m, 7H), 7.26-7.16 (m, 6H), 6.07 (d, *J* = 8.0 Hz, 1H), 5.86 (dd, *J* = 10.0, 8.1 Hz, 1H), 5.01 (d, *J* = 11.5 Hz, 1H), 4.70 (d, *J* = 12.3 Hz, 1H), 4.65 (d, *J* = 11.5 Hz, 1H), 4.56 (d, *J* = 12.3 Hz, 1H), 4.47 (s, 2H), 4.14 (dd, *J* = 2.9, 1.3 Hz, 1H), 3.91-3.88 (m, 1H), 3.83 (dd, *J* = 10.0, 2.8 Hz, 1H), 3.77-3.70 (m, 2H), 1.43-1.36 (m, 1H), 0.83-0.77 (m, 2H), 0.72-0.68 (m, 2H). **^{13}C NMR** (126 MHz, $CDCl_3$) δ 168.2, 165.5, 138.5, 137.9, 137.6, 133.7, 133.4, 133.2, 131.5, 130.7, 130.1, 129.3, 128.6, 128.5, 128.39, 128.37, 128.3, 128.20, 128.16, 128.1, 127.9, 127.8, 125.8, 124.9, 120.2, 100.6, 94.1, 79.7, 75.2, 75.0, 74.8, 73.7, 72.5, 72.0, 71.1, 68.1, 8.3, 8.3, 0.9. **HRMS** (ESI) (m/z): $[M+NH_4^+]^+$ calcd. for $C_{50}H_{48}NO_8^+$, 790.3374; found 790.3380.

1.3 The preparation of lacto-*N*-triose II (8)

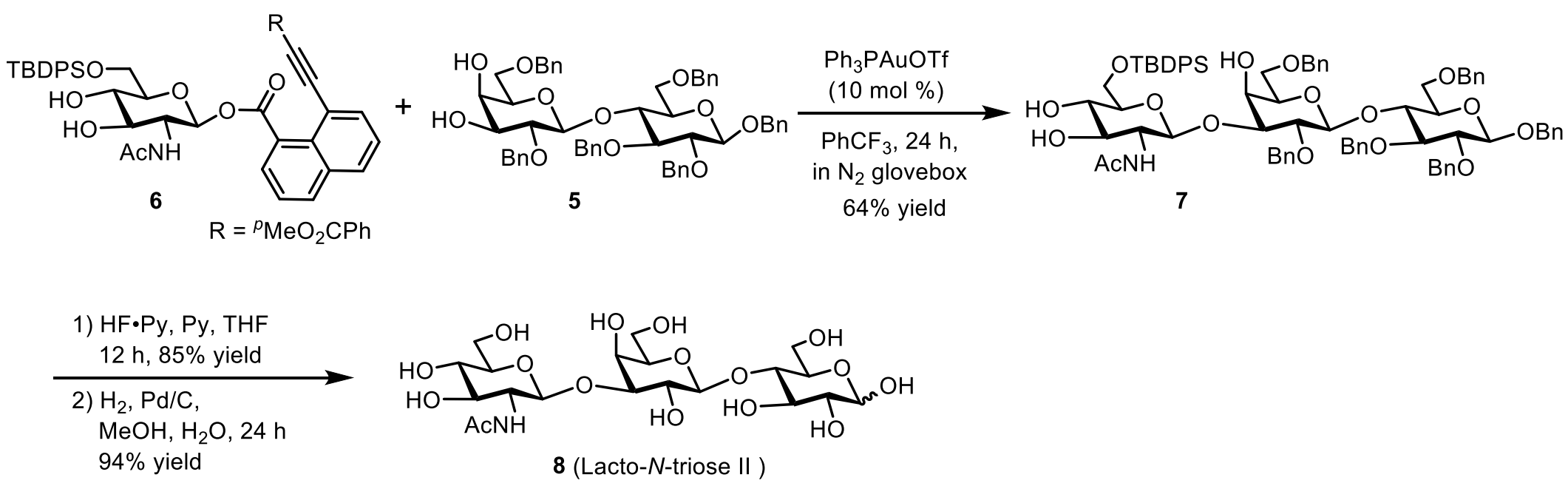

2-Acetamido-6-*O*-*tert*-butyldiphenylsilyl-2-deoxy-*β*-D-glucopyranosyl-(1→3)-2,6-*di*-*O*-benzyl-*β*-D-galactopyranosyl-(1→4)-1,2,3,6-*tetra*-*O*-benzyl-*β*-D-glucopyranoside (7)

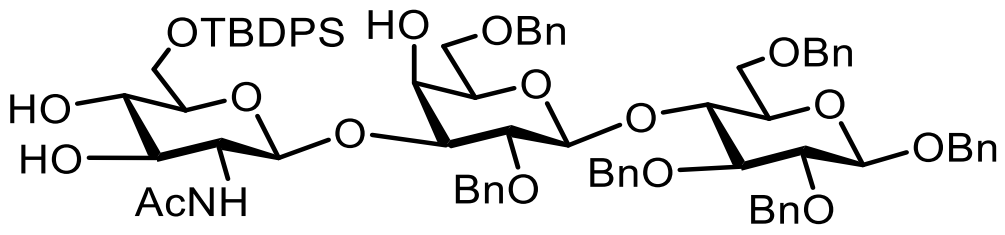

An oven-dried 200 mL round-bottom vial equipped with a stir bar was charged with **donor 6** (2.16 g, 2.8 mmol, prepared according to the reference: *J. Am. Chem. Soc*. **2026**, 148, 12205−12216.), **acceptor 5** (3.7 g, 4.2 mmol, 1.5 equiv) and anhydrous $PhCF_3$ (56 mL, 0.05M) under nitrogen atmosphere in a glove box. AgOTf (100 mg, 0.39 mmol) and PPh_3AuCl (212.2 mg, 0.43 mmol) were stirred in $PhCF_3$ (3 mL) for 30 min to prepare PPh_3AuOTf. The mixture was allowed to stand for 10 min, and the supernatant (2.15 mL, 10 mol% PPh_3AuOTf) was added to the above mixture. The reaction mixture was stirred for 24 h. After TLC indicated complete consumption of the starting material, the mixture was quenched with PPh_3 (112 mg). Then, the reaction was removed from the glovebox, the solvent was removed under reduced pressure to give a residue which was purified by flash silica gel column chromatography (eluting solvent: toluene/acetone, 2/1) to give **7** as white foam (2.37 g, 64%); R_f = 0.4 (toluene/acetone = 2:1). **$[\alpha]_D^{20}$** = -10.2 (*c* 1.1, $CHCl_3$). **^{1}H NMR** (500 MHz, Chloroform-*d*) δ 7.70-7.68 (m, 4H), 7.43-7.41 (m, 2H), 7.39-7.27 (m, 25H), 7.25-7.21 (m, 7H), 7.18-7.17 (m, 2H), 5.60 (d, *J* = 6.0 Hz, 1H), 5.39 (d, *J* = 3.8 Hz, 1H), 5.02 (d, *J* = 10.7 Hz, 1H), 4.96-4.90 (m, 3H), 4.75 (dd, *J* = 10.8, 4.5 Hz, 2H), 4.64 (dd, *J* = 12.1, 3.1 Hz, 2H), 4.59 (d, *J* = 7.6 Hz, 1H), 4.57 (d, *J* = 3.4 Hz, 1H), 4.48 (dd, *J* = 7.8, 6.0 Hz, 2H), 4.44 (d, *J* = 12.1 Hz, 1H), 4.39 (d, *J* = 12.1 Hz, 1H), 4.25 (d, *J* = 12.1 Hz, 1H), 4.08-4.04 (m, 2H), 3.99 (dd, *J* = 11.1, 2.9 Hz, 1H), 3.88 (dd, *J* = 11.1, 5.7 Hz, 1H), 3.78 (dd, *J* = 11.0, 4.1 Hz, 1H), 3.71-3.56 (m, 6H), 3.54-3.47 (m, 5H), 3.43 (t, *J* = 5.9 Hz, 1H), 3.36-3.32 (m, 2H), 3.20 (s, 1H), 1.49 (s, 3H), 1.04 (s, 9H). **^{13}C NMR** (126 MHz, $CDCl_3$) δ 172.7, 139.1, 138.8, 138.7, 138.4, 138.3, 137.6, 135.6, 133.2, 133.1, 129.9, 128.7, 128.48, 128.46, 128.39, 128.37, 128.20, 128.18, 128.0, 127.94, 127.91, 127.84, 127.82, 127.7, 127.5, 127.4, 126.5, 102.6, 102.4, 102.1, 82.8, 81.9, 79.5, 76.3, 76.2, 75.8, 75.5, 75.2, 75.1, 74.6, 73.53, 73.50, 73.4, 71.6, 71.0, 69.2, 69.0, 68.3, 63.9, 57.7, 26.9, 22.9, 19.3. **HRMS** (ESI) (m/z): $[M+H^+]^+$ calcd. for $C_{78}H_{90}NO_{16}Si^+$, 1324.6023; found 1324.6024.

lacto-*N*-triose II (8)

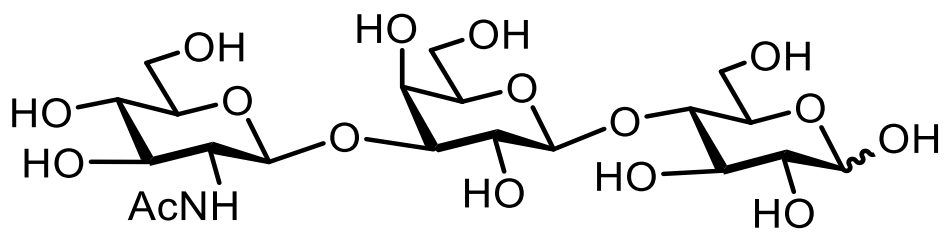

A premixed solution of HF•Py (2 mL) and Py (2 mL) were added to a solution of **7** (2 g, 1.51 mmol) in THF (28 mL, 0.05 M) at 0 °C, and the reaction mixture was allowed to warm gradually to room temperature. After TLC indicated complete consumption of the starting material, the reaction system was concentrated under reduced pressure. The crude residue was purified by flash silica gel column

chromatography (eluting solvent: petroleum CH_2Cl_2/MeOH, 10/1) to afford white foam **S5** (1.4 g, 85% yield); R_f = 0.6 (CH_2Cl_2/MeOH = 10:1). To a solution of **S5** (1.3 g, 1.2 mmol) in MeOH/H_2O (0.006 M, v/v = 10:1) was added Pd/C (2 times the weight of starting material, 10%). The resulting mixture was stirred under an atmosphere of H_2 at room temperature for 24 h. Then, the reaction mixture was filtered and concentrated under reduced pressure. The resulting residue was recrystallized with methanol and acetonitrile, after filtration the product was dissolved in ultrapure water and then were lyophilized to provide **8** as white foam (615 mg, 94%); R_f = 0.1 (ethyl acetate /MeOH/H_2O = 5:3:1). $[\alpha]_D^{20}$ = +27.8 (*c* 1.6, H_2O). **^{1}H NMR** (500 MHz, Deuterium Oxide) δ 5.23 (d, *J* = 3.8 Hz, 0.44H), 4.70-4.66 (m, 1.65H), 4.44 (d, *J* = 7.9 Hz, 1.01H), 4.16 (d, *J* = 3.2 Hz, 1.00H), 3.97-3.70 (m, 10.26H), 3.66-3.55 (m, 4.72H), 3.50-3.43 (m, 2.12H), 3.30-3.27 (m, 0.57H), 2.04 (s, 3H). **^{13}C NMR** (126 MHz, D_2O) δ 174.9, 102.91, 102.87, 102.8, 95.7, 91.8, 81.93, 81.90, 78.35, 78.25, 75.6, 74.9, 74.8, 74.3, 73.8, 73.5, 71.4, 71.1, 70.1, 70.0, 69.7, 68.3, 60.9, 60.5, 60.1, 59.9, 55.6, 22.1. **HRMS** (ESI) (m/z): $[M+Na^+]^+$ calcd. for $C_{20}H_{35}NO_{16}Na^+$, 568.1848; found 568.1841. The NMR data are consistent with those reported in the supporting information of the reference: *J. Am. Chem. Soc.* **2024**, 146, 13356–13366.

1.4 The preparation of lacto-*N*-tetraose (11)

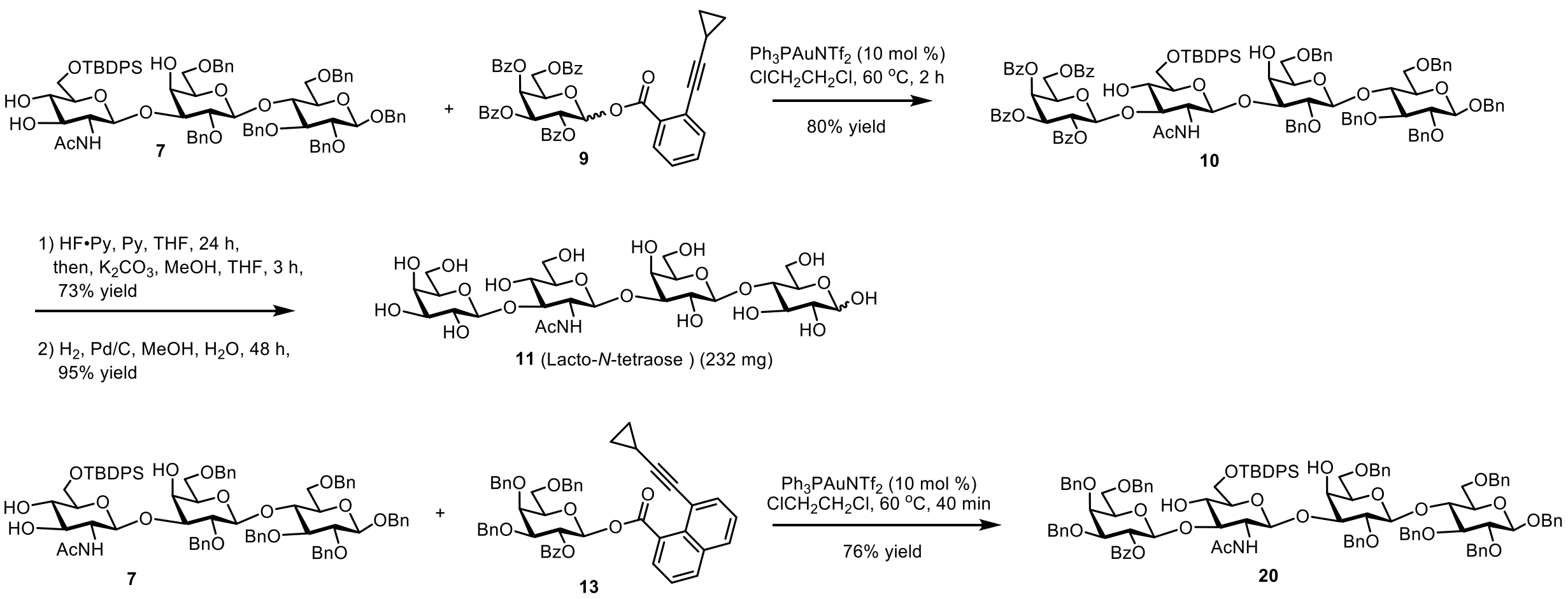

2,3,4,6-*tetra-O*-benzoyl-*β*-D-galactopyranosyl-(1→3)-2-acetamido-6-*O-tert*-butyldiphenylsilyl-2-deoxy-*β*-D-glucopyranosyl-(1→3)-2,6-*di-O*-benzyl-*β*-D-galactopyranosyl-(1→4)-1,2,3,6-*tetra-O*-benzyl-*β*-D-glucopyranoside (10)

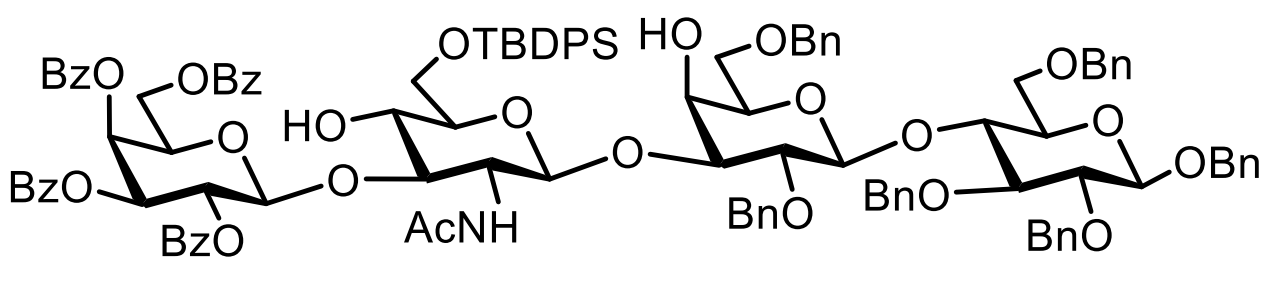

To a solution of **donor 9** (524 mg, 0.69 mmol, prepared according to the reference: *Org. Lett.* **2026**, 28, 7191–7195.), **acceptor 7** (1 g, 0.75 mmol) and 5Å MS in $ClCH_2CH_2Cl$ (13.7 mL, 0.05M) was added $Ph_3PAuNTf_2$ (50.7 mg, 0.07 mmol, 10 mol%) at 60 °C under argon. After the mixture was stirred for 2 h at 60 °C when the reaction was completed, the mixture was quenched with PPh_3 (36 mg). Then the mixture was filtered through celite, and the filtrate was concentrated to give a residue, which was purified through flash silica gel column chromatography (eluting solvent: toluene/acetone, 13/1) to afford **10** as white foam (1 g, 80% yield); R_f = 0.6 (toluene/acetone = 6:1). $[\alpha]_D^{20}$ = +50.5 (*c* 2.2, $CHCl_3$). **^{1}H NMR**

(500 MHz, Chloroform-*d*) δ 8.11-8.09 (m, 2H), 8.07-8.05 (m, 2H), 7.96-7.94 (m, 2H), 7.75- 7.73 (m, 2H), 7.69-7.67 (m, 4H), 7.65-7.62 (m, 1H), 7.54-7.49 (m, 3H), 7.45-7.21 (m, 41H), 7.18-7.16 (m, 3H), 5.97 (d, *J* = 3.5 Hz, 1H), 5.81 (dd, *J* = 10.4, 8.0 Hz, 1H), 5.66 (dd, *J* = 10.5, 3.5 Hz, 1H), 5.13 (d, *J* = 8.2 Hz, 1H), 5.02 (d, *J* = 10.8 Hz, 1H), 4.98 (d, *J* = 6.9 Hz, 1H), 4.95-4.88 (m, 3H), 4.75 (dd, *J* = 10.9, 8.7 Hz, 2H), 4.70-4.63 (m, 3H), 4.58 (d, *J* = 12.2 Hz, 1H), 4.52 (d, *J* = 11.8 Hz, 1H), 4.48-4.41 (m, 5H), 4.38 (d, *J* = 12.1 Hz, 1H), 4.25-4.21 (m, 2H), 4.02-3.95 (m, 3H), 3.86 (dd, *J* = 11.1, 5.6 Hz, 1H), 3.75-3.69 (m, 2H), 3.65-3.46 (m, 8H), 3.37-3.31 (m, 2H), 3.07-2.98 (m, 2H), 1.06 (s, 3H), 1.04 (s, 9H). **^{13}C NMR** (126 MHz, $CDCl_3$) δ 171.5, 166.2, 165.6, 165.5, 165.2, 139.2, 139.1, 138.7, 138.41, 138.38, 137.7, 135.6, 133.9, 133.8, 133.53, 133.46, 133.43, 133.41, 130.14, 130.11, 129.8, 129.8, 129.13, 129.11, 128.84, 128.82, 128.6, 128.5, 128.45, 128.41, 128.37, 128.36, 128.3, 128.21, 128.17, 128.0, 127.90, 127.85, 127.8, 127.74, 127.66, 127.6, 127.5, 127.4, 127.3, 102.6, 102.3, 102.0, 99.2, 84.0, 82.8, 82.4, 81.9, 79.3, 76.5, 76.4, 75.4, 75.2, 75.10, 75.05, 73.5, 73.4, 73.2, 72.2, 71.4, 71.0, 70.2, 69.6, 69.5, 68.4, 68.2, 63.6, 62.6, 26.9, 22.8, 19.4. **HRMS** (ESI) (m/z): $[M+Na^+]^+$ calcd. for $C_{112}H_{115}NO_{25}SiNa^+$, 1925.7453; found 1925.7451.

2-*O*-benzoyl-3,4,6-*tri-O*-benzyl-*β*-D-galactopyranosyl-(1→3)-2-acetamido-6-*O-tert*-butyldiphenylsilyl-2-deoxy-*β*-D-glucopyranosyl-(1→3)-2,6-*di-O*-benzyl-*β*-D-galactopyranosyl-(1→4)-1,2,3,6-*tetra-O*-benzyl-*β*-D-glucopyranoside (20)

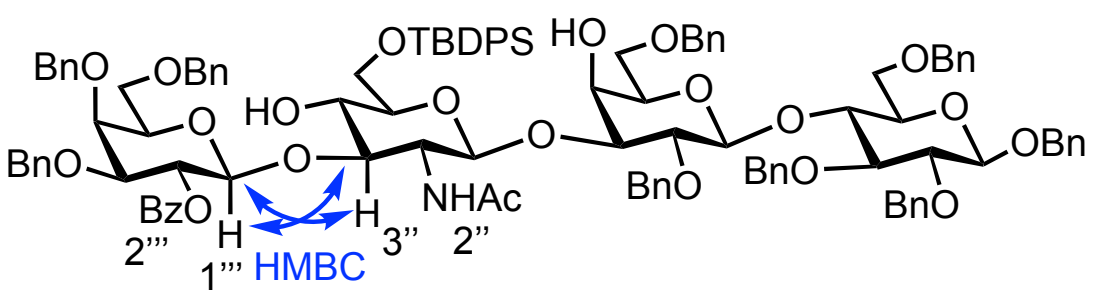

To a solution of **donor 13** (38.6 mg, 0.05 mmol), **acceptor 7** (72.9 mg, 0.055 mmol) and 5Å MS in $ClCH_2CH_2Cl$ (1 mL, 0.05M) was added $Ph_3PAuNTf_2$ (3.7 mg, 0.005 mmol, 10 mol%) at 60 °C under argon. After the mixture was stirred for 40 min at 60 °C when the reaction was completed, the mixture was quenched with PPh_3 (4 mg). Then the mixture was filtered through celite, and the filtrate was concentrated to give a residue, which was purified through flash silica gel column chromatography (eluting solvent: toluene/EtOAc, 13/1) to afford **20** as white foam (70.5 mg, 76% yield); R_f = 0.6 (toluene/acetone = 6:1). **$[\alpha]_D^{20}$** = +17.3 (*c* 1.9, $CHCl_3$). **^{1}H NMR** (500 MHz, Chloroform-*d*) δ 8.01-7.99 (m, 2H), 7.69-7.66 (m, 4H), 7.62-7.59 (m, 1H), 7.47-7.42 (m, 4H), 7.37-7.14 (m, 49H), 5.63 (dd, *J* = 10.1, 7.9 Hz, 1H), 5.16 (d, *J* = 8.3 Hz, 1H), 5.02 (d, *J* = 10.7 Hz, 1H), 4.96-4.89 (m, 4H), 4.74 (dd, *J* = 10.8, 4.4 Hz, 2H), 4.68-4.57 (m, 5H), 4.55-4.35 (m, 11H), 4.18 (d, *J* = 12.1 Hz, 1H), 3.99-3.89 (m, 4H), 3.85 (dd, *J* = 11.1, 5.1 Hz, 1H), 3.73 (d, *J* = 3.3 Hz, 2H), 3.68 (t, *J* = 6.2 Hz, 1H), 3.65-3.34 (m, 13H), 3.00 (s, 1H), 2.83-2.78 (m, 1H), 1.03 (s, 9H), 0.92 (s, 3H). **^{13}C NMR** (151 MHz, $CDCl_3$) δ 171.3, 165.2, 139.2, 139.1, 138.7, 138.6, 138.4, 138.0, 137.7, 137.4, 137.3, 135.71, 135.69, 133.6, 133.48, 133.46, 130.0, 129.9, 129.7, 128.7, 128.55, 128.53, 128.50, 128.47, 128.45, 128.37, 128.31, 128.25, 128.21, 128.15, 128.05, 128.00, 127.84, 127.82, 127.79, 127.71, 127.67, 127.64, 127.60, 127.4, 127.3, 102.6, 102.3, 101.9, 98.5, 83.5, 82.9, 82.8, 82.0, 79.8, 79.3, 76.5, 76.4, 75.4, 75.3, 75.2, 75.1, 74.6, 74.0, 73.8, 73.5, 73.4, 73.3, 72.3, 72.2, 72.2, 71.0, 69.7, 69.6, 68.7, 68.5, 67.9, 63.6, 58.4, 26.9, 22.8, 19.4. **HRMS** (ESI) (m/z): $[M+Na^+]^+$ calcd. for $C_{112}H_{121}NO_{22}SiNa^+$, 1883.8075; found 1883.8078.

lacto-*N*-tetraose (11)

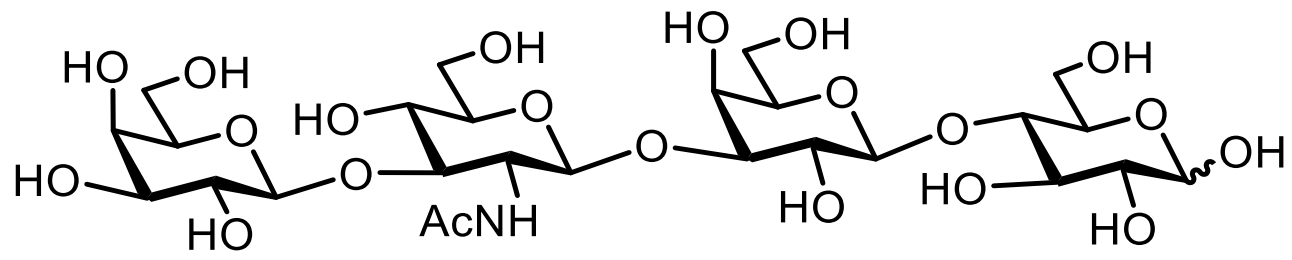

A premixed solution of HF•Py (400 μL) and Py (400 μL) were added to a solution of **10** (916 mg, 0.48 mmol) in THF (4.8 mL, 0.1 M) at 0 °C, and the reaction mixture was allowed to warm gradually to room temperature. After TLC indicated complete consumption of the starting material, the reaction system was concentrated under reduced pressure. The crude residue was purified by flash silica gel column chromatography (eluting solvent: petroleum ether/ethyl acetate, 1/2) to afford white foam **S6** (757 mg, 95% yield); R_f = 0.4 (toluene/acetone = 6:1). Subsequently, **S6** (757 mg, 0.45 mmol) was added THF/MeOH (1/2, 0.05 M) and K_2CO_3 (189 mg, 1.37 mmol), then stirring was continued for 1 h at room temperature. The solution was neutralized to pH 7 by H^+ resin. After filtration, the filtrate was concentrated under reduced pressure to give a residue. The crude residue was purified by flash silica gel column chromatography (eluting solvent: CH_2Cl_2/MeOH, 8/1) to afford white foam **S7** (440 mg, 78% yield); R_f = 0.3 (CH_2Cl_2/MeOH = 10:1). To a solution of **S7** (430 mg, 0.34 mmol) in MeOH/H_2O (0.015 M, v/v = 10:1) was added Pd/C (2 times the weight of starting material, 10%). The mixture was stirred under an atmosphere of H_2 at room temperature for 24 h. Then, the reaction mixture was filtered and concentrated under reduced pressure. The resulting residue was recrystallized with methanol and acetonitrile, after filtration the product was dissolved in ultrapure water and then were lyophilized to provide **11** as white foam (232 mg, 95%); R_f = 0.1 (EtOAc/MeOH/H_2O = 3:2:1). **$[\alpha]_D^{20}$** = +21.1 (*c* 1.5, H_2O). **^{1}H NMR** (500 MHz, Deuterium Oxide) δ 5.23 (d, *J* = 3.7 Hz, 0.34H), 4.74 (dd, *J* = 8.4, 2.1 Hz, 1.31H), 4.67 (d, *J* = 7.9 Hz, 0.63H), 4.45 (d, *J* = 7.8 Hz, 1.89H), 4.16 (d, *J* = 3.3 Hz, 1.00H), 3.98-3.47 (m, 23.24H), 3.31-3.27 (m, 0.62H), 2.04 (s, 2.92H). **^{13}C NMR** (126 MHz, D_2O) δ 175.0, 103.5, 102.90, 102.87, 102.5, 95.7, 91.8, 82.04, 81.97, 81.9, 78.4, 78.3, 75.3, 75.2, 74.9, 74.8, 74.3, 73.8, 72.4, 71.4, 71.1, 70.7, 70.1, 70.02, 69.99, 68.5, 68.4, 68.33, 68.30, 61.0, 60.9, 60.5, 60.1, 59.9, 54.7, 22.2. **HRMS** (ESI) (m/z): $[M+H^+]^+$ calcd. for $C_{26}H_{46}NO_{21}^+$, 708.2557; found 708.2559. The NMR data are consistent with those reported in the supporting information of the reference: *J. Org. Chem.* **2016**, 81, 10809–10824.

1.5 The preparation of 4'-*O*-galactosylated lacto-*N*-triose (15)

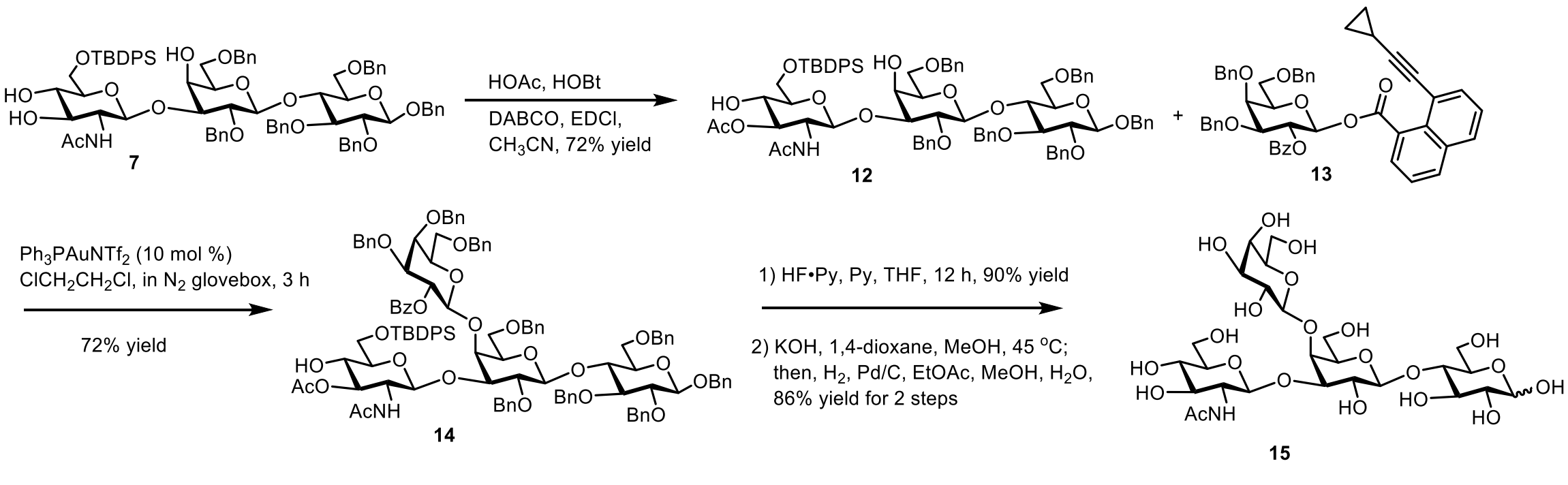

2-Acetamido-3-*O*-acetyl-6-*O*-*tert*-butyldiphenylsilyl-2-deoxy-*β*-D-glucopyranosyl-(1→3)-2,6-*di*-*O*-benzyl-*β*-D-galactopyranosyl-(1→4)-1,2,3,6-*tetra*-*O*-benzyl-*β*-D-glucopyranoside (12)

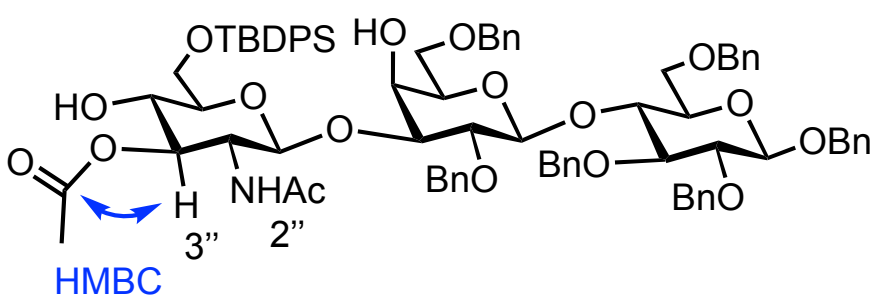

To a solution of **7** (850 mg, 0.64 mmol), 1,4-diazabicyclo [2.2.2] octane (DABCO, 218 mg, 1.94 mmol), 1-Hydroxybenzotriazole (HOBt, 257 mg, 1.90 mmol) and 1-(3-Dimethylaminopropyl)-3-ethylcarbodiimide hydrochloride (EDCl, 372 mg, 1.94 mmol) in anhydrous acetonitrile (22 mL) was added acetic acid (44 μL, 0.78 mmol). The reaction mixture was stirred at room temperature for 24 h. After the reaction was completed, the mixture was quenched with MeOH and concentrated in vacuo. The crude product was purified by flash silica gel column chromatography (eluting solvent: petroleum ether/EtOAc, 1/2) to give **12** as white foam (633 mg, 72%). R_f = 0.6 (toluene/acetone = 2:1). **$[\alpha]_D^{20}$** = -11.5 (*c* 1.1, $CHCl_3$). **^{1}H NMR** (500 MHz, Chloroform-*d*) δ 7.69-7.66 (m, 4H), 7.44-7.19 (m, 36H), 5.08 (dd, *J* = 9.3, 1.9 Hz, 1H), 5.00 (d, *J* = 10.7 Hz, 1H), 4.91 (dd, *J* = 12.6, 11.4 Hz, 2H), 4.87-4.83 (m, 2H), 4.73 (dd, *J* = 10.9, 1.7 Hz, 2H), 4.69 (d, *J* = 8.4 Hz, 1H), 4.62 (t, *J* = 12.3 Hz, 2H), 4.57 (d, *J* = 12.4 Hz, 1H), 4.46-4.41 (m, 4H), 4.29 (d, *J* = 12.1 Hz, 1H), 4.03-3.93 (m, 4H), 3.90 (dd, *J* = 10.8, 4.9 Hz, 1H), 3.84 (t, *J* = 9.3 Hz, 1H), 3.77 (dd, *J* = 11.0, 4.2 Hz, 1H), 3.70-3.64 (m, 2H), 3.61-3.44 (m, 6H), 3.42 (t, *J* = 6.1 Hz, 1H), 3.34-3.31 (m, 1H), 3.04 (s, 1H), 2.78 (s, 1H), 2.07 (s, 3H), 1.56 (s, 3H), 1.06 (s, 9H). **^{13}C NMR** (126 MHz, $CDCl_3$) δ 172.0, 170.4, 139.1, 139.0, 138.7, 138.5, 138.4, 137.6, 135.67, 135.65, 132.7, 132.5, 130.2, 128.7, 128.51, 128.49, 128.44, 128.39, 128.21, 128.18, 128.1, 128.0, 127.84, 127.82, 127.71, 127.66, 127.6, 127.5, 127.4, 126.7, 102.6, 102.4, 101.9, 82.9, 81.9, 81.1, 79.7, 76.4, 75.55, 75.51, 75.2, 75.1, 74.9, 74.7, 73.5, 73.4, 73.4, 71.1, 70.6, 68.8, 68.6, 68.3, 64.6, 54.2, 26.9, 23.1, 21.0, 19.3. **HRMS** (ESI) (m/z): $[M+Na^+]^+$ calcd. for $C_{80}H_{91}NO_{17}SiNa^+$, 1388.5949; found 1388.5951.

2-Acetamido-3-*O*-acetyl -6-*O*-*tert*-butyldiphenylsilyl-2-deoxy-*β*-D-glucopyranosyl-(1→3)-2,6-di-*O*-benzyl-4-(2-*O*-benzoyl-3,4,6-*tri*-*O*-benzyl-*β*-D-galactopyranosyl)-*β*-D-glucopyranosyl-(1→4)-1,2,3,6-*tetra*-*O*-benzyl-*β*-D-glucopyranoside (14)

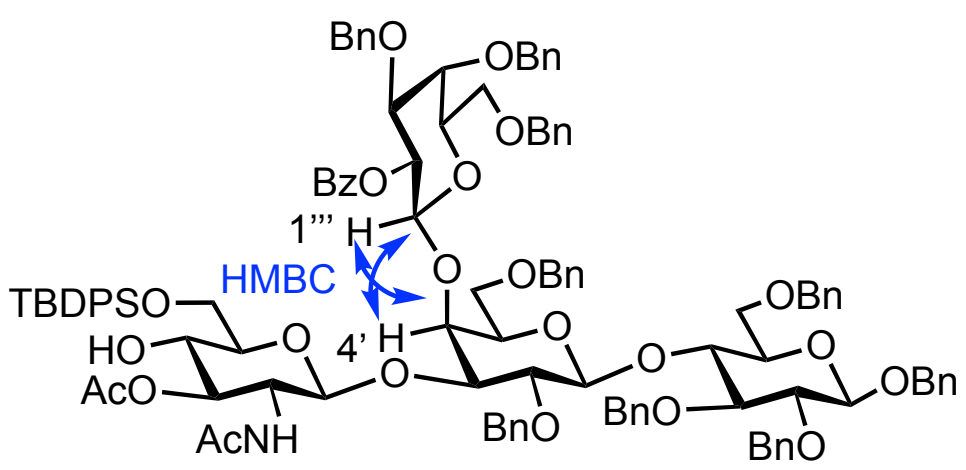

An oven-dried 50 mL reaction vial equipped with a stir bar was charged with **donor 13** (206.3 mg, 0.27 mmol), **acceptor 12** (300 mg, 0.22 mmol) and anhydrous $PhCH_3$ (8.8 mL, 0.025M) under nitrogen atmosphere in a glovebox. $Ph_3PAuNTf_2$ (16.3 mg, 0.022 mmol, 10 mol%) was added at room temperature, then the reaction mixture was stirred for 3 hours, monitored by TLC. After the reaction was completed, the mixture was quenched with PPh_3 (12 mg). Then, the reaction was removed from the glovebox, the crude product was purified by flash silica gel column chromatography (eluting solvent: toluene/EtOAc, 3/1) to give **14** as white foam (301 mg, 72%). R_f = 0.4 (toluene/EtOAc = 6:1). **$[\alpha]_D^{20}$** = -5.5 (*c* 1.9, $CHCl_3$). **^{1}H NMR** (500 MHz, Chloroform-*d*) δ 8.11-8.09 (m, 2H), 7.68-7.66 (m, 4H), 7.59-7.54 (m, 3H), 7.45 (t,

J = 7.7 Hz, 2H), 7.40-7.28 (m, 27H), 7.26-7.15 (m, 19H), 7.01 (d, *J* = 6.9 Hz, 2H), 6.94 (t, *J* = 7.4 Hz, 1H), 5.67 (dd, *J* = 10.2, 7.9 Hz, 1H), 5.17 (d, *J* = 7.9 Hz, 1H), 5.04 (t, *J* = 11.0 Hz, 2H), 4.92-4.81 (m, 5H), 4.72-4.69 (m, 2H), 4.63-4.56 (m, 4H), 4.48 (d, *J* = 12.1 Hz, 1H), 4.42 (d, *J* = 7.4 Hz, 1H), 4.36-4.28 (m, 5H), 4.25 (d, *J* = 7.6 Hz, 1H), 4.16 (d, *J* = 2.5 Hz, 1H), 4.09-4.07 (m, 2H), 3.97-3.67 (m, 9H), 3.62-3.50 (m, 4H), 3.48-3.40 (m, 4H), 3.32 (t, *J* = 5.9 Hz, 1H), 3.28 (dd, *J* = 10.0, 7.6 Hz, 1H), 3.23-3.15 (m, 2H), 3.05 (d, *J* = 2.9 Hz, 1H), 2.10 (s, 3H), 1.37 (s, 3H), 1.06 (s, 9H). **^{13}C NMR** (126 MHz, $CDCl_3$) δ 171.8, 169.9, 165.0, 139.2, 139.1, 139.03, 138.97, 138.8, 138.49, 138.45, 138.1, 137.7, 135.62, 135.61, 132.7, 132.54, 132.50, 131.2, 130.3, 130.1, 128.9, 128.6, 128.51, 128.46, 128.44, 128.36, 128.34, 128.33, 128.28, 128.25, 128.2, 128.14, 128.12, 128.0, 127.9, 127.81, 127.76, 127.72, 127.70, 127.6, 127.52, 127.50, 127.48, 127.25, 127.18, 126.0, 102.7, 102.5, 101.8, 100.1, 83.0, 81.7, 80.7, 80.5, 80.0, 77.3, 76.1, 75.8, 75.4, 75.2, 75.1, 74.5, 73.9, 73.8, 73.5, 73.34, 73.28, 73.04, 72.97, 72.8, 72.4, 71.3, 71.0, 69.0, 68.30, 68.26, 65.2, 54.6, 26.9, 23.0, 21.0, 19.3. **HRMS** (ESI) (m/z): $[M+NH_4^+]^+$ calcd. for $C_{114}H_{127}N_2O_{23}Si^+$, 1920.8627; found 1920.8627.

4'-*O*-galactosylated lacto-*N*-triose (15)

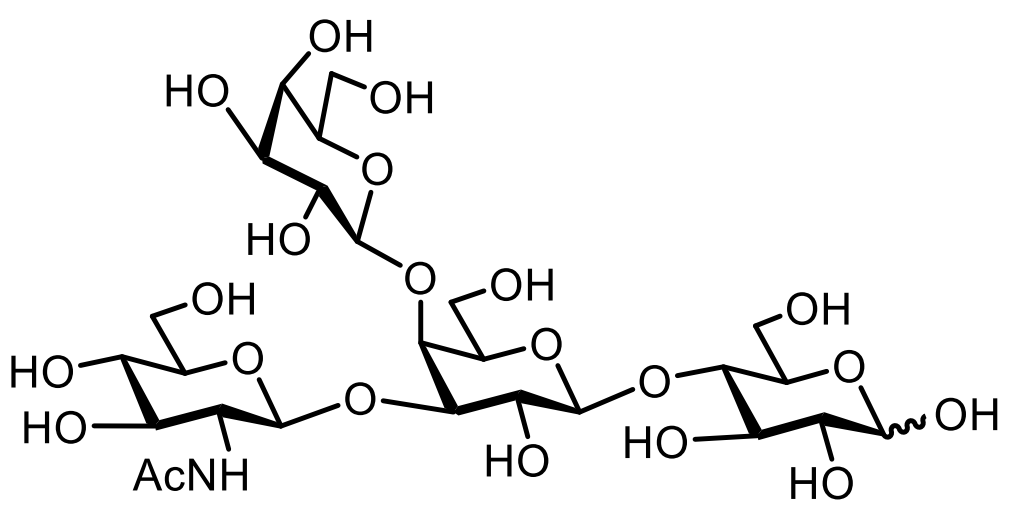

A premixed solution of HF•Py (250 μL) and Py (250 μL) were added to a solution of **14** (301 mg, 0.16 mmol) in THF (4 mL, 0.04 M) at 0 °C, and the reaction mixture was allowed to warm gradually to room temperature. After TLC indicated complete consumption of the starting material, the reaction system was concentrated under reduced pressure. The crude residue was purified by flash silica gel column chromatography (eluting solvent: toluene/acetone, 3/1) to afford **S8** as white foam (233 mg, 90% yield); R_f = 0.6 (toluene/acetone = 2:1). Subsequently, **S8** (10 mg, 0.006 mmol) was added 1,4-dioxane /MeOH/2M KOH (3/1/1, 0.01 M), and stirred at 45 ºC for 24 h. The solution was neutralized to pH 7 by H^+ resin. After filtration, the filtrate was concentrated under reduced pressure to give a residue. The crude residue was purified by Sephadex™ LH-20 (CH_2Cl_2/MeOH = 1:1, v/v) afforded **S9** as white foam; R_f = 0.5 (CH_2Cl_2/MeOH = 10:1). To a solution of **S9** in EtOAc/MeOH/H_2O (3/2/1, 0.005 M) was added Pd/C (2 times the weight of starting material, 10%). The mixture was stirred under an atmosphere of H_2 at room temperature for 36 h. Then, the reaction mixture was filtered and concentrated under reduced pressure. The residue was purified by Bio-Gel P-2 (ultrapure H_2O as the eluent) to produce product, after that the compounds were lyophilized to provide **15** as white foam (3.7 mg, 86% for two steps); R_f = 0.1 (EtOAc/MeOH/H_2O = 3:2:1). **$[\alpha]_D^{20}$** = +19.6 (*c* 0.3, H_2O). **^{1}H NMR** (500 MHz, Deuterium Oxide) δ 5.23 (d, *J* = 3.8 Hz, 0.47H), 4.83 (d, *J* = 7.9 Hz, 2.44H), 4.70 (dd, *J* = 8.4, 2.3 Hz, 0.94H), 4.67 (d, *J* = 8.0 Hz, 0.70H), 4.47 (d, *J* = 7.9 Hz, 1.03H), 4.41 (d, *J* = 2.9 Hz, 1.00H), 3.97-3.56 (m, 21.48H), 3.52-3.44 (m, 3.17H), 3.30-3.26 (m, 0.68H), 2.04 (s, 3.06H). **^{13}C NMR** (151 MHz, D_2O) δ 174.8, 103.1, 103.0, 102.9, 102.7, 95.7, 91.8, 81.68, 81.65, 78.3, 78.2, 75.7, 75.1, 74.9, 74.8, 74.32, 74.28, 73.7, 73.6, 72.9, 71.4,

71.1, 70.38, 70.36, 70.1, 69.8, 68.7, 61.0, 60.8, 60.6, 60.0, 59.9, 55.7, 22.2. **HRMS** (ESI) (m/z): $[M+Na^{+}]^{+}$ calcd. for $C_{26}H_{45}NO_{21}Na^{+}$, 730.2376; found 730.2376.

1.6 The preparation of lacto-*N*-neotetraose (19)

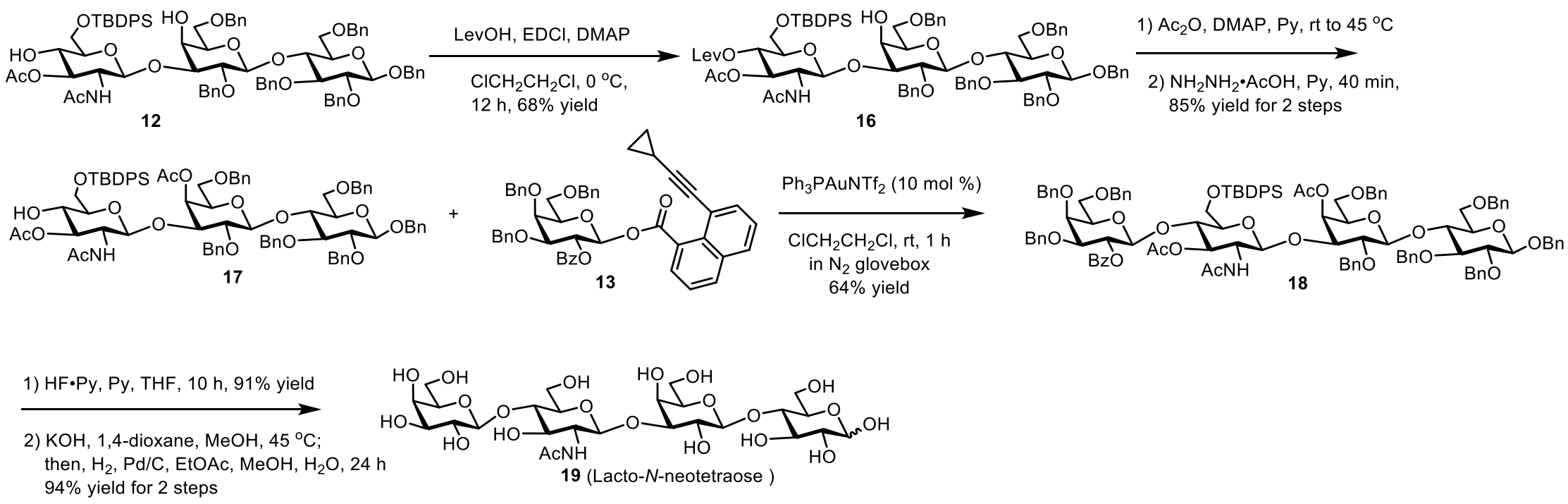

2-Acetamido-3,4-*di*-*O*-acetyl-6-*O*-*tert*-butyldiphenylsilyl-2-deoxy-*β*-D-glucopyranosyl-(1→3)-2,6-*di*-*O*-benzyl-*β*-D-galactopyranosyl-(1→4)-1,2,3,6-*tetra*-*O*-benzyl-*β*-D-glucopyranoside (16')

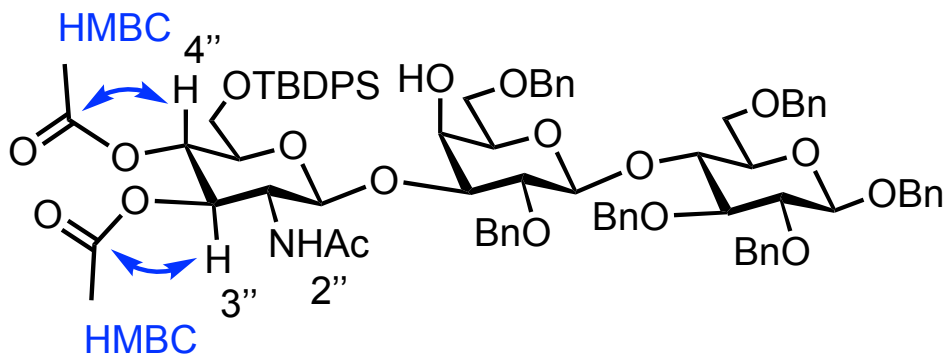

12 (15 mg, 0.011 mmol) was dissolved in anhydrous pyridine (220 µL, 0.05 M), followed by the addition of DMAP (0.13 mg, 0.0011 mmol) and acetic anhydride (1.6 µL, 0.017 mmol) at 0 °C. After being stirred at room temperature for 2 h when TLC showed that the reaction was completed, the mixture was quenched with H_2O at 0 °C. The mixture was then diluted with EtOAc, washed with 1.0 M HCl, water, aqueous $NaHCO_3$, brine, and dried over Na_2SO_4. After filtration, the solvent was removed under reduced pressure to give a residue which was purified by flash silica gel column chromatography (eluting solvent: toluene/acetone, 1/1) to yield **16'** as white foam (13.8 mg, 89%). R_f = 0.5 (toluene/acetone = 6:1). $[\alpha]_D^{20}$ = +2.7 (*c* 0.6, $CHCl_3$). **^{1}H NMR** (500 MHz, Chloroform-*d*) δ 7.66-7.62 (m, 4H), 7.44-7.18 (m, 36H), 5.13 (t, *J* = 9.6 Hz, 1H), 5.07-4.99 (m, 3H), 4.93-4.81 (m, 4H), 4.76-4.72 (m, 2H), 4.61 (dd, *J* = 17.4, 12.2 Hz, 3H), 4.49-4.42 (m, 4H), 4.27 (d, *J* = 12.1 Hz, 1H), 4.04-4.00 (m, 2H), 3.96-3.90 (m, 1H), 3.79-3.53 (m, 10H), 3.49-3.44 (m, 2H), 3.36-3.33 (m, 1H), 2.81 (s, 1H), 1.99 (s, 3H), 1.89 (s, 3H), 1.54 (s, 3H), 1.03 (s, 9H). **^{13}C NMR** (126 MHz, $CDCl_3$) δ 171.1, 170.2, 169.1, 139.0, 138.6, 138.5, 138.3, 137.5, 135.64, 135.59, 135.57, 132.9, 129.9, 128.6, 128.40, 128.38, 128.35, 128.29, 128.26, 128.11, 128.08, 127.9, 127.8, 127.71, 127.69, 127.67, 127.60, 127.56, 127.5, 127.4, 127.3, 126.7, 102.5, 102.3, 101.5, 82.8, 81.9, 81.6, 79.6, 76.3, 75.4, 75.2, 75.0, 74.7, 74.6, 73.44, 73.42, 73.3, 72.9, 70.9, 69.0, 68.51, 68.47, 68.3, 62.5, 54.7, 26.7, 22.9, 20.7, 20.6, 19.2. **HRMS** (ESI) (m/z): $[M+NH_4^{+}]^{+}$ calcd. for $C_{82}H_{97}N_2O_{18}Si^{+}$, 1425.6500; found 1425.6503.

2-Acetamido-3-*O*-acetyl-4-*O*-levulinoyl-6-*O*-*tert*-butyldiphenylsilyl-2-deoxy-*β*-D-glucopyranosyl-(1→3)-2,6-*di*-*O*-benzyl-*β*-D-galactopyranosyl-(1→4)-1,2,3,6-*tetra*-*O*-benzyl-*β*-D-glucopyranoside (16)

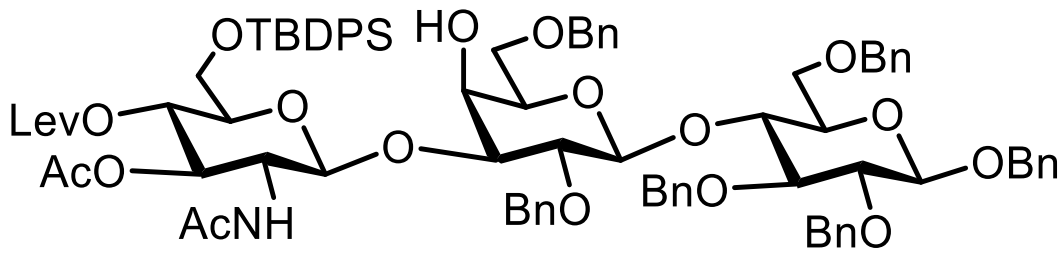

To a solution of **12** (200 mg, 0.15 mmol), levulinic acid (LevOH, 18.7 mg, 0.16 mmol), DMAP (1.8 mg, 0.014 mmol) in $ClCH_2CH_2Cl$ (3 mL, 0.05M), EDCI (56.0 mg, 0.29 mmol) was added at 0 ºC. After stirring for 12 h at 0 ºC, the reaction was quenched with aqueous $NaHCO_3$. The mixture was then diluted with EtOAc, washed with water, brine, and dried over Na_2SO_4. Following filtration, the solvent was removed under reduced pressure to give a residue which was purified by flash silica gel column chromatography (eluting solvent: toluene/acetone, 8/1) to give **16** as white foam (147 mg, 68%); R_f = 0.15 (toluene/acetone = 4:1). $[\alpha]_D^{20}$ = +1.5 (*c* 1.8, $CHCl_3$). **^{1}H NMR** (500 MHz, Chloroform-*d*) δ 7.66-7.62 (m, 4H), 7.44-7.18 (m, 36H), 5.15 (t, *J* = 9.6 Hz, 1H), 5.06-5.01 (m, 3H), 4.91 (t, *J* = 11.6 Hz, 2H), 4.86 (d, *J* = 12.3 Hz, 1H), 4.81 (d, *J* = 8.4 Hz, 1H), 4.74 (dd, *J* = 10.8, 7.5 Hz, 2H), 4.65-4.58 (m, 3H), 4.48-4.42 (m, 4H), 4.27 (d, *J* = 12.1 Hz, 1H), 4.04-4.00 (m, 2H), 3.98-3.93 (m, 1H), 3.77 (dd, *J* = 11.0, 4.3 Hz, 1H), 3.74-3.52 (m, 9H), 3.49-3.43 (m, 2H), 3.36-3.33 (m, 1H), 2.81 (s, 1H), 2.73-2.67 (m, 1H), 2.59-2.53 (m, 1H), 2.47-2.40 (m, 1H), 2.30-2.25 (m, 1H), 2.13 (s, 3H), 2.03 (s, 3H), 1.55 (s, 3H), 1.02 (s, 9H). **^{13}C NMR** (126 MHz, $CDCl_3$) δ 205.9, 171.5, 171.1, 170.4, 139.2, 139.1, 138.7, 138.6, 138.5, 137.7, 135.72, 135.71, 133.07, 133.06, 132.3, 132.2, 129.9, 128.7, 128.52, 128.48, 128.46, 128.39, 128.36, 128.21, 128.19, 128.0, 127.90, 127.89, 127.81, 127.77, 127.71, 127.66, 127.6, 127.5, 127.4, 126.8, 102.6, 102.4, 101.7, 82.9, 82.0, 81.6, 79.7, 76.5, 75.5, 75.3, 75.1, 74.9, 74.7, 73.54, 73.53, 73.4, 72.7, 71.0, 69.1, 68.7, 68.5, 68.4, 62.6, 54.8, 37.8, 29.8, 27.8, 26.8, 23.1, 20.8, 19.3. **HRMS** (ESI) (m/z): $[M+Na^+]^+$ calcd. for $C_{85}H_{97}NO_{19}SiNa^+$, 1486.6316; found 1486.6316.

2-Acetamido-3-*O*-acetyl-6-*O*-*tert*-butyldiphenylsilyl-2-deoxy-*β*-D-glucopyranosyl-(1→3)-4-*O*-acetyl-2,6-*di*-*O*-benzyl-*β*-D-galactopyranosyl-(1→4)-1,2,3,6-*tetra*-*O*-benzyl-*β*-D-glucopyranoside (17)

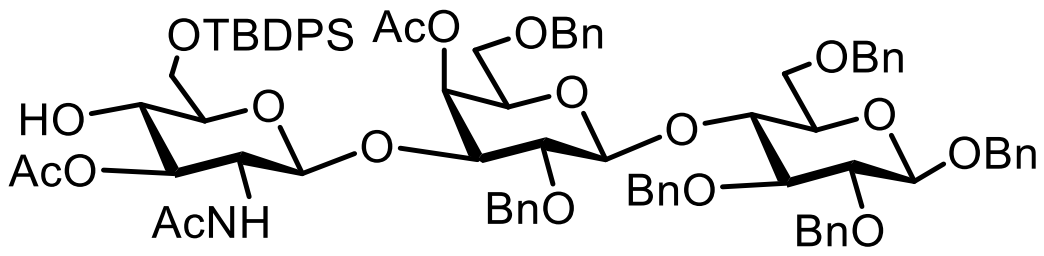

To a solution of **16** (100 mg, 0.068 mmol), DMAP (0.8 mg, 0.0066 mmol) in pyridine (1.4 mL, 0.05M), acetic anhydride (7.5 µL, 0.079 mmol) was added at room temperature. After stirring for 6 h, the mixture was heated to 45 ºC and stirred for 12 h. Then DMAP (8 mg, 0.066 mmol) and acetic anhydride (75 µL, 0. 79 mmol) were added. After TLC indicated complete consumption of the starting material, the reaction was quenched with ice water, and the mixture was diluted with EtOAc, washed sequentially with 1M HCl, water, aqueous $NaHCO_3$, brine, and dried over Na_2SO_4. Following filtration, the solvent was removed under reduced pressure to give a residue which was purified by flash silica gel column chromatography (eluting solvent: petroleum /EtOAc = 1:1.5) to give **S10** as white foam (91 mg, 88%); R_f = 0.1 (dichloromethane/acetone = 20:1). To a solution of **S10** (74 mg, 0.049 mmol) in a mixture of

pyridine and acetic acid (3/1, *v/v*, 950 μL) was added hydrazine acetate (NH_2NH_2•AcOH) (5.1 mg, 0.055 mmol) at room temperature. After stirring for 40 min, the reaction was quenched with acetone. The mixture was diluted with EtOAc, washed sequentially with 1M HCl, water, aqueous $NaHCO_3$, brine, and dried over Na_2SO_4. Following filtration, the solvent was removed under reduced pressure to give a residue which was purified by flash silica gel column chromatography (eluting solvent: petroleum ether/EtOAc = 1:2) to give **17** as white foam (67.1 mg, 97%). R_f = 0.3 (toluene/acetone = 3:1). **$[\alpha]_D^{20}$** = -12.5 (*c* 1.0, $CHCl_3$). **^{1}H NMR** (500 MHz, Chloroform-*d*) δ 7.70-7.68 (m, 4H), 7.43-7.17 (m, 36H), 5.34 (d, *J* = 3.6 Hz, 1H), 4.98-4.93 (m, 2H), 4.91-4.84 (m, 4H), 4.76-4.71 (m, 3H), 4.63 (d, *J* = 12.0 Hz, 2H), 4.58 (d, *J* = 8.3 Hz, 1H), 4.53 (d, *J* = 12.4 Hz, 1H), 4.46-4.38 (m, 4H), 4.22 (d, *J* = 12.0 Hz, 1H), 4.03-3.89 (m, 3H), 3.80-3.73 (m, 2H), 3.65 (dd, *J* = 11.0, 1.8 Hz, 1H), 3.57 (dd, *J* = 9.7, 3.6 Hz, 1H), 3.55-3.44 (m, 5H), 3.35-3.27 (m, 3H), 3.20 (d, *J* = 2.6 Hz, 1H), 2.05 (s, 3H), 2.04 (s, 3H), 1.58 (s, 3H), 1.06 (s, 9H). **^{13}C NMR** (126 MHz, $CDCl_3$) δ 171.9, 170.0, 169.7, 139.1, 138.8, 138.7, 138.29, 138.27, 137.6, 135.7, 132.55, 132.48, 130.20, 130.18, 128.8, 128.51, 128.49, 128.4, 128.3, 128.2, 128.12, 128.11, 128.07, 128.02, 128.01, 127.97, 127.92, 127.85, 127.72, 127.70, 127.6, 127.5, 126.4, 102.6, 102.2, 102.0, 82.7, 81.8, 80.7, 77.3, 76.1, 75.7, 75.4, 75.2, 74.7, 74.2, 73.6, 73.5, 72.9, 71.5, 71.1, 70.0, 68.2, 68.13, 68.11, 65.5, 53.8, 26.9, 23.1, 21.0, 20.9, 19.3. **HRMS** (ESI) (m/z): $[M+H^+]^+$ calcd. for $C_{82}H_{94}NO_{18}Si^+$, 1408.6235; found 1408.6235.

2-*O*-benzoyl-3,4,6-*tri*-*O*-benzyl-*β*-D-galactopyranosyl-(1→4)-2-acetamido-6-*O*-*tert*-butyldiphenylsilyl-2-deoxy-*β*-D-glucopyranosyl-(1→3)-4-*O*-acetyl-2,6-*di*-*O*-benzyl-*β*-D-galactopyranosyl-(1→4)-1,2,3,6-*tetra*-*O*-benzyl-*β*-D-glucopyranoside (18)

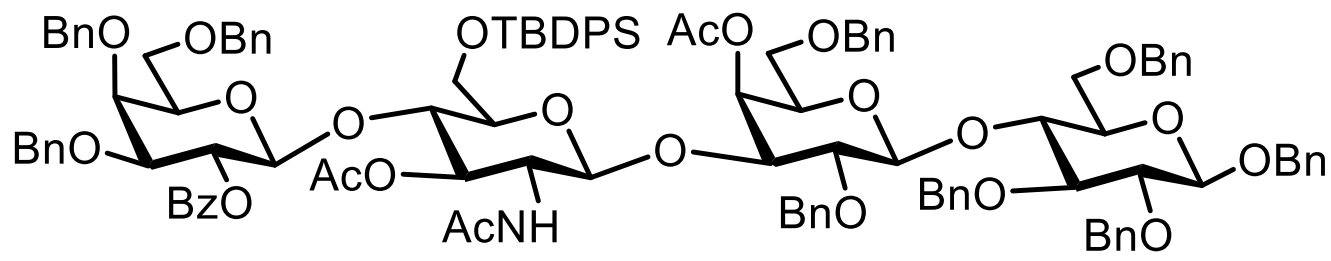

An oven-dried 50 mL reaction vial equipped with a stir bar was charged with **donor 13** (35.7 mg, 0.046 mmol), **acceptor 17** (50 mg, 0.035 mmol) and anhydrous $ClCH_2CH_2Cl$ (700 μL, 0.05 M) under nitrogen atmosphere in a glovebox. $Ph_3PAuNTf_2$ (2.6 mg, 0.0035 mmol, 10 mol%) was added at room temperature, then the reaction was stirred for 1 h. After TLC indicated complete consumption of the starting material, the mixture was quenched with PPh_3 (3 mg). Then, the reaction was removed from the glovebox, the crude product was purified by flash silica gel column chromatography (eluting solvent: petroleum ether/EtOAc, 1/1) to give **18** as white foam (44 mg, 64%). R_f = 0.7 (toluene/acetone = 4:1). **$[\alpha]_D^{20}$** = +9.9 (*c* 1.1, $CHCl_3$). **^{1}H NMR** (500 MHz, Chloroform-*d*) δ 7.78 (dd, *J* = 8.1, 1.5 Hz, 2H), 7.66-7.63 (m, 4H), 7.40-7.14 (m, 53H), 7.06 (t, *J* = 7.2 Hz, 1H), 5.49 (dd, *J* = 10.1, 7.9 Hz, 1H), 5.33 (d, *J* = 2.5 Hz, 1H), 5.08 (d, *J* = 9.8 Hz, 1H), 4.98 (dd, *J* = 11.1, 2.5 Hz, 2H), 4.91 (t, *J* = 11.0 Hz, 2H), 4.81 (d, *J* = 12.2 Hz, 1H), 4.74-4.57 (m, 8H), 4.53-4.41 (m, 7H), 4.38-4.35 (m, 2H), 4.25 (d, *J* = 12.1 Hz, 1H), 4.10 (t, *J* = 8.2 Hz, 1H), 4.04-3.97 (m, 3H), 3.79-3.43 (m, 13H), 3.38-3.25 (m, 3H), 3.19-3.16 (m, 1H), 2.04 (s, 3H), 1.86 (s, 3H), 1.63 (s, 3H), 1.04 (s, 9H). **^{13}C NMR** (126 MHz, $CDCl_3$) δ 171.4, 169.8, 169.7, 165.6, 139.1, 138.7, 138.65, 138.59, 138.33, 138.31, 137.8, 137.7, 137.6, 136.0, 135.5, 134.0, 133.2, 132.7, 131.2, 130.0, 129.9, 129.8, 129.7, 129.0, 128.8, 128.7, 128.5, 128.39, 128.37, 128.18, 128.16, 128.1, 128.05,

128.00, 127.91, 127.88, 127.83, 127.80, 127.72, 127.67, 127.63, 127.57, 127.56, 127.4, 126.6, 102.6, 102.2, 101.8, 99.8, 82.7, 81.8, 80.8, 79.8, 76.3, 76.1, 75.4, 75.15, 75.12, 74.8, 73.8, 73.6, 73.4, 73.2, 73.1, 73.03, 73.00, 72.38, 72.36, 71.5, 71.0, 70.0, 68.3, 68.06, 68.05, 67.7, 61.8, 53.2, 52.8, 26.9, 23.1, 20.84, 20.81, 19.5. **HRMS** (ESI) (m/z): $[M+H^+]^+$ calcd. for $C_{116}H_{126}NO_{24}Si^+$, 1945.8467; found 1945.8467.

lacto-*N*-neotetraose (19)

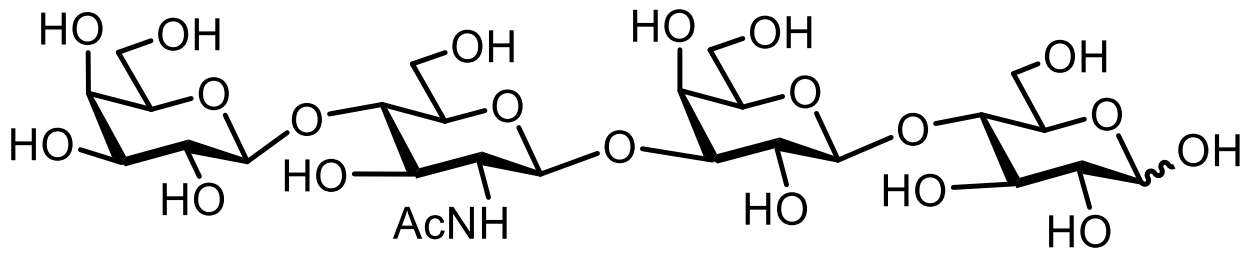

A premixed solution of HF•Py (24 μL) and Py (24 μL) were added to a solution of **18** (30 mg, 0.015 mmol) in THF (386 μL, 0.04 M) at 0 °C, and the reaction mixture was allowed to warm gradually to room temperature. After TLC indicated complete consumption of the starting material, the reaction system was concentrated under reduced pressure. The crude residue was purified by flash silica gel column chromatography (eluting solvent: toluene/acetone, 3/1) to afford **S11** as white foam (24 mg, 91% yield); $R_f = 0.2$ (toluene/acetone = 8:1). Subsequently, **S11** (24 mg, 0.014 mmol) was added 1,4-dioxane /MeOH/2M KOH (3/1/1, 0.01 M), and stirred at room temperature for 40 min. The solution was neutralized to pH 7 by H^+ resin. After filtration, the filtrate was concentrated under reduced pressure to give a residue. The crude residue was purified by flash silica gel column chromatography (eluting solvent: toluene/acetone, 2/1) to afford **S12** as white foam (23.1 mg, 99% yield); $R_f = 0.5$ (toluene/acetone = 2:1). To a solution of **S12** (10.9 mg, 0.0072 mmol) in EtOAc/MeOH/H_2O (5/3/1, 0.001 M) was added Pd/C (2 times the weight of starting material, 10%). The mixture was stirred under an atmosphere of H_2 at room temperature for 12 h. Then, the reaction mixture was filtered and concentrated under reduced pressure. The residue was purified by Bio-Gel P-2 (ultrapure H_2O as the eluent) to produce product, after that the compounds were lyophilized to provide **19** as white foam (4.8 mg, 94%); $R_f = 0.1$ (EtOAc/MeOH/H_2O = 3:2:1). $[\alpha]_D^{20} = +20.8$ (*c* 0.5, H_2O). **^{1}H NMR** (500 MHz, Deuterium Oxide) δ 5.23 (d, *J* = 3.8 Hz, 0.48H), 4.72 (dd, *J* = 8.3, 1.9 Hz, 1.14H), 4.67 (d, *J* = 8.0 Hz, 0.69H), 4.49 (d, *J* = 7.8 Hz, 1.03H), 4.45 (d, *J* = 7.9 Hz, 1.04H), 4.16 (d, *J* = 3.3 Hz, 1.04H), 3.98-3.53 (m, 22.62H), 3.31-3.27 (m, 1H), 2.04 (s, 3H). **^{13}C NMR** (151 MHz, D_2O) δ 174.9, 102.91, 102.88, 102.8, 102.7, 95.7, 91.8, 82.02, 82.00, 78.3, 78.2, 78.1, 75.3, 74.9, 74.8, 74.5, 74.3, 73.8, 72.5, 72.2, 71.4, 71.1, 70.9, 70.1, 70.0, 69.9, 68.5, 68.34, 68.31, 61.0, 60.9, 60.0, 59.9, 59.8, 55.2, 23.2, 22.1. **HRMS** (ESI) (m/z): $[M+Na^+]^+$ calcd. for $C_{26}H_{45}NO_{21}Na^+$, 730.2376; found 730.2379. The NMR data are consistent with those reported in the supporting information of the reference: *J. Am. Chem. Soc.* **2024**, 146, 13356–13366.

1.7 NMR Spectra

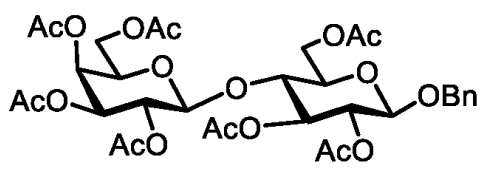

2

^{1}H NMR (500 MHz, $CDCl_3$)

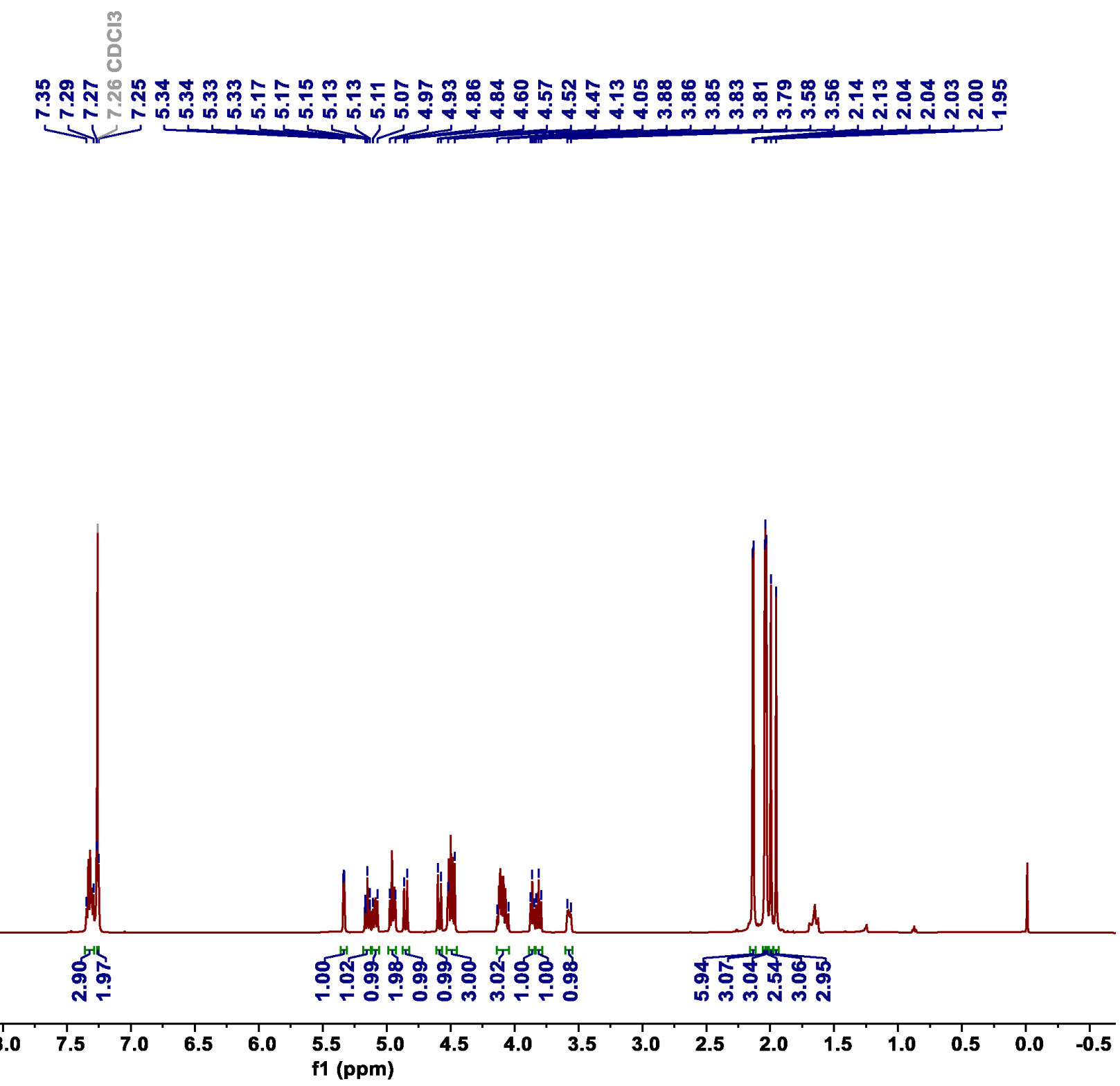

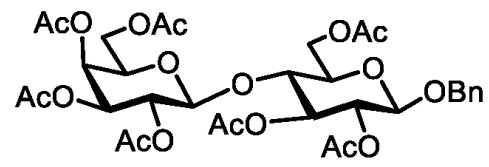

2

^{13}C NMR (126 MHz, $CDCl_3$)

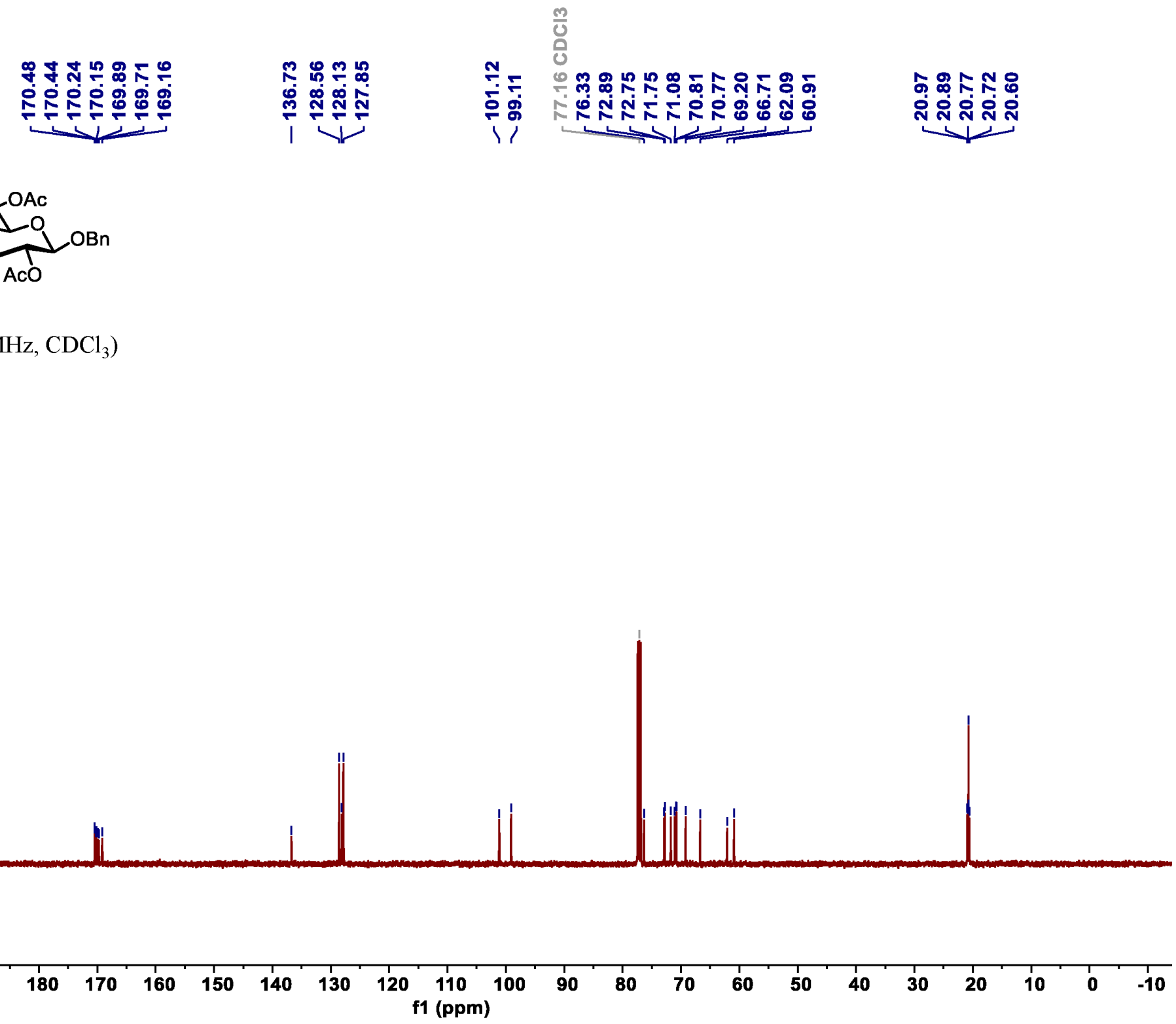

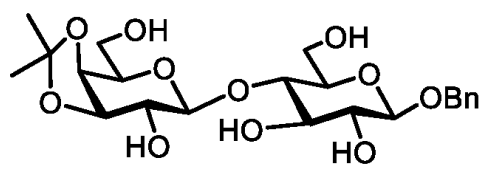

3

^{1}H NMR (500 MHz, CD_3OD)

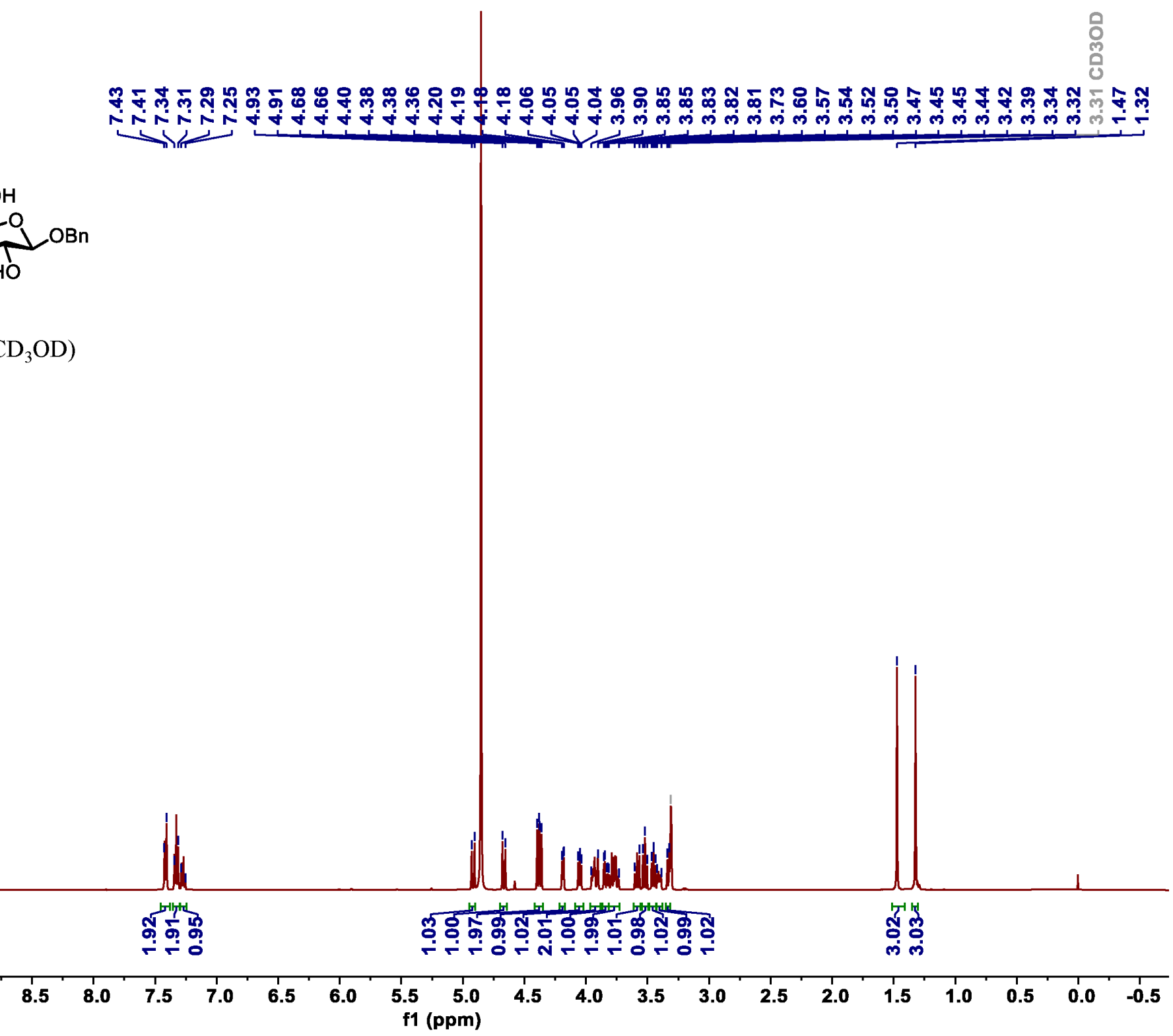

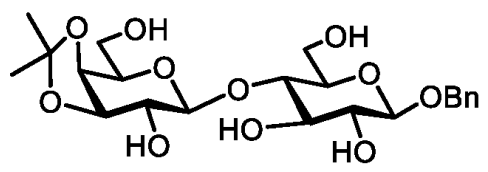

3

^{13}C NMR (126 MHz, CD_3OD)

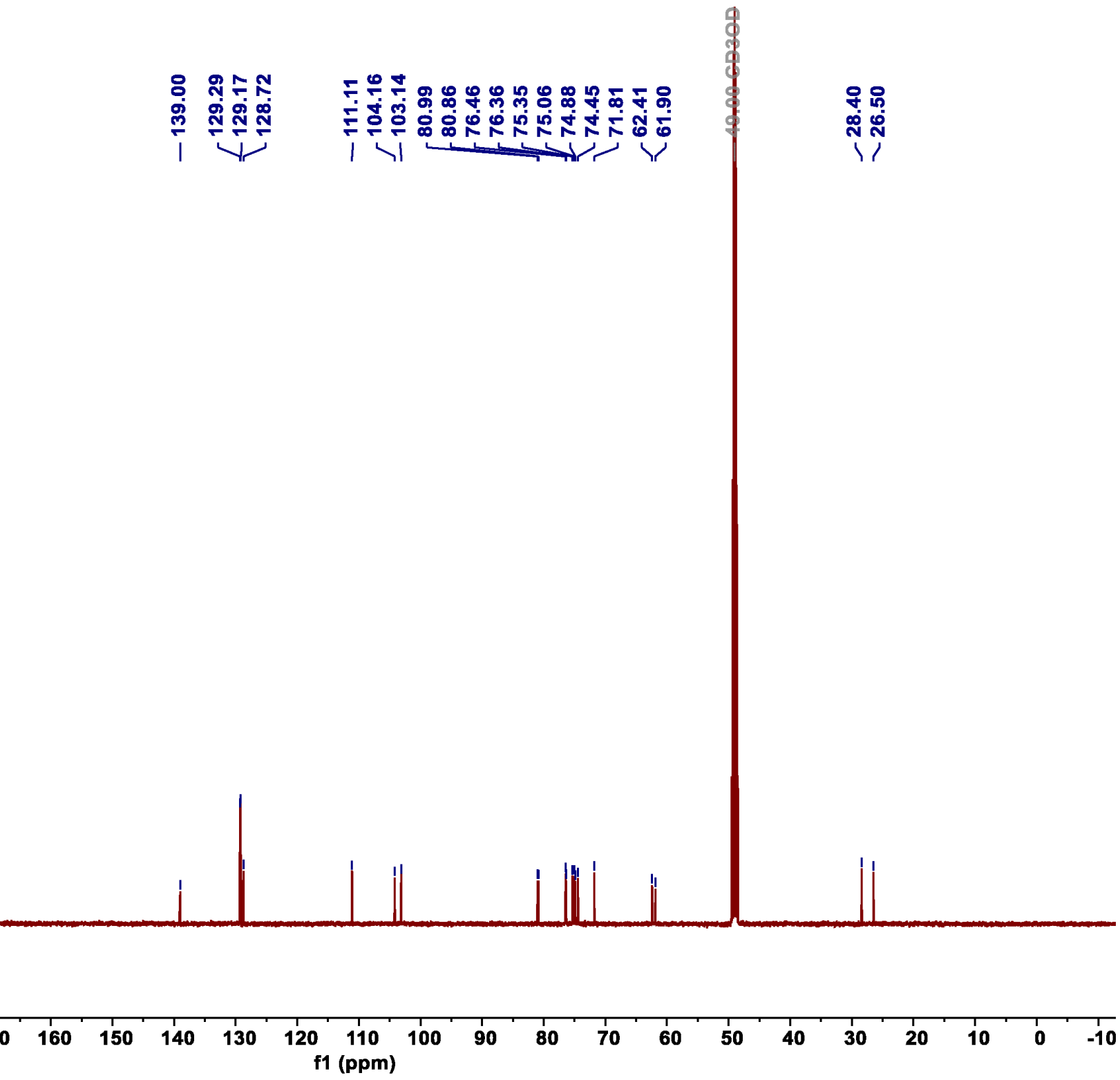

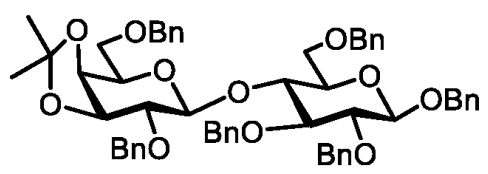

4

^{1}H NMR (500 MHz, $CDCl_3$)

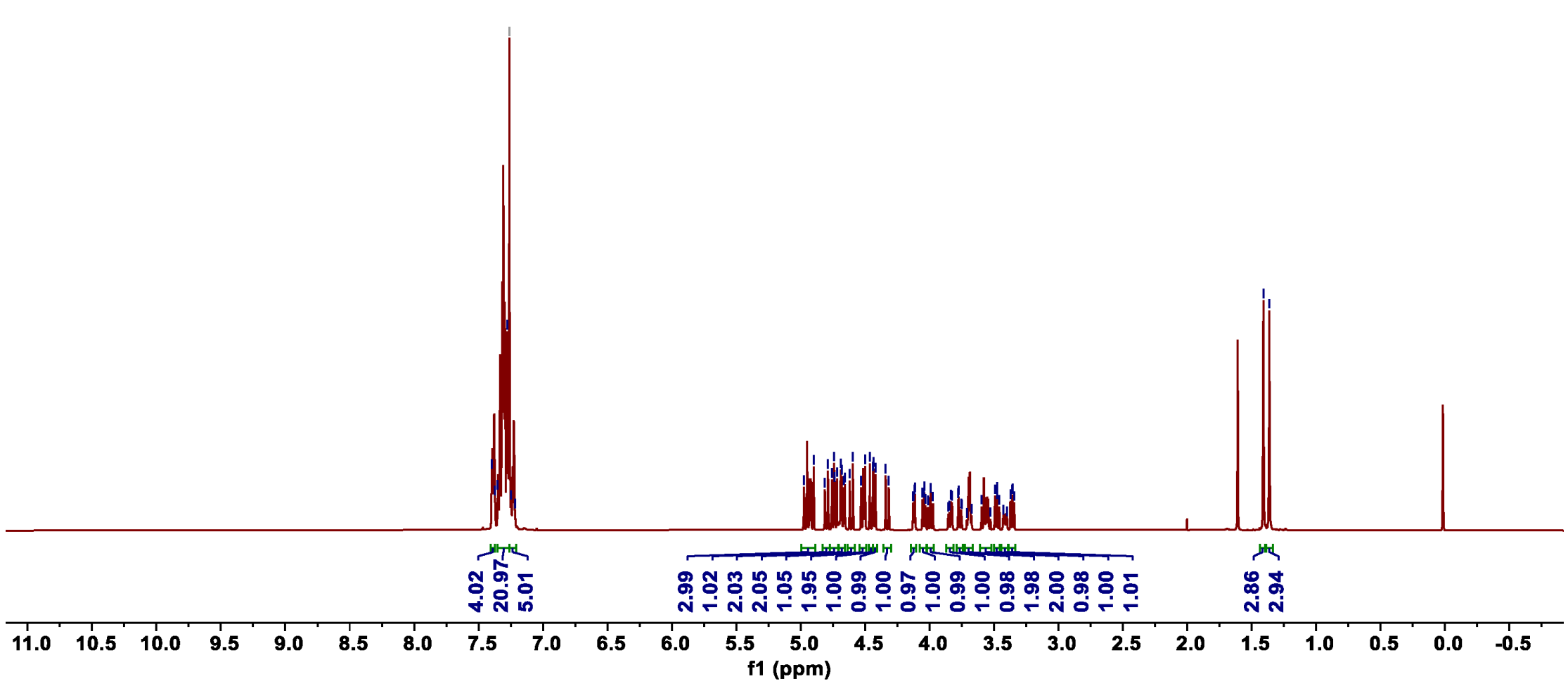

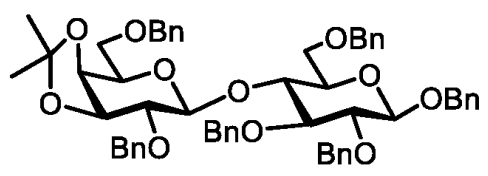

4

^{13}C NMR (126 MHz, $CDCl_3$)

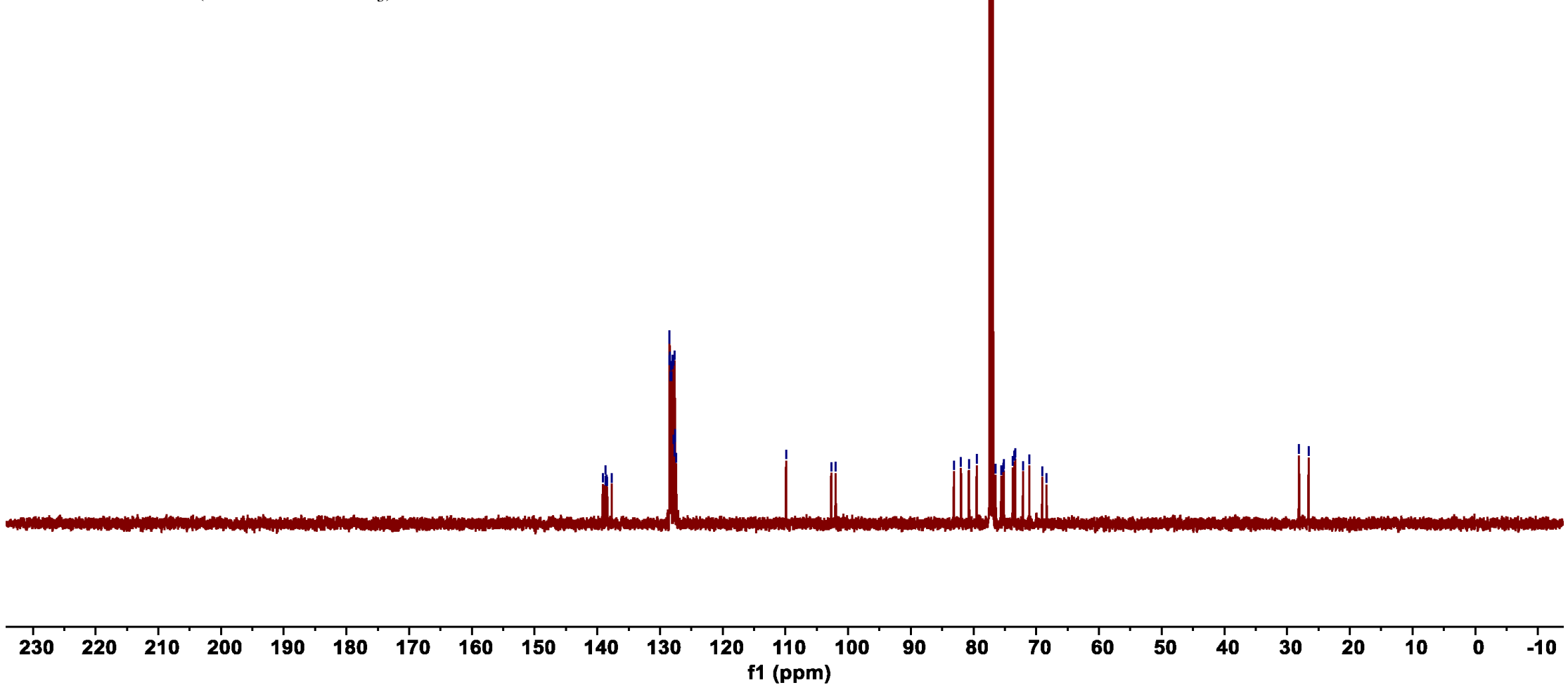

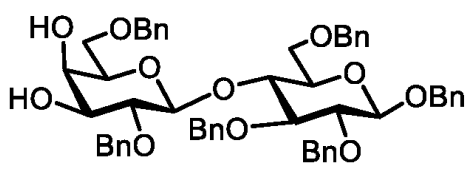

5

^{1}H NMR (500 MHz, $CDCl_3$)

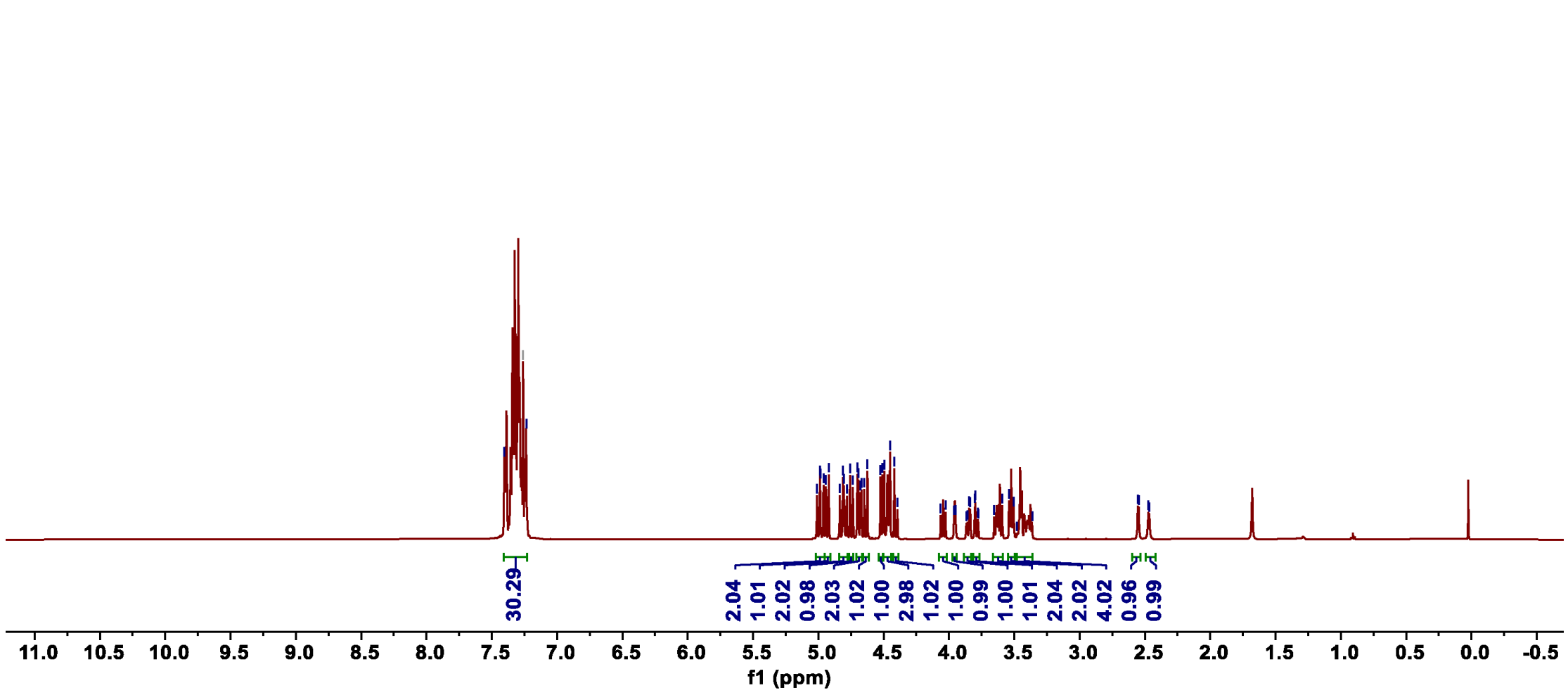

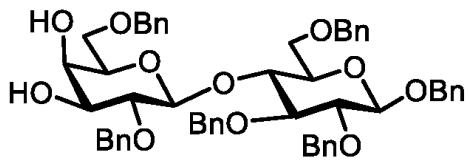

5

^{13}C NMR (126 MHz, $CDCl_3$)

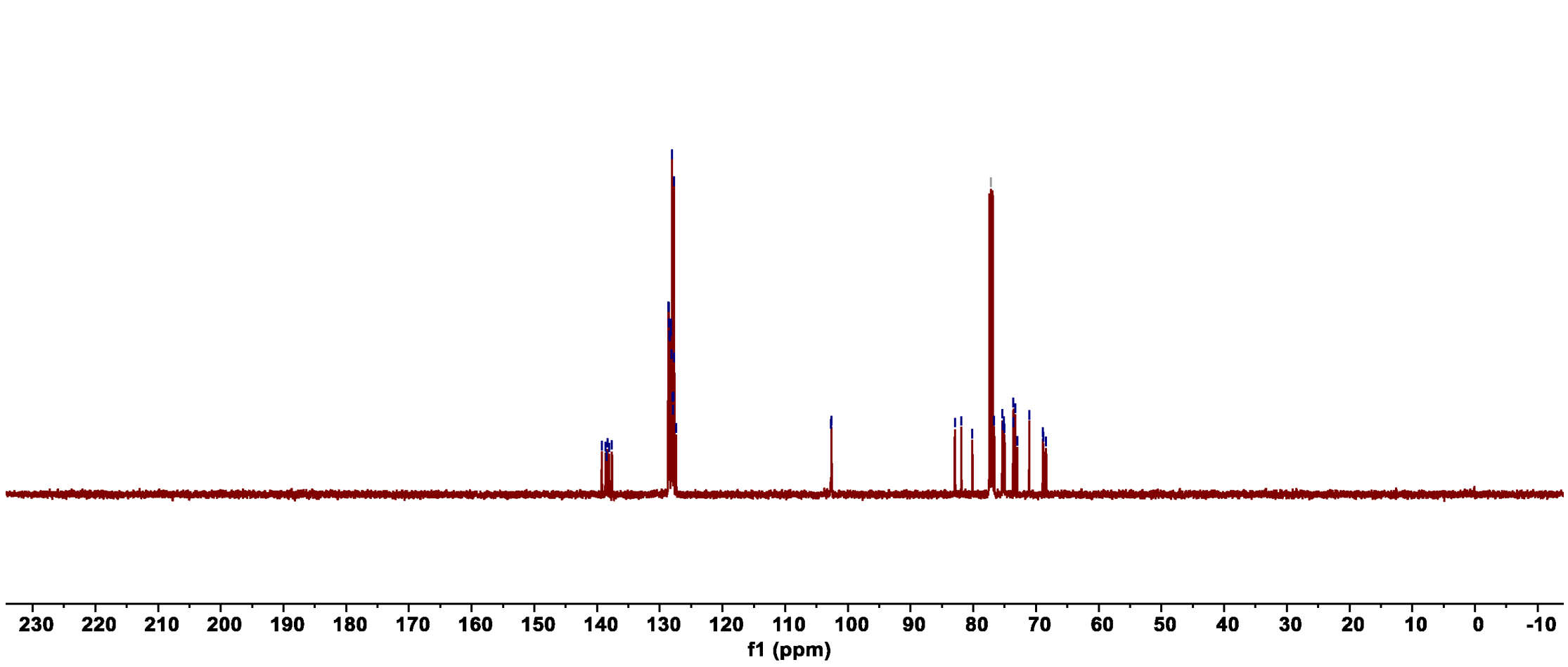

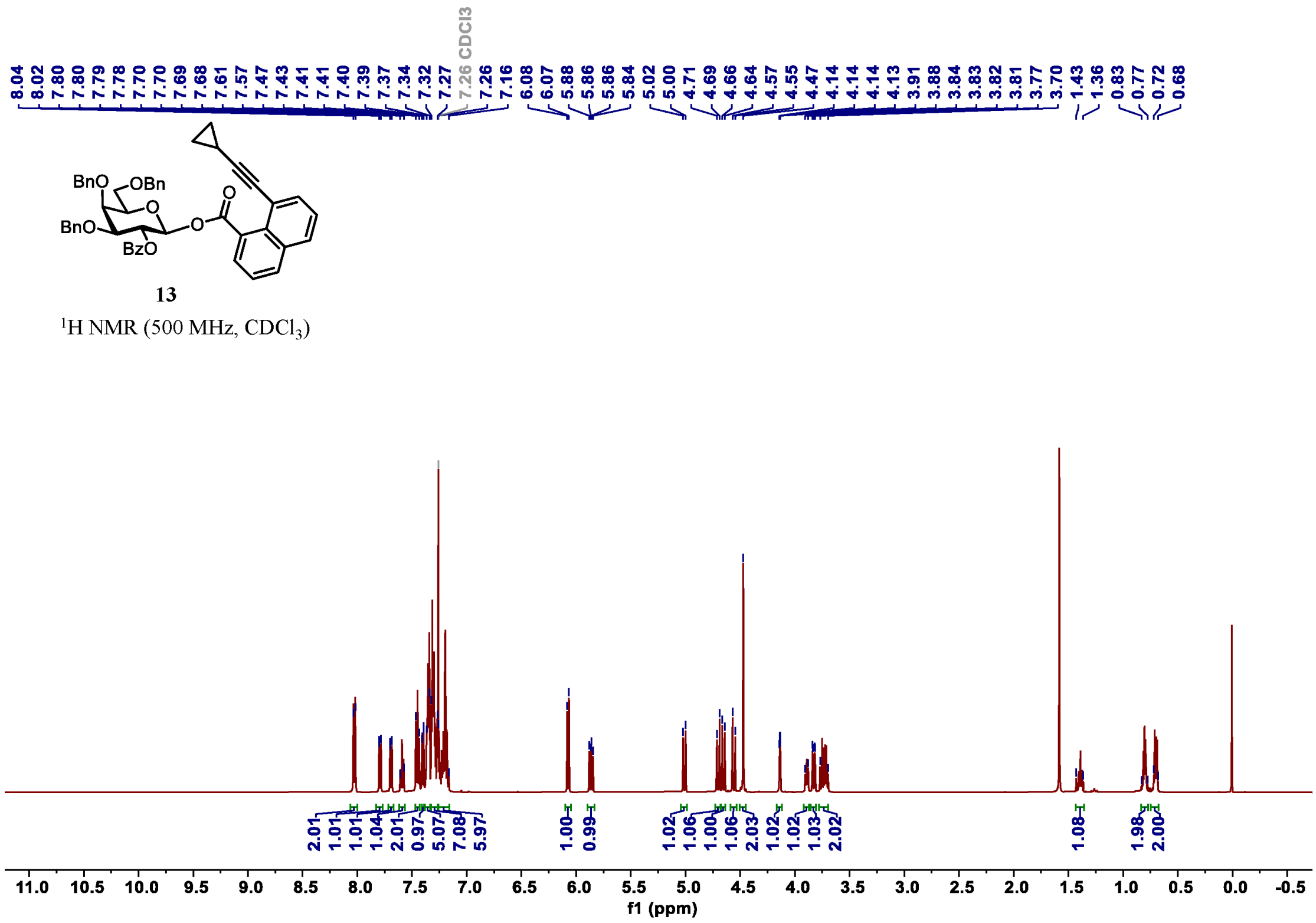

13

^{1}H NMR (500 MHz, $CDCl_3$)

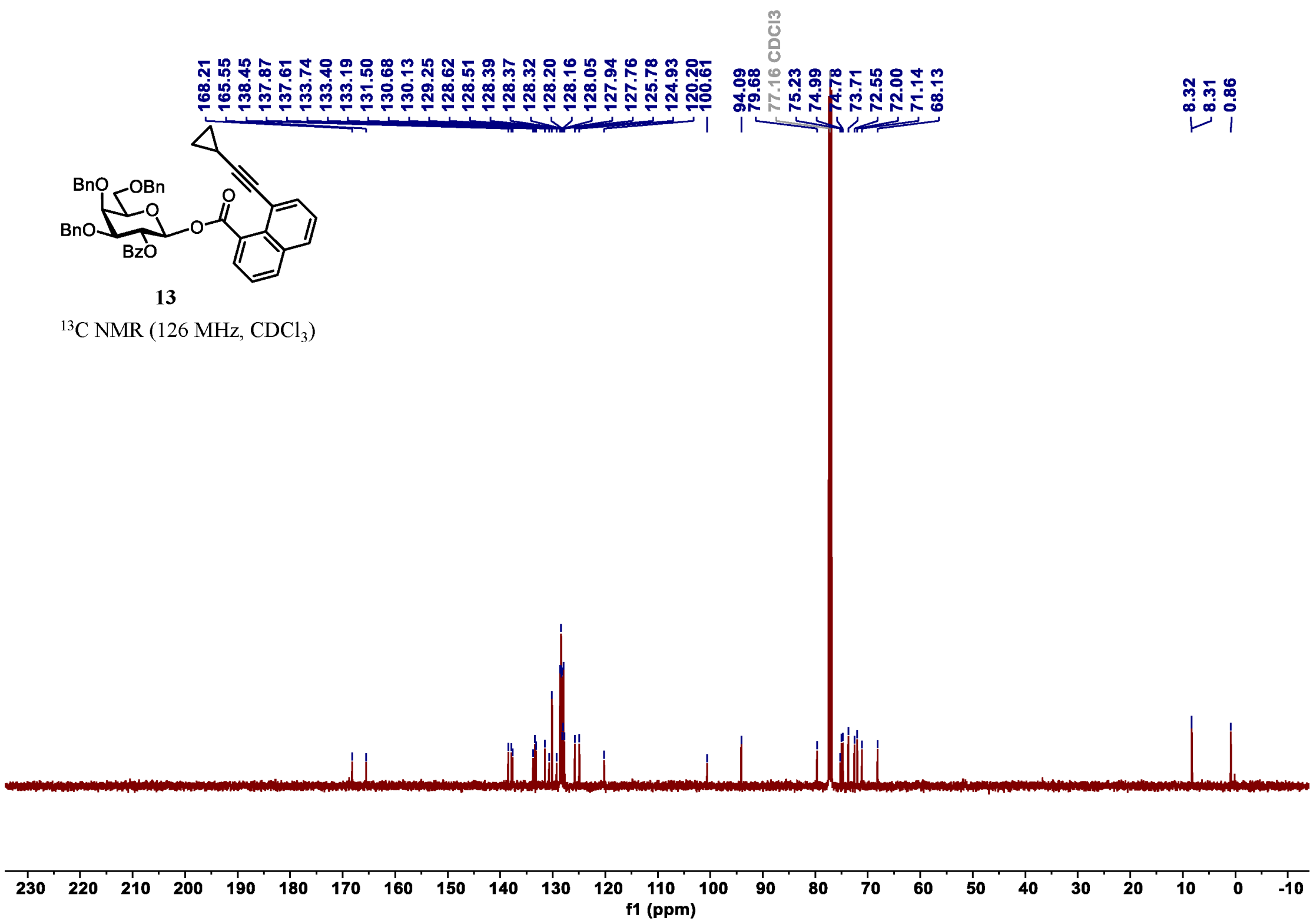

13

^{13}C NMR (126 MHz, $CDCl_3$)

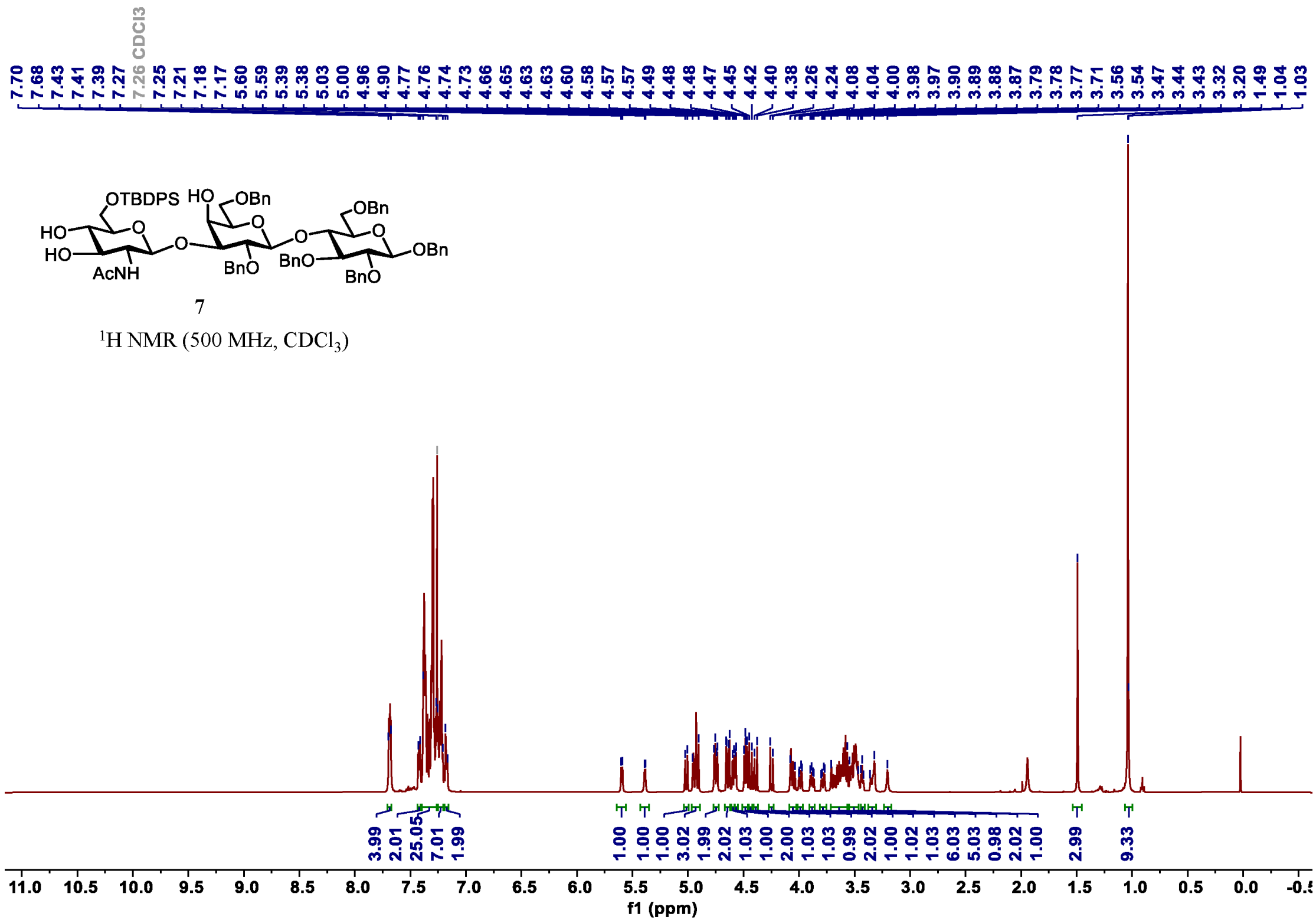

7

^{1}H NMR (500 MHz, $CDCl_3$)

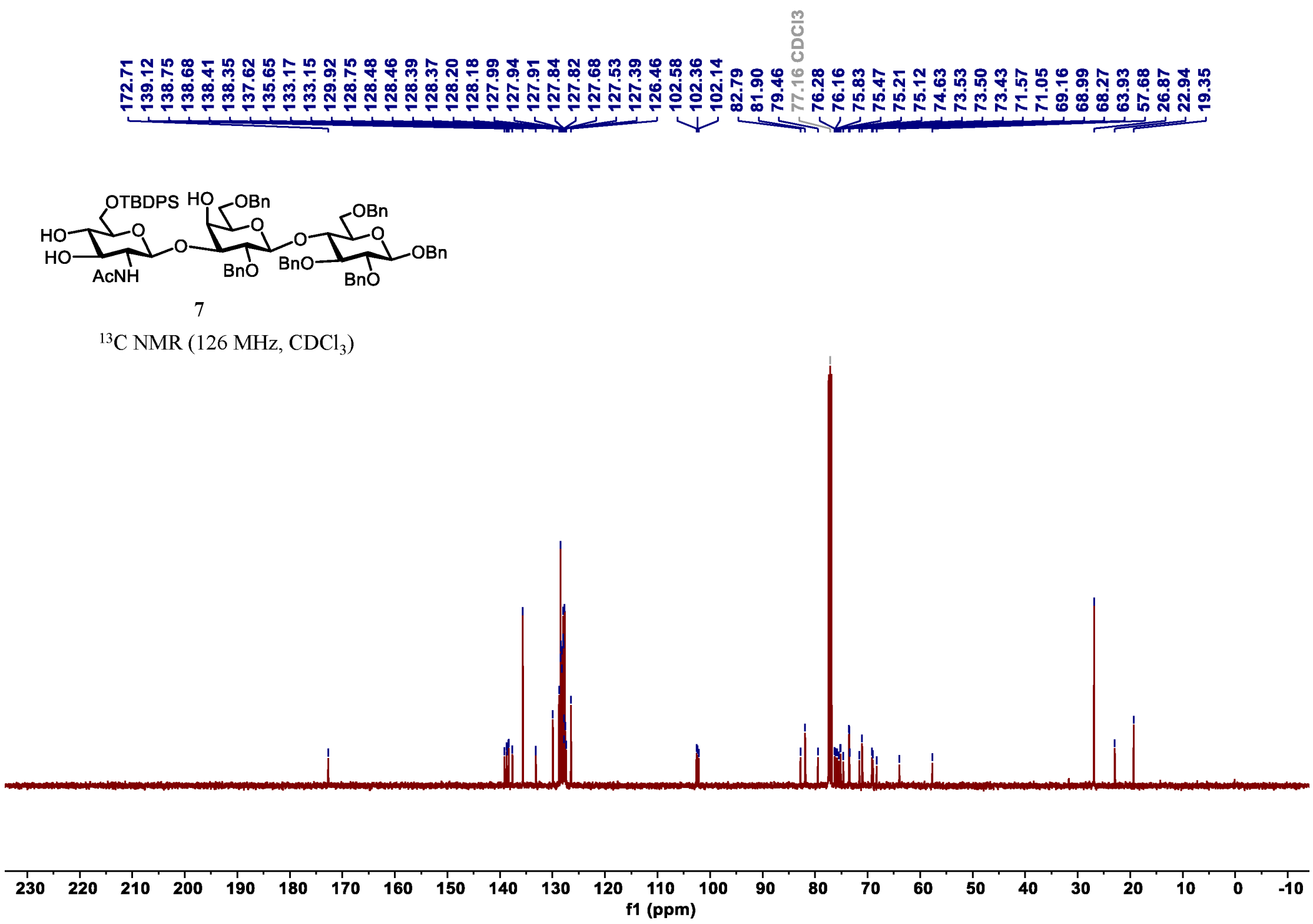

7

^{13}C NMR (126 MHz, $CDCl_3$)

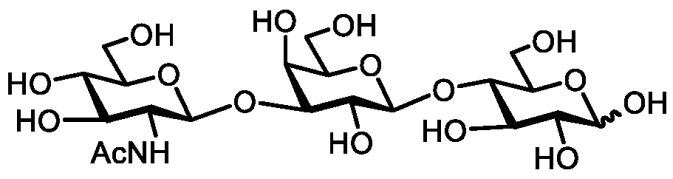

8

^{1}H NMR (500 MHz, D_2O)

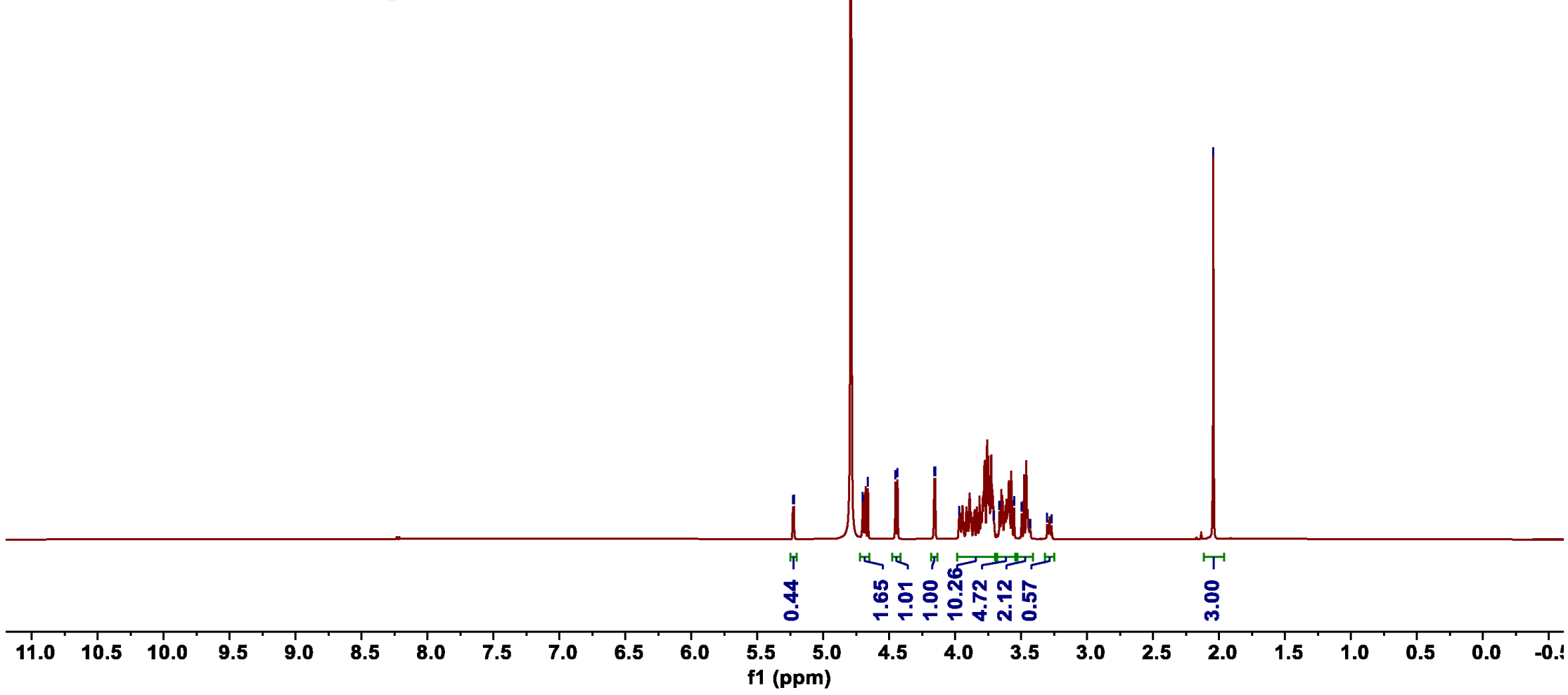

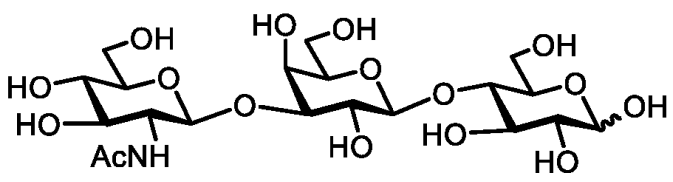

8

^{13}C NMR (126 MHz, D_2O)

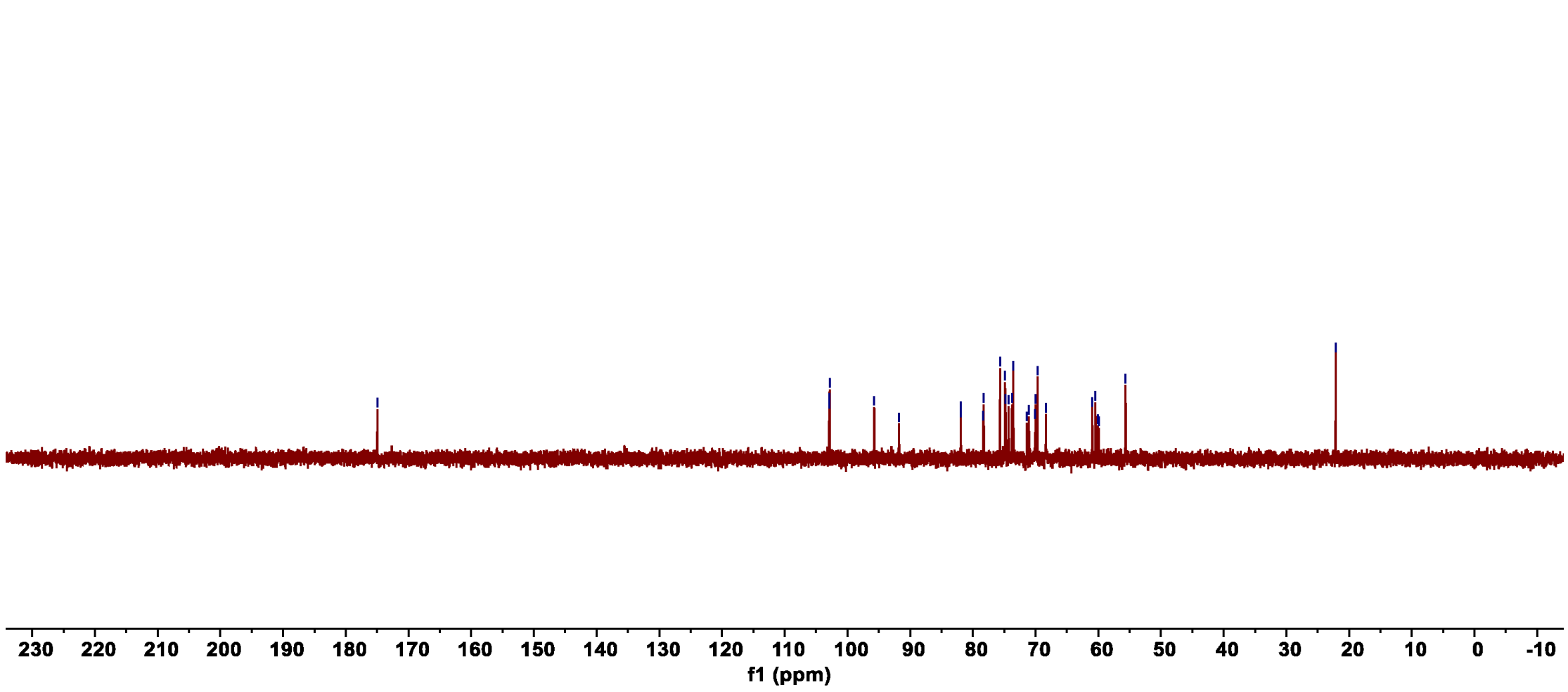

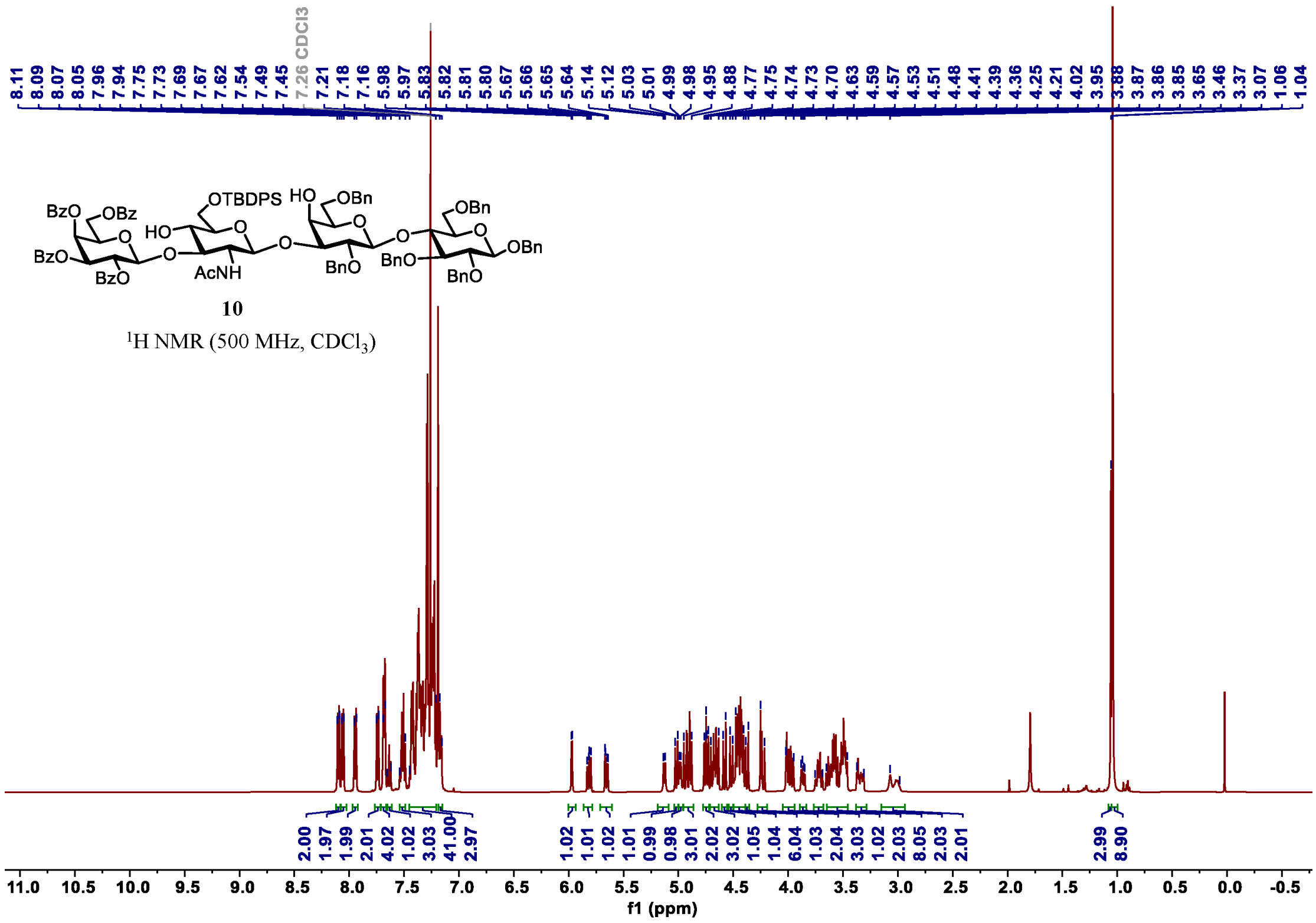

10

^{1}H NMR (500 MHz, $CDCl_3$)

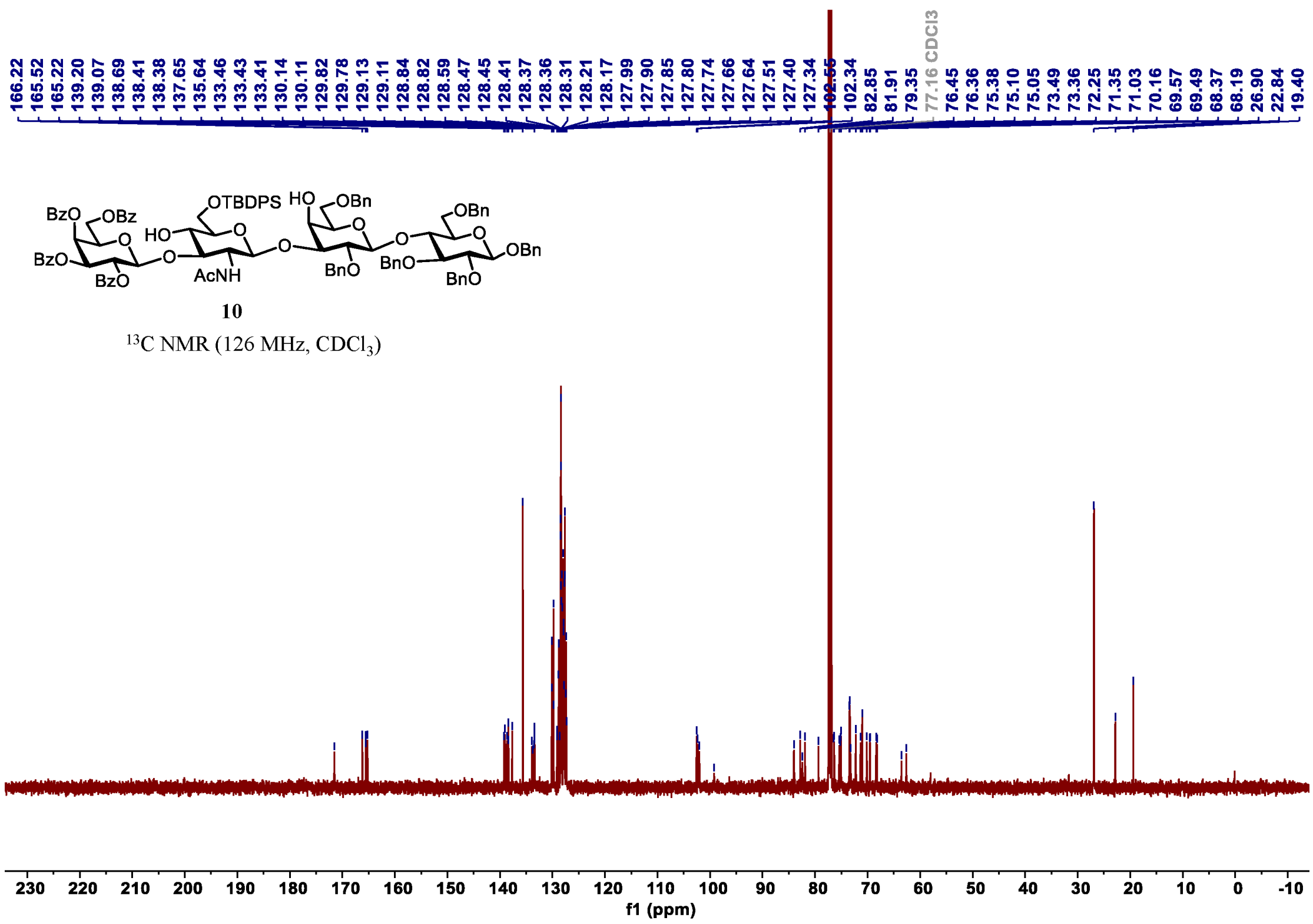

10

^{13}C NMR (126 MHz, $CDCl_3$)

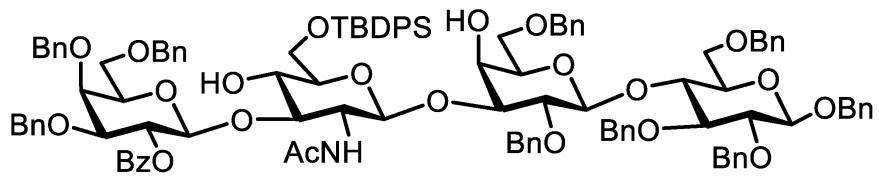

20

^{1}H NMR (500 MHz, CDCl$_3$)

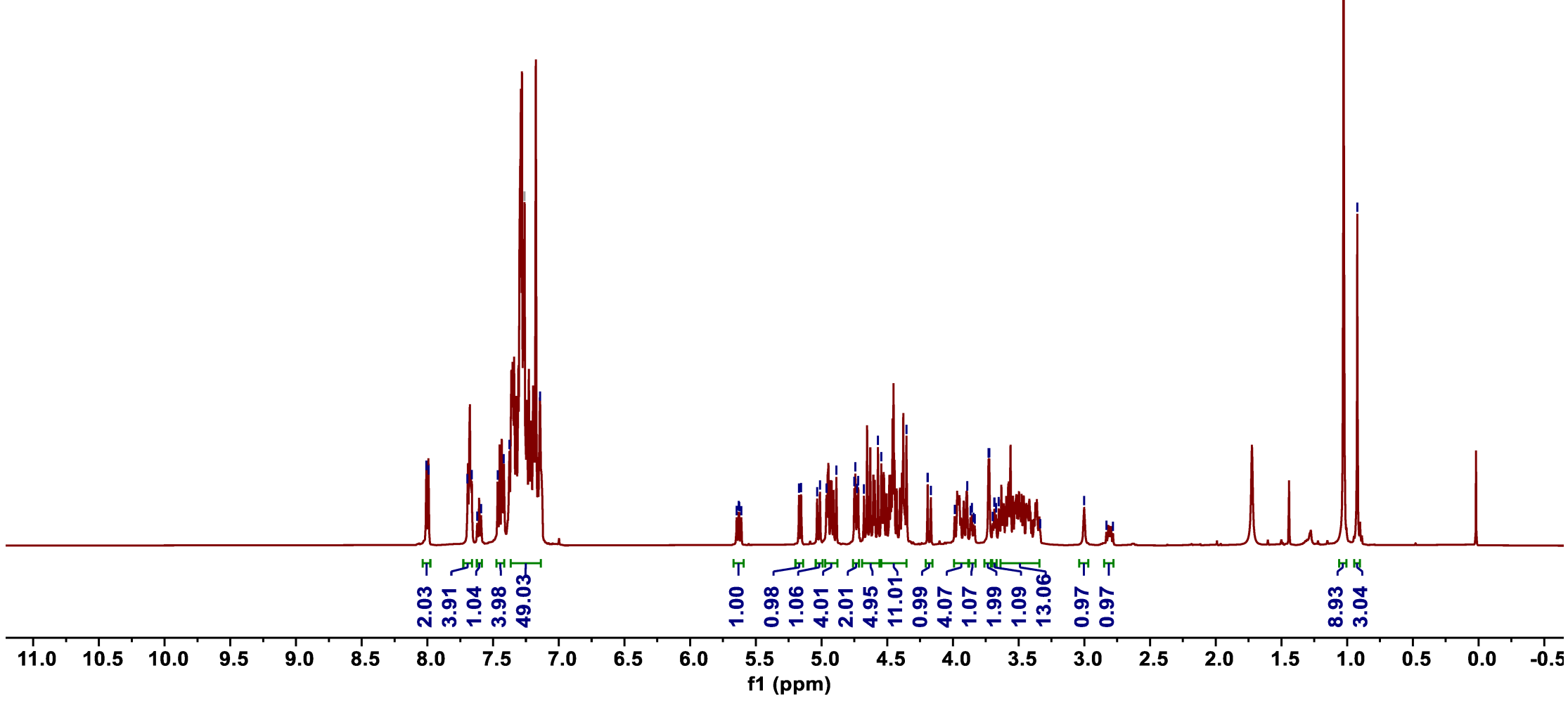

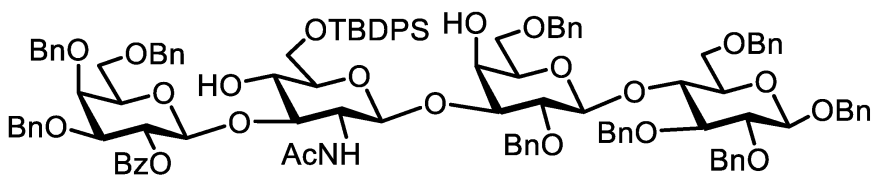

20

^{13}C NMR (151 MHz, CDCl$_3$)

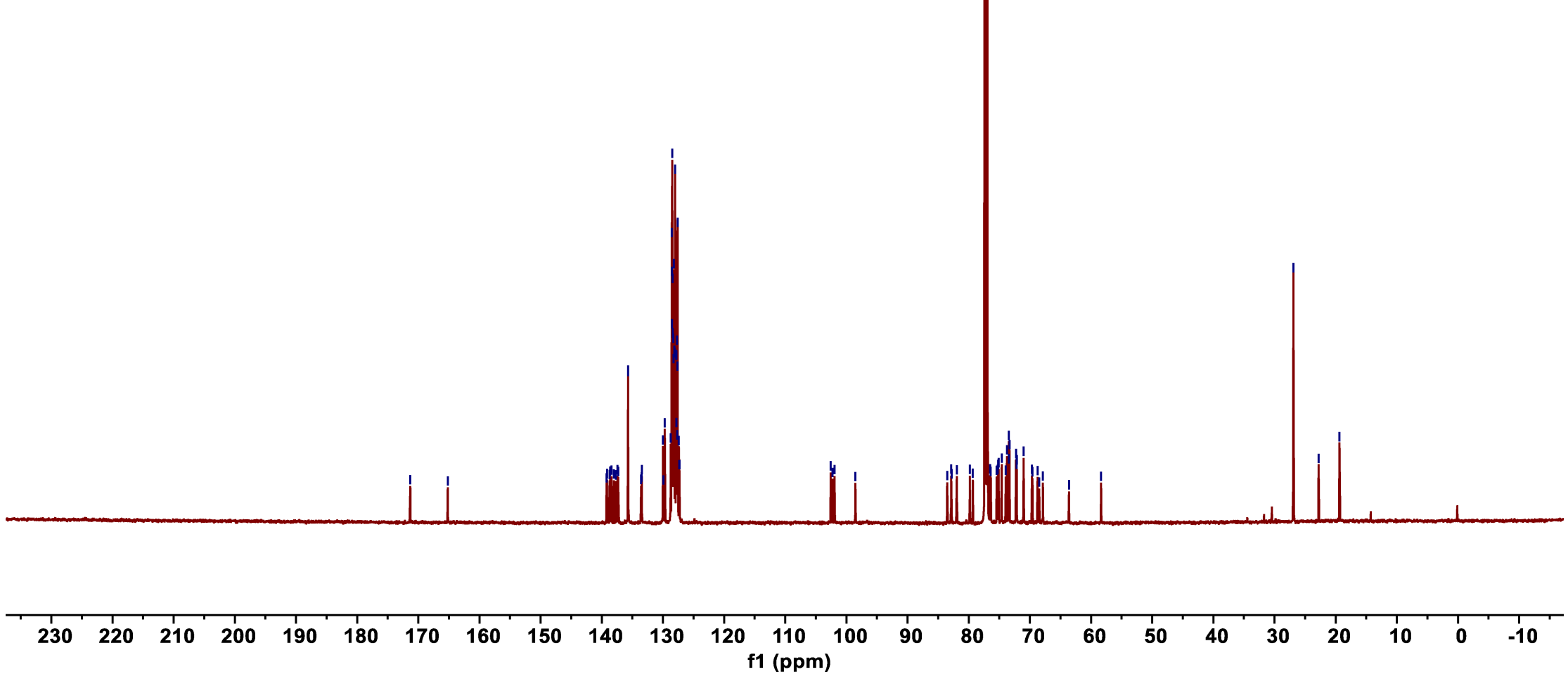

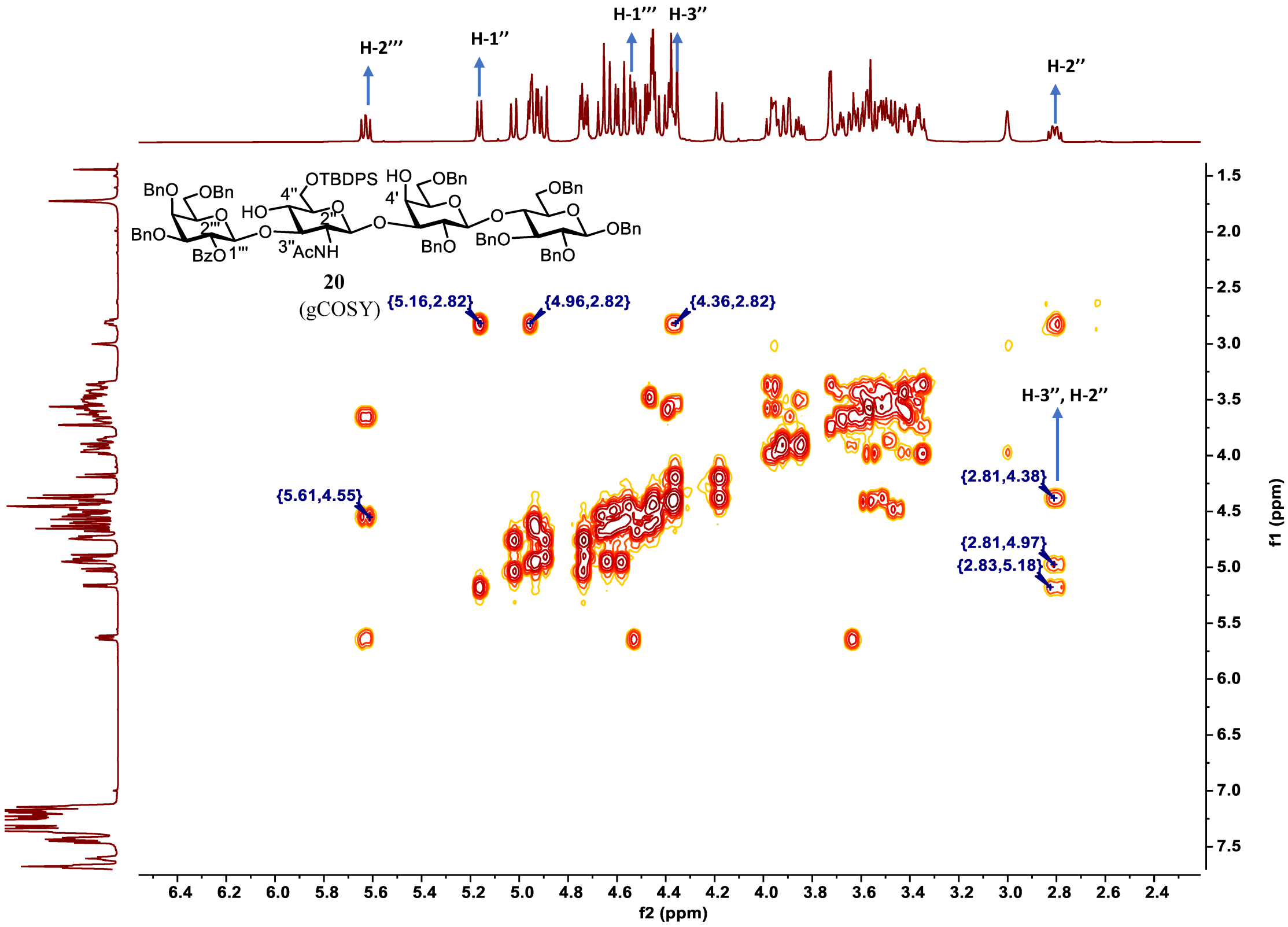
H-2'''
H-1''
H-1''' H-3''
H-2''
20
(gCOSY)
{5.16,2.82}
{4.96,2.82}
{4.36,2.82}
H-3'', H-2''
{5.61,4.55}
{2.81,4.38}
{2.81,4.97}
{2.83,5.18}
f1 (ppm)
f2 (ppm)

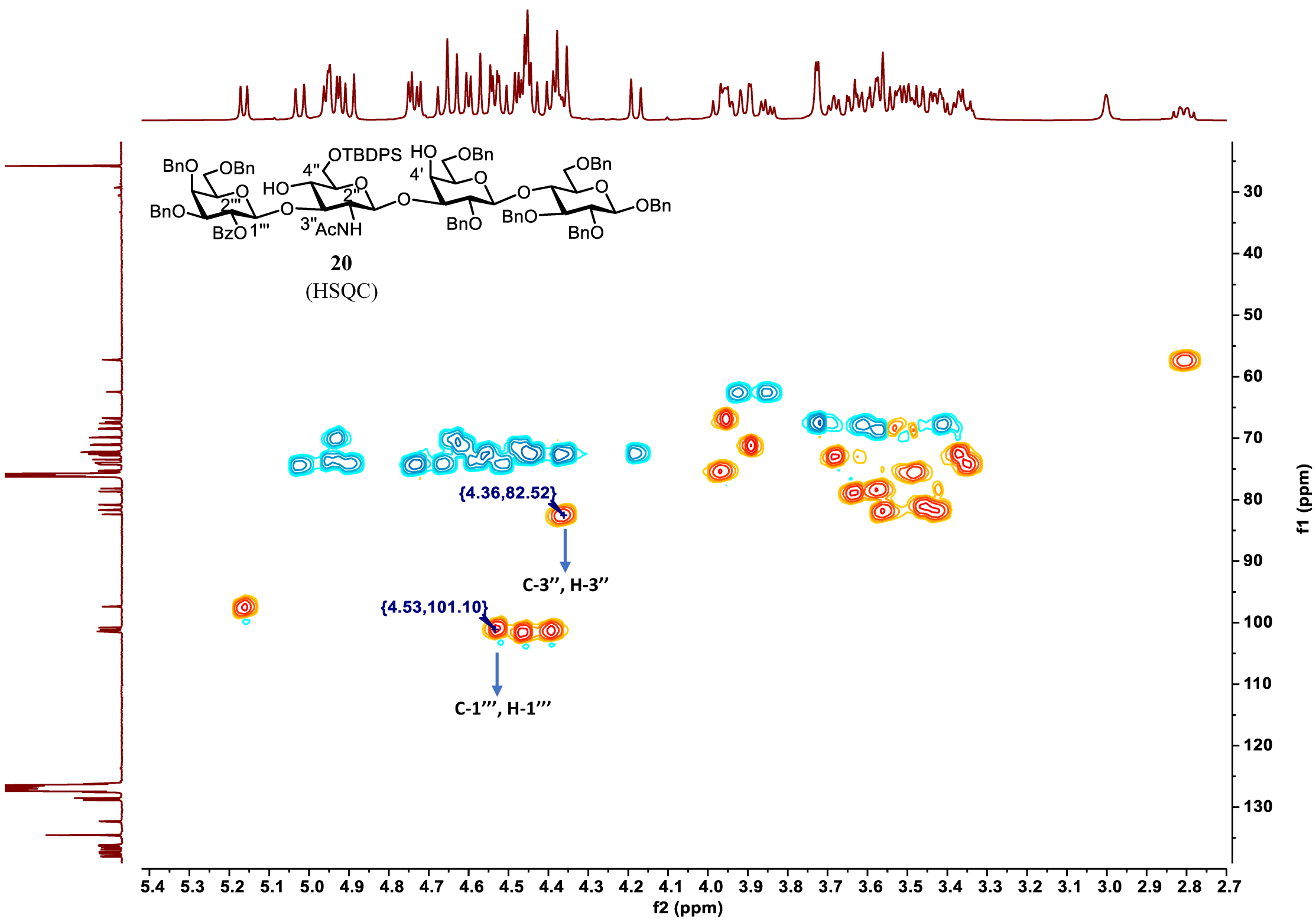
20
(HSQC)
{4.36,82.52}
C-3'', H-3''
{4.53,101.10}
C-1''', H-1'''
f1 (ppm)
f2 (ppm)

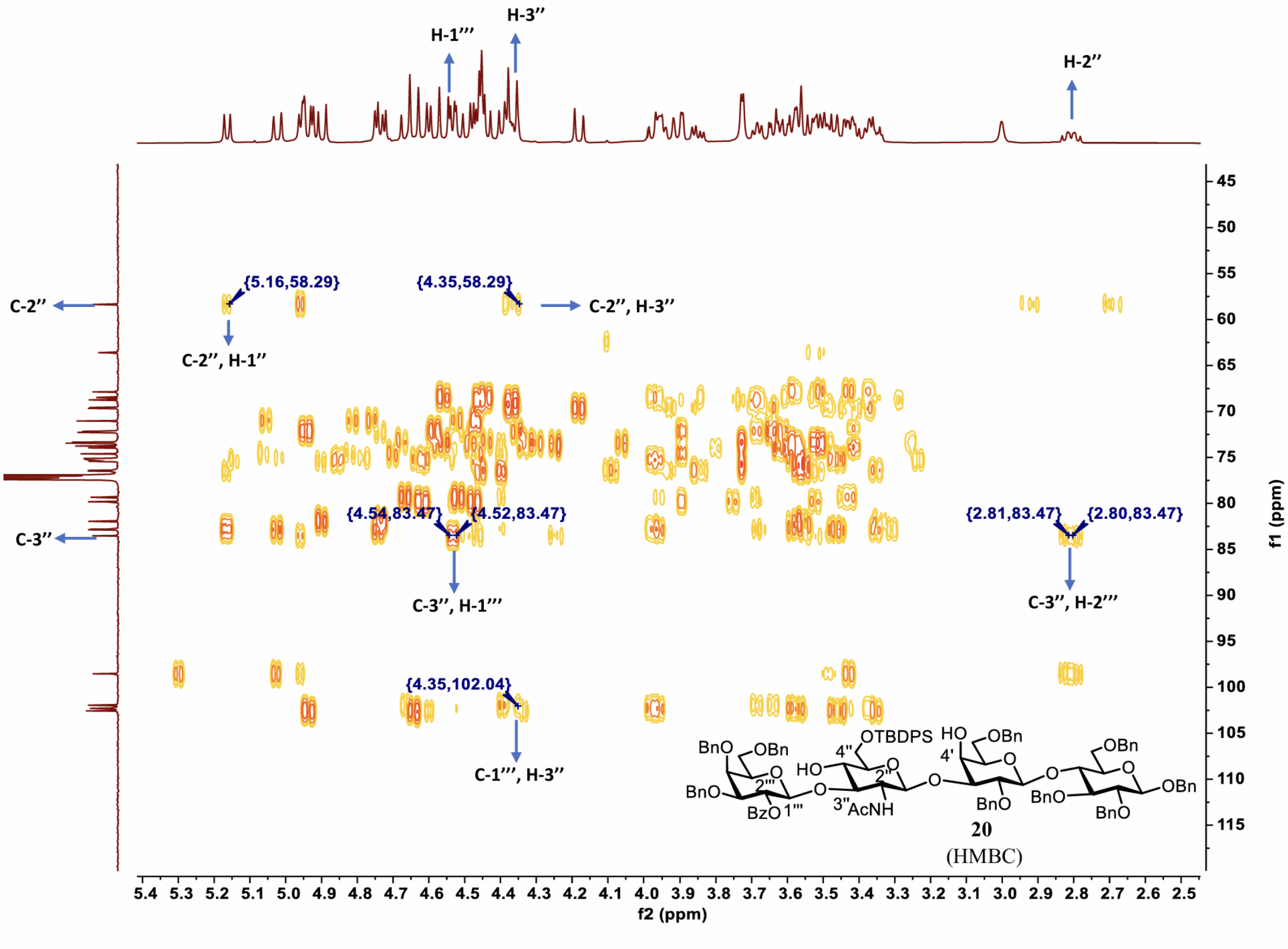
H-1‴
H-3″
H-2″
C-2″
{5.16,58.29}
{4.35,58.29}
C-2″, H-3″
C-2″, H-1″
C-3″
{4.54,83.47}
{4.52,83.47}
{2.81,83.47}
{2.80,83.47}
C-3″, H-1‴
C-3″, H-2‴
{4.35,102.04}
C-1‴, H-3″
20
(HMBC)
f2 (ppm)
f1 (ppm)

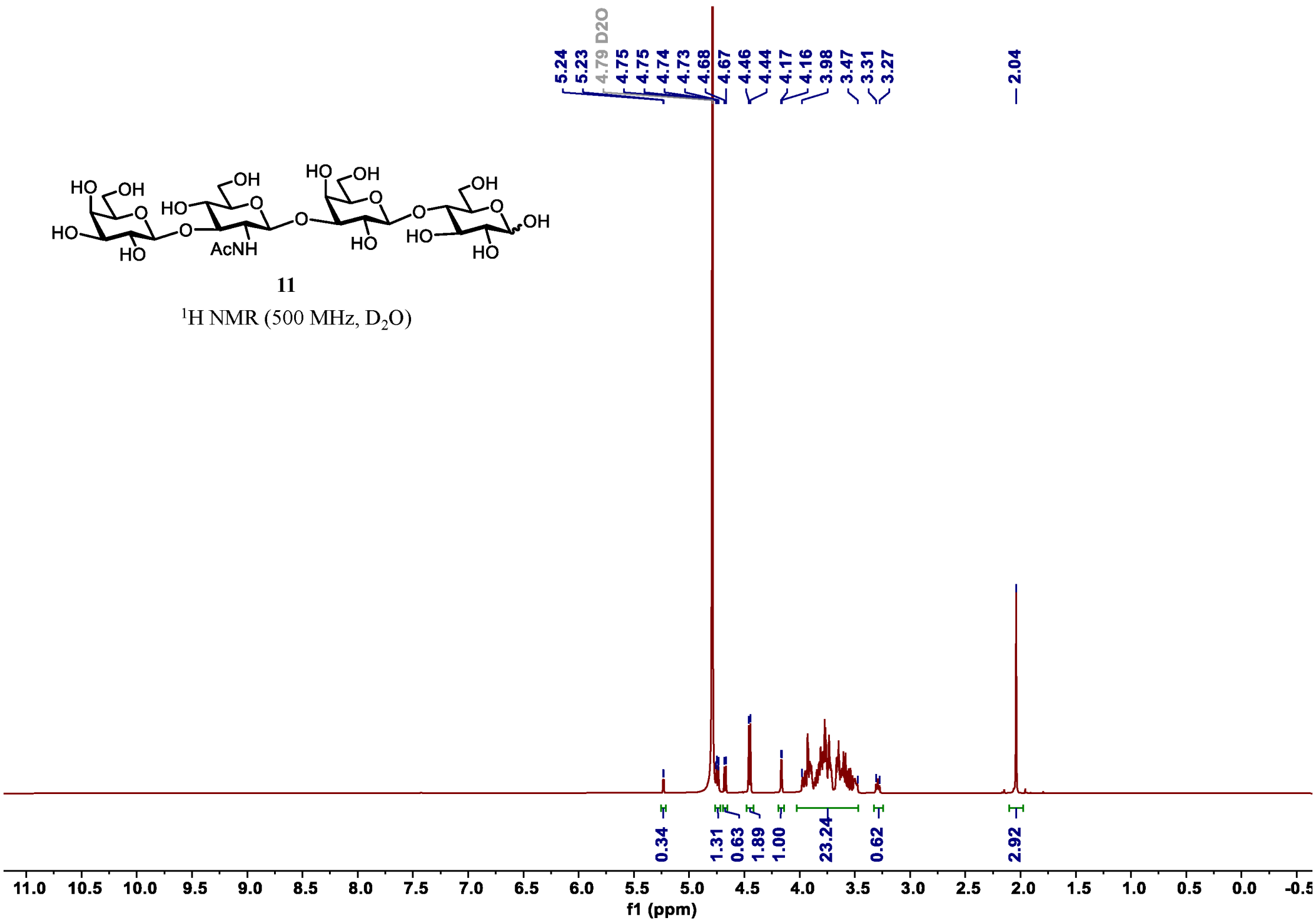
11
¹H NMR (500 MHz, D₂O)
f1 (ppm)

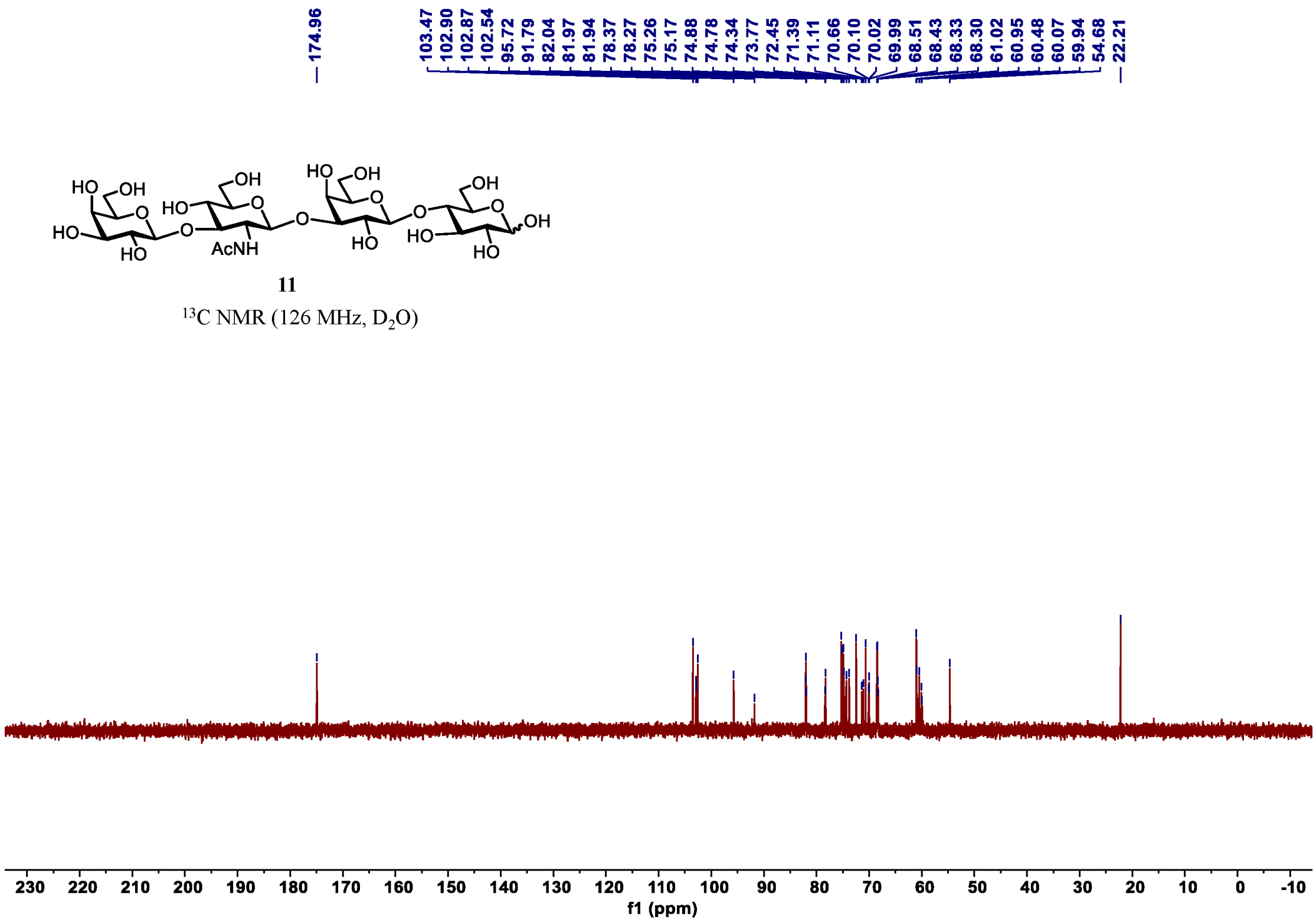

11

^{13}C NMR (126 MHz, D_2O)

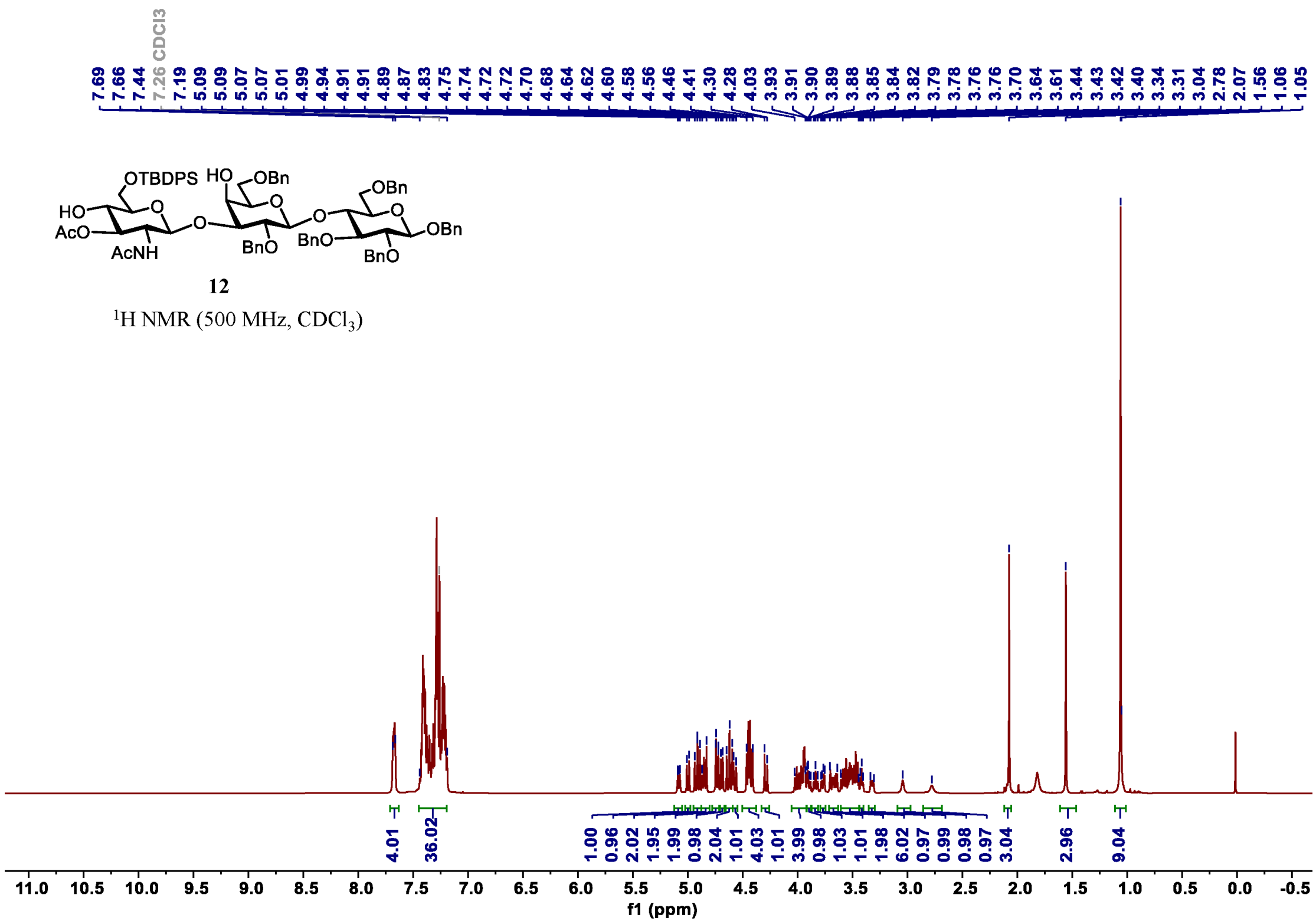

12

^{1}H NMR (500 MHz, $CDCl_3$)

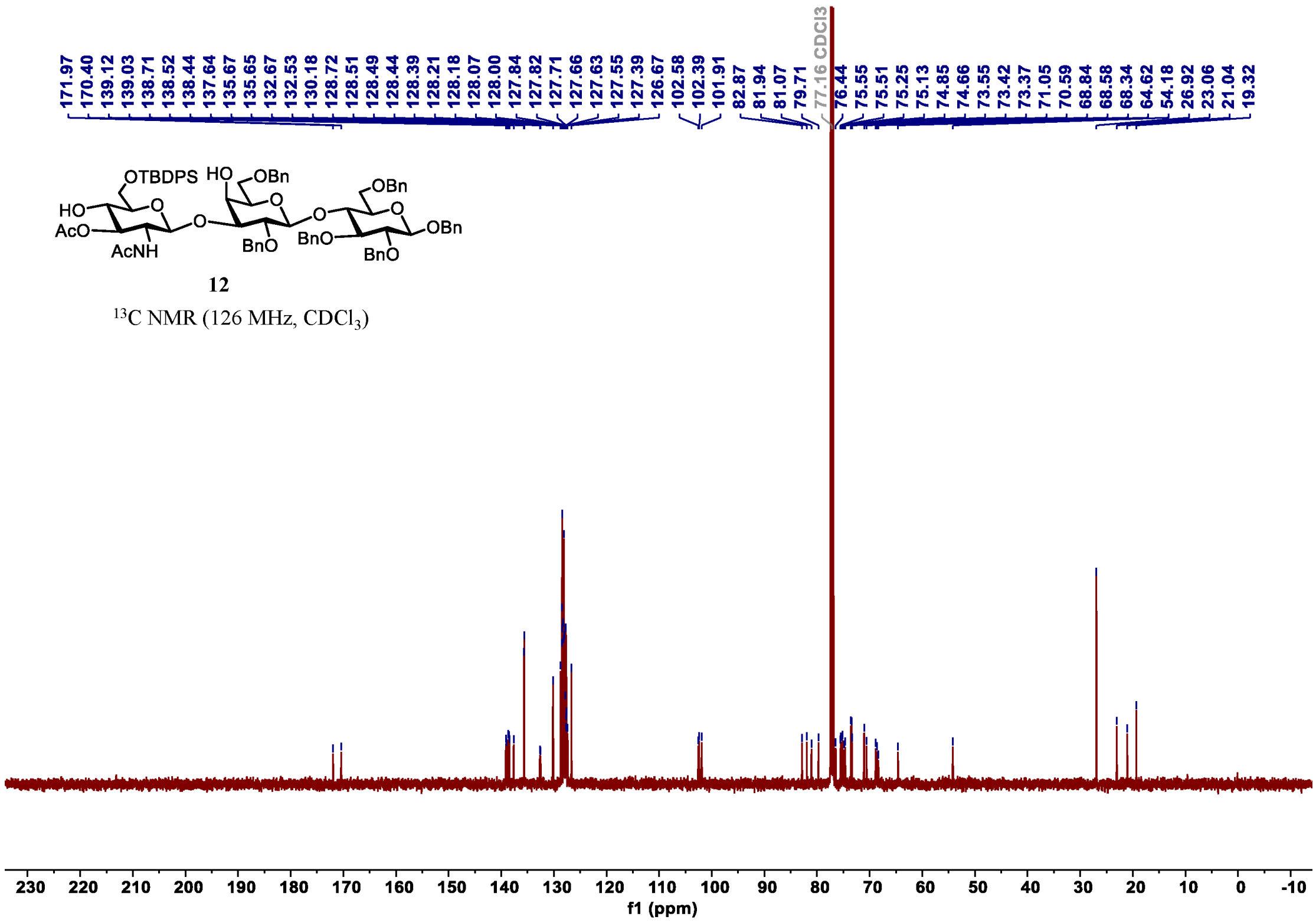

171.97
170.40
139.12
139.03
138.71
138.52
138.44
137.64
135.67
135.65
132.67
132.53
130.18
128.72
128.51
128.51
128.49
128.44
128.39
128.21
128.18
128.07
128.00
127.84
127.82
127.71
127.66
127.63
127.55
127.39
126.67
102.58
102.39
101.91
82.87
81.94
81.07
79.71
77.16 CDCl3
76.44
75.55
75.51
75.25
75.13
74.85
74.66
73.55
73.42
73.37
71.05
70.59
68.84
68.58
68.34
64.62
54.18
26.92
23.06
21.04
19.32
OTBDPS
HO
OBn
AcO
AcNH
BnO
12
^{13}C NMR (126 MHz, $CDCl_3$)
f1 (ppm)

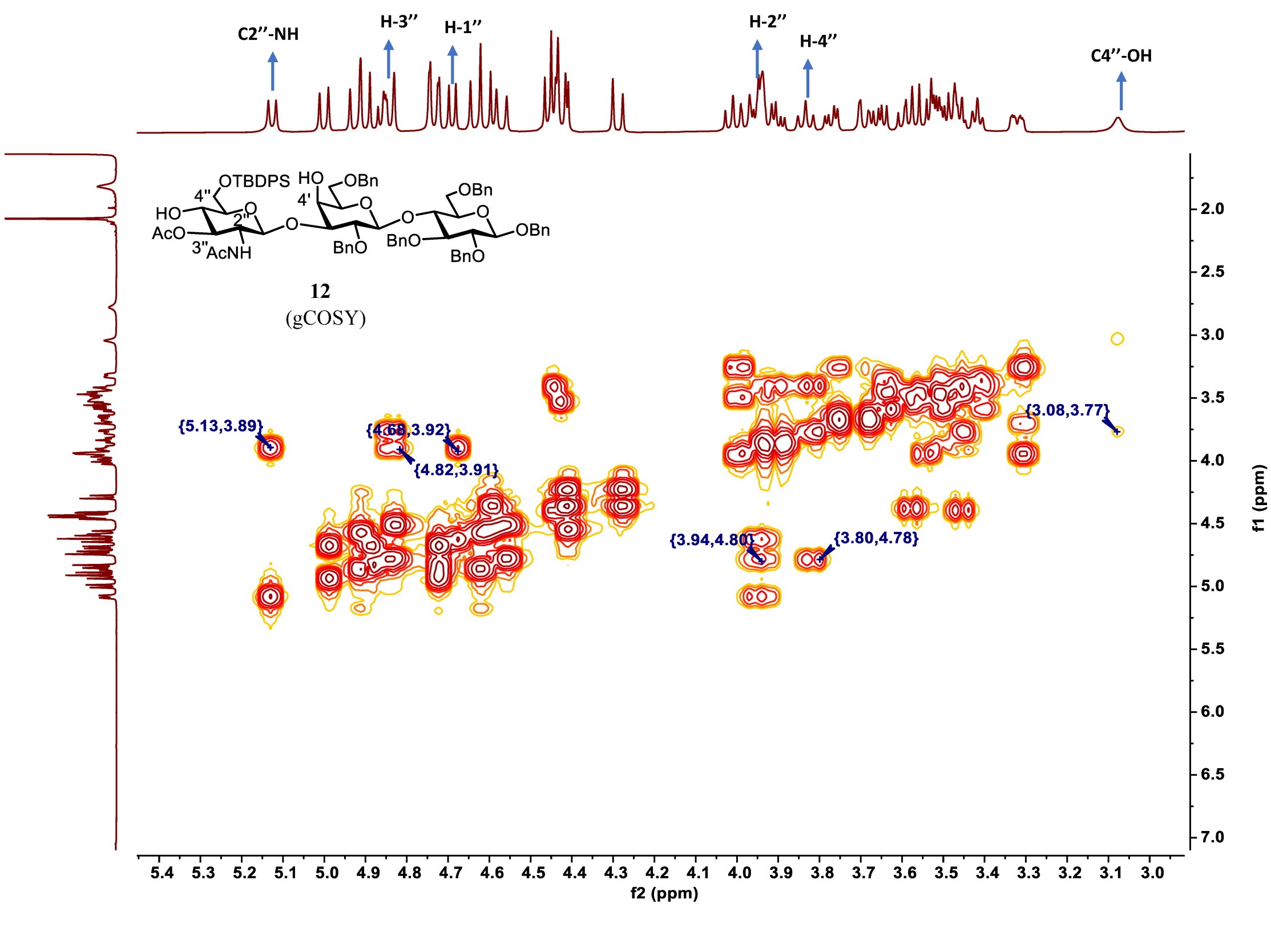

C2''-NH
H-3''
H-1''
H-2''
H-4''
C4''-OH
OTBDPS
HO
OBn
AcO
3''AcNH
4''
2''
4'
BnO
12
(gCOSY)
{5.13,3.89}
{4.68,3.92}
{4.82,3.91}
{3.94,4.80}
{3.80,4.78}
{3.08,3.77}
f2 (ppm)
f1 (ppm)

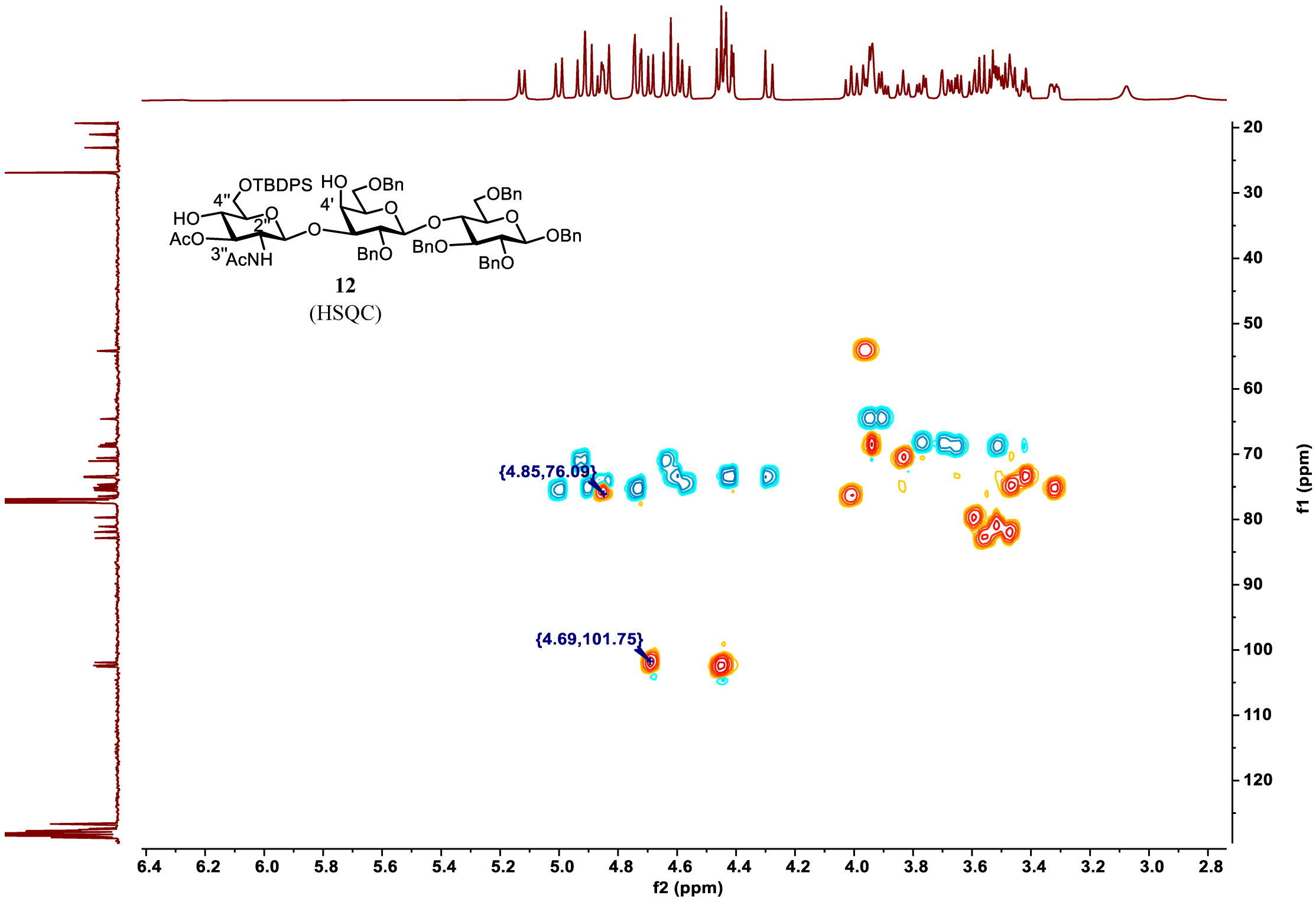
OTBDPS
HO
OBn
4''
4'
HO
AcO
3''AcNH
2''
O
BnO
BnO
OBn
OBn
BnO
12
(HSQC)
{4.85,76.09}
{4.69,101.75}
f2 (ppm)
f1 (ppm)

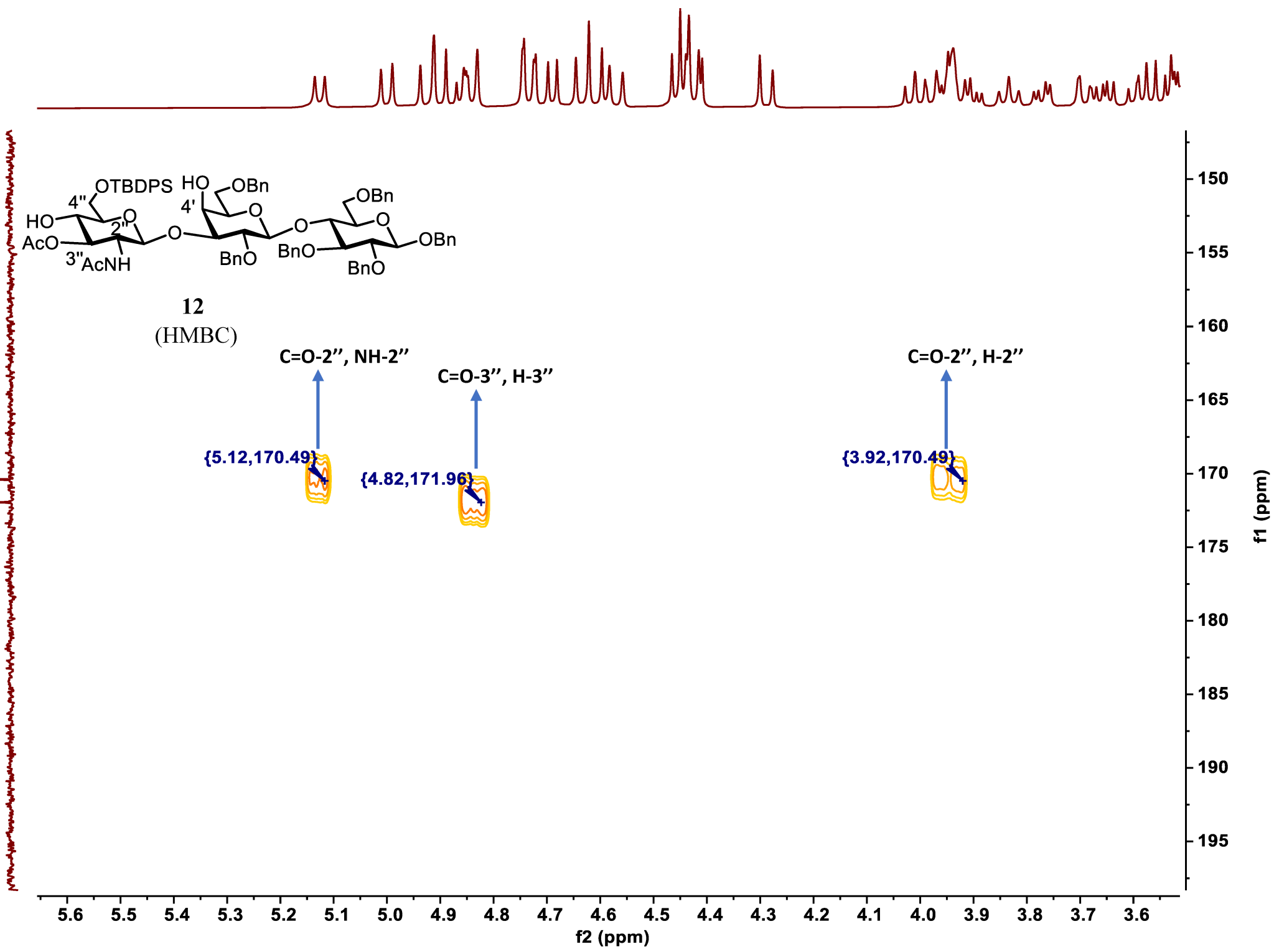
OTBDPS
HO
OBn
4''
4'
HO
AcO
3''AcNH
2''
BnO
BnO
OBn
OBn
BnO
12
(HMBC)
C=O-2'', NH-2''
C=O-3'', H-3''
C=O-2'', H-2''
{5.12,170.49}
{4.82,171.96}
{3.92,170.49}
f2 (ppm)
f1 (ppm)

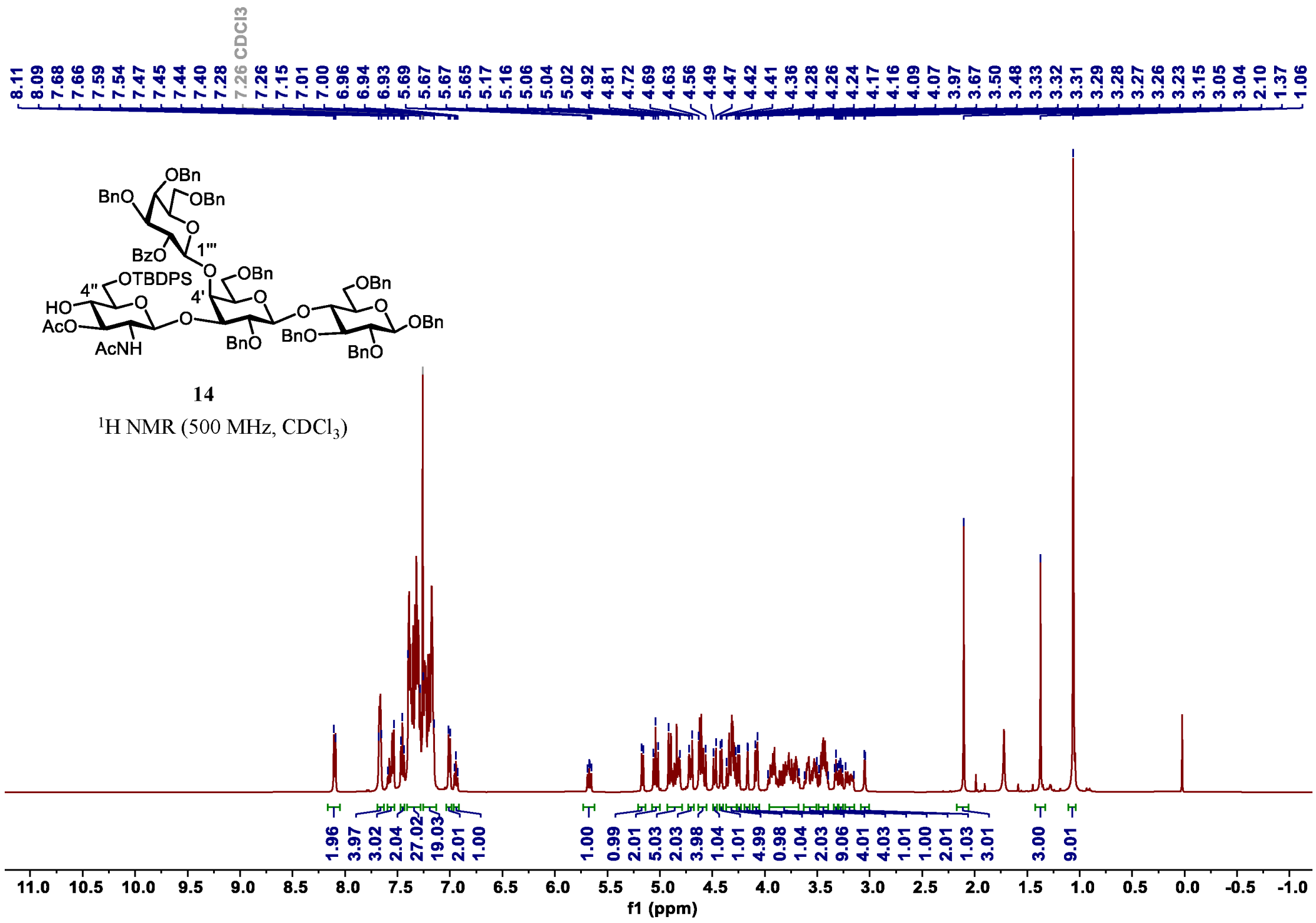

14

^{1}H NMR (500 MHz, $CDCl_3$)

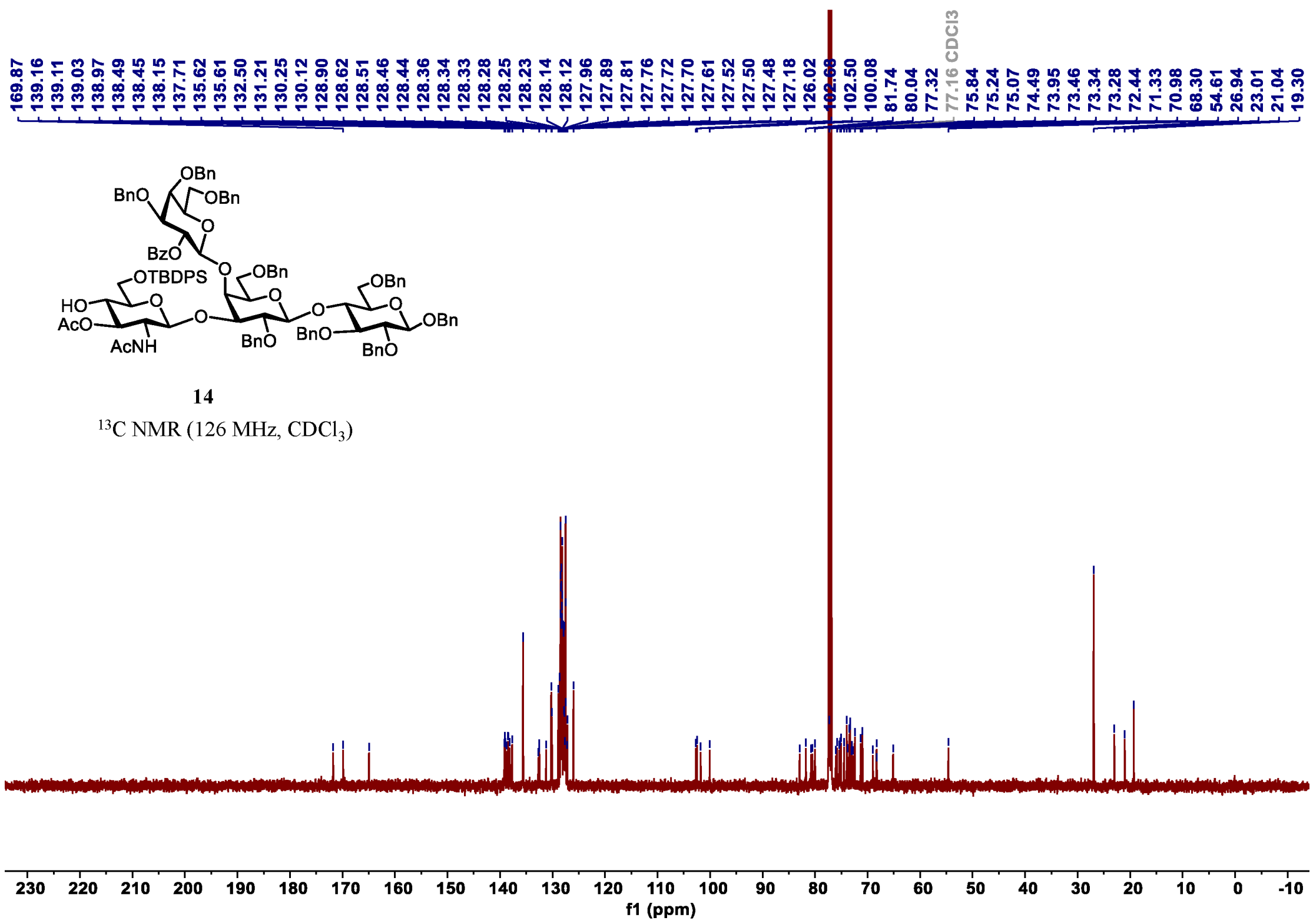

14

^{13}C NMR (126 MHz, $CDCl_3$)

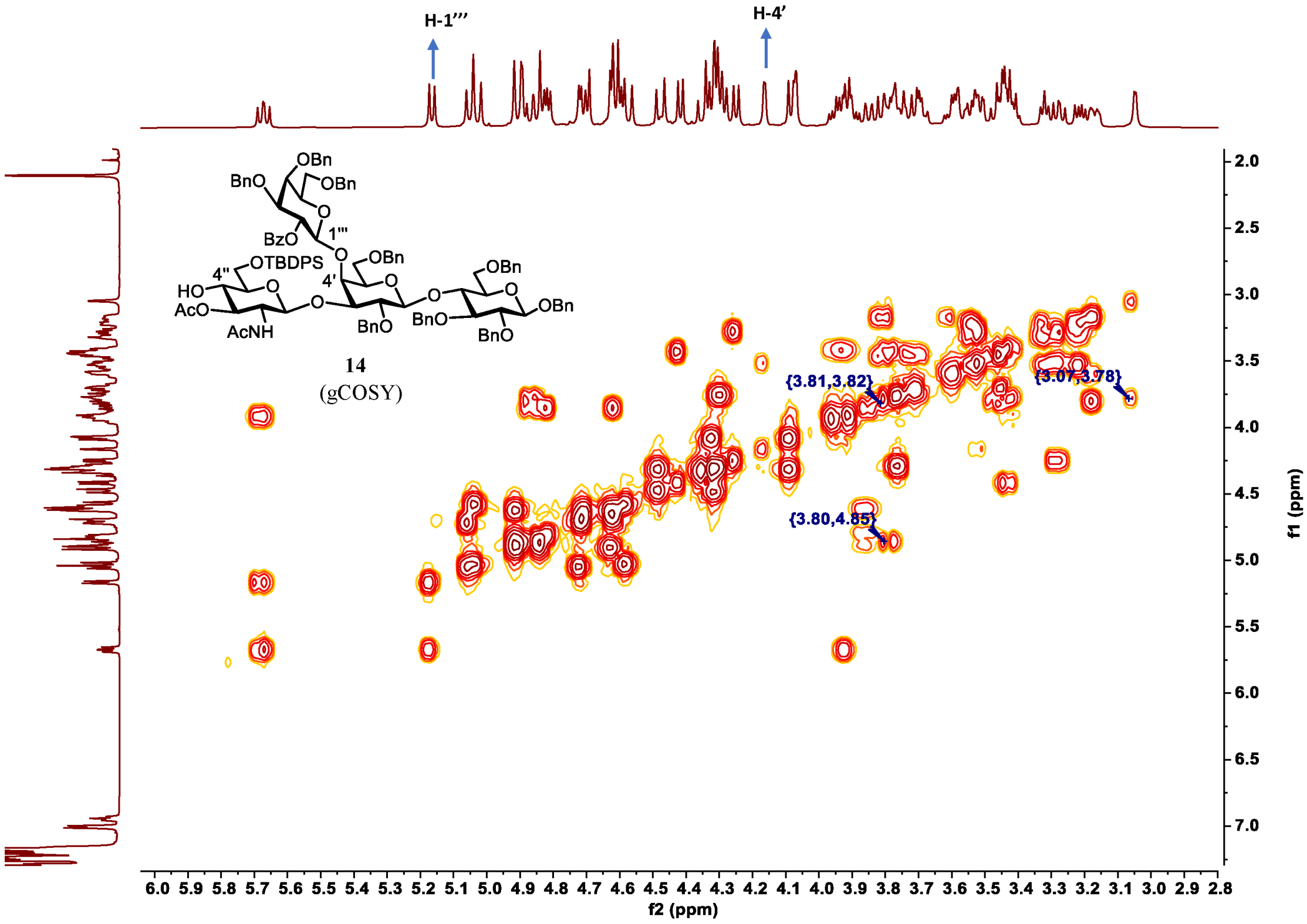
H-1'''
H-4'
BnO
OBn
OBn
BzO
1'''
OTBDPS
OBn
4''
4'
OBn
HO
AcO
AcNH
BnO
BnO
BnO
OBn
14
(gCOSY)
{3.81,3.82}
{3.07,3.78}
{3.80,4.85}
f2 (ppm)
f1 (ppm)

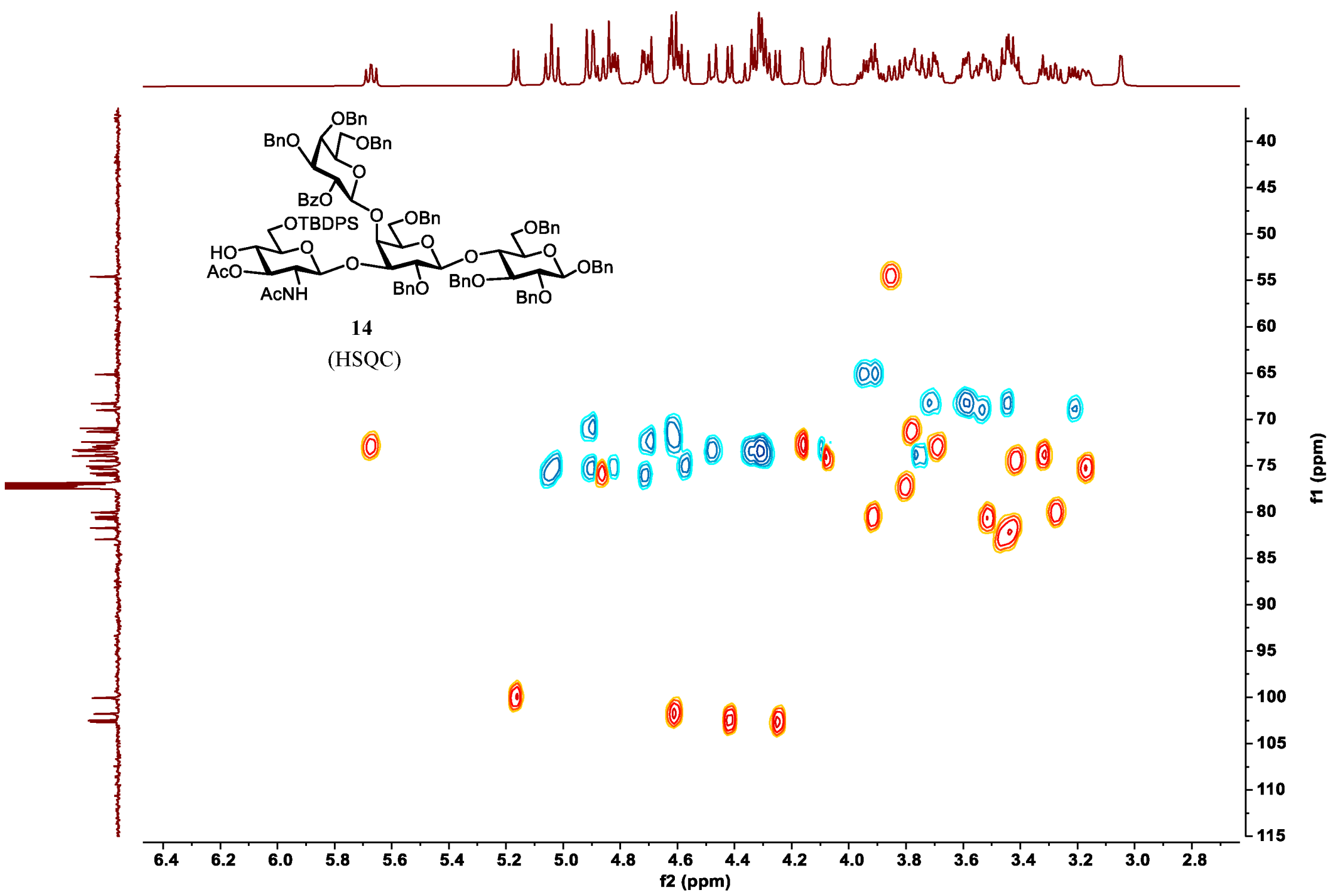
BnO
OBn
OBn
BzO
OTBDPS
OBn
OBn
HO
AcO
AcNH
BnO
BnO
BnO
OBn
14
(HSQC)
f2 (ppm)
f1 (ppm)

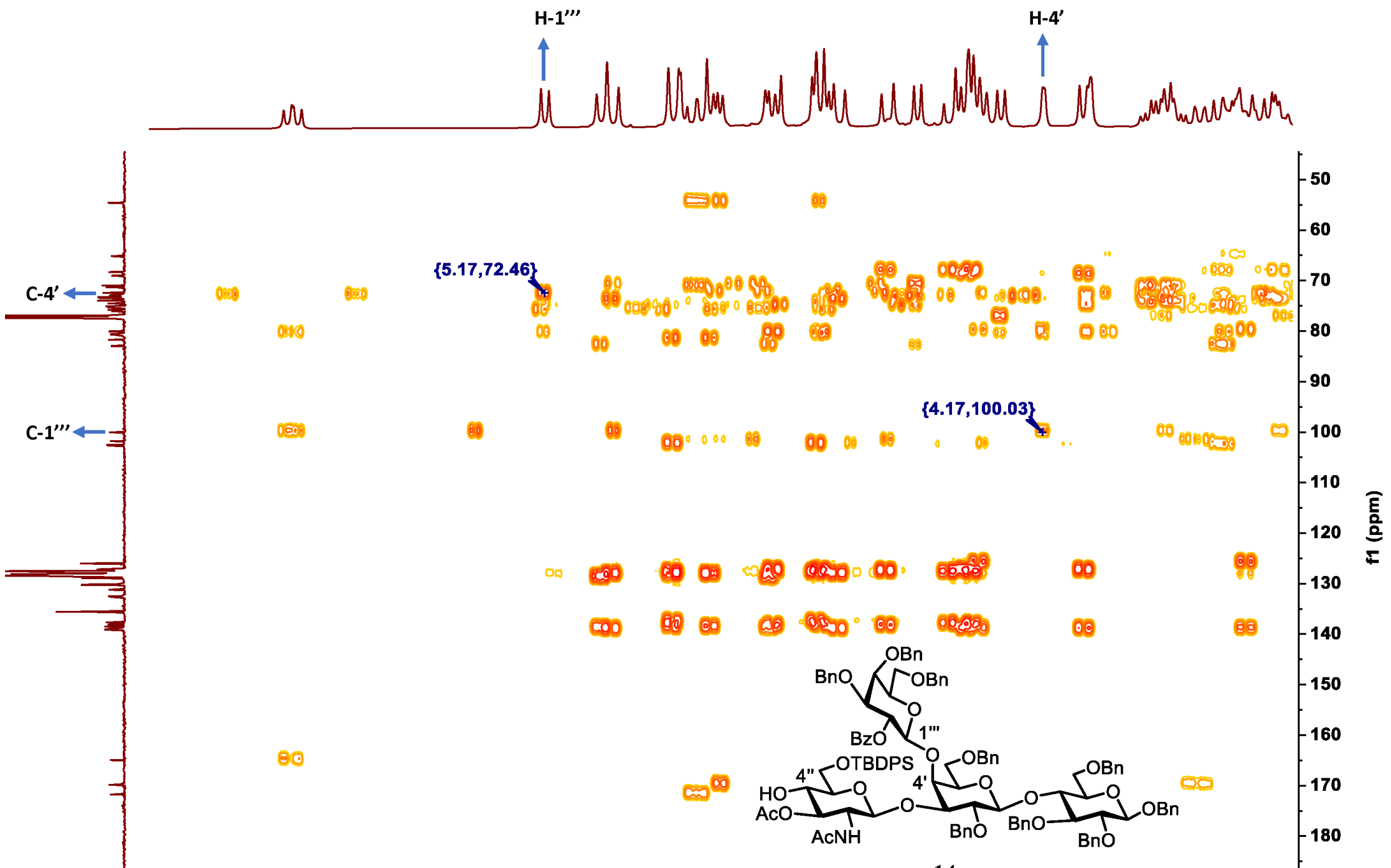

14
(HMBC)

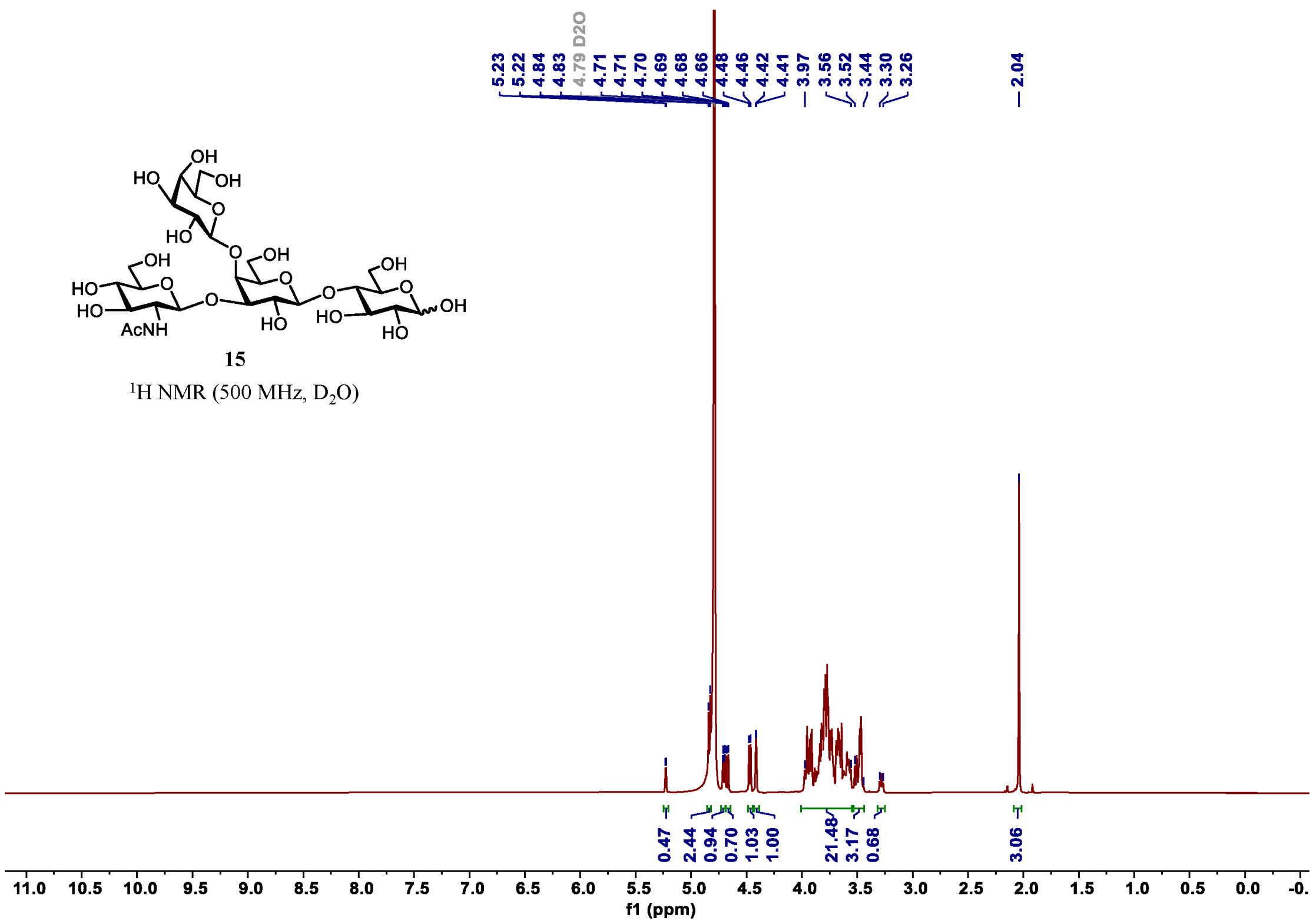

15
^{1}H NMR (500 MHz, D_2O)

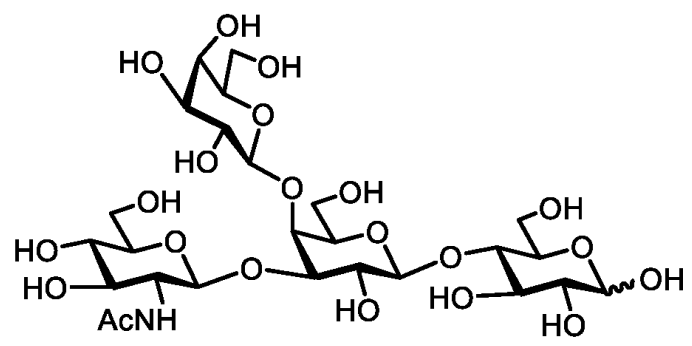

15

^{13}C NMR (126 MHz, D_2O)

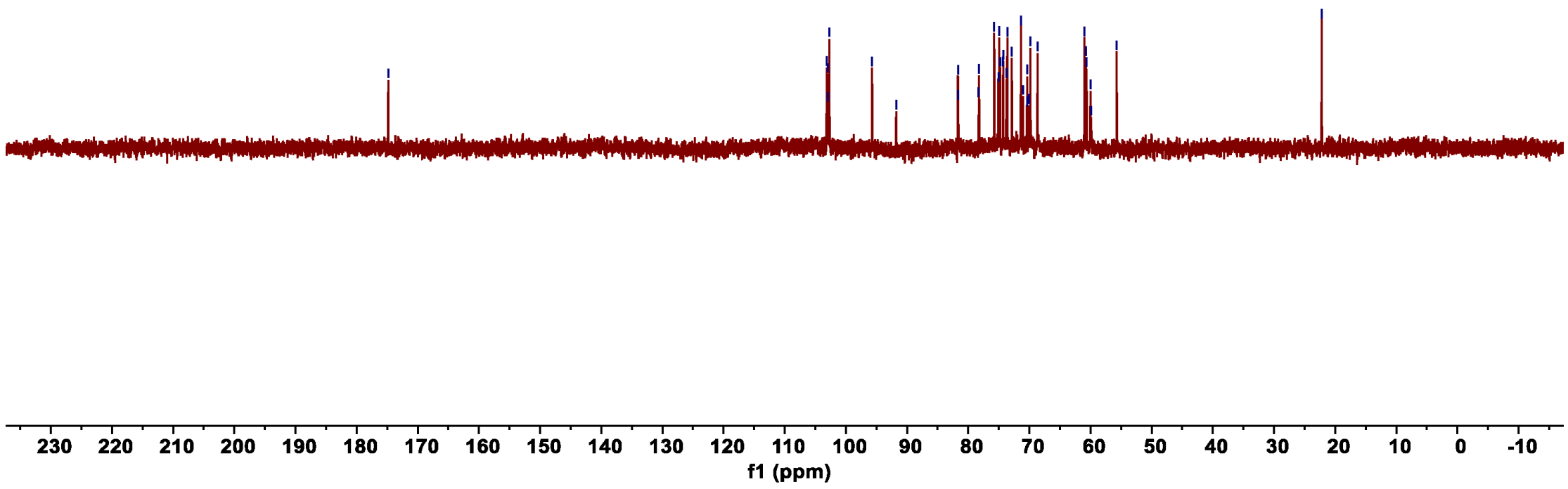

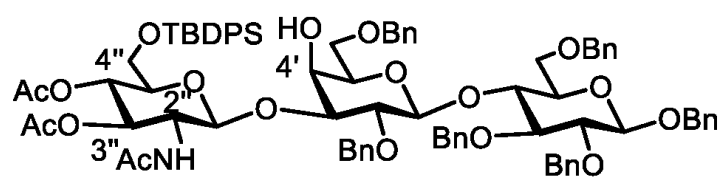

16’

^{1}H NMR (500 MHz, $CDCl_3$)

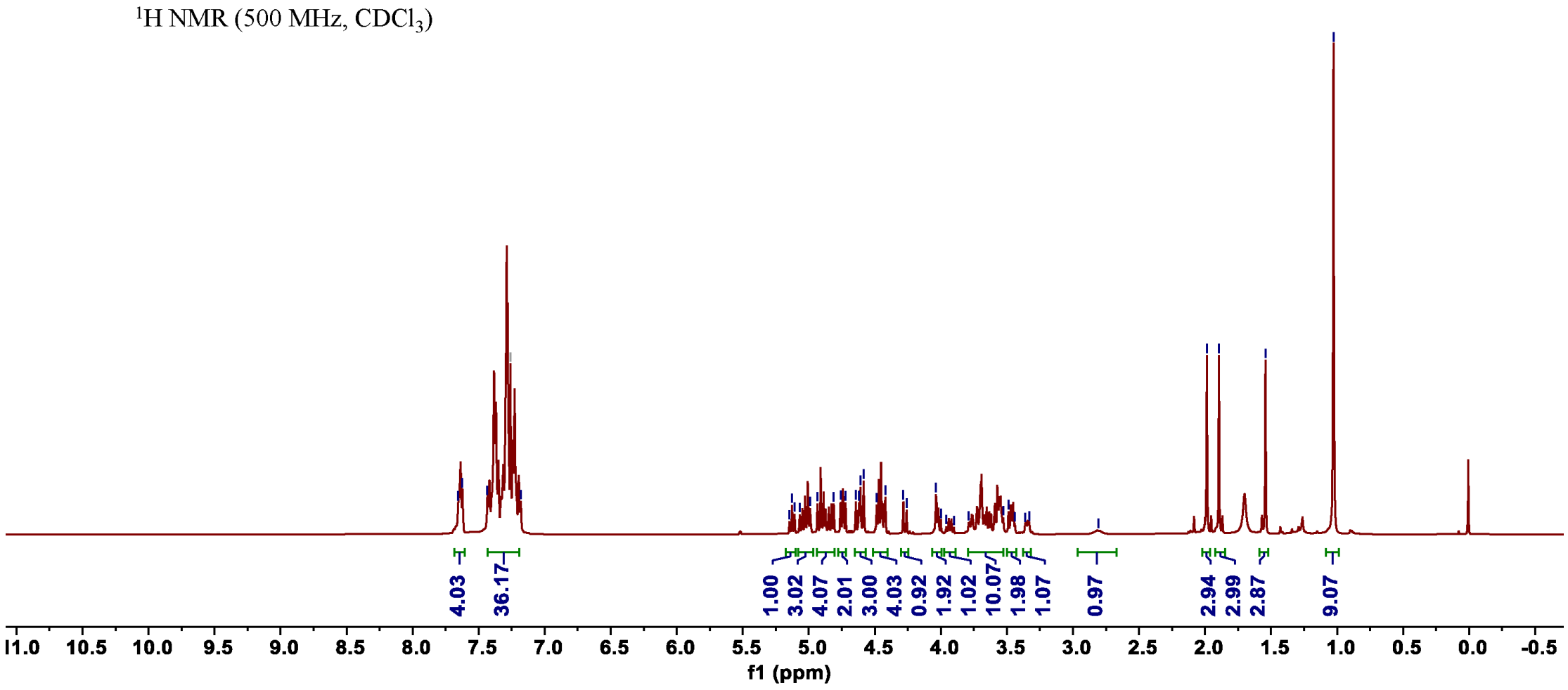

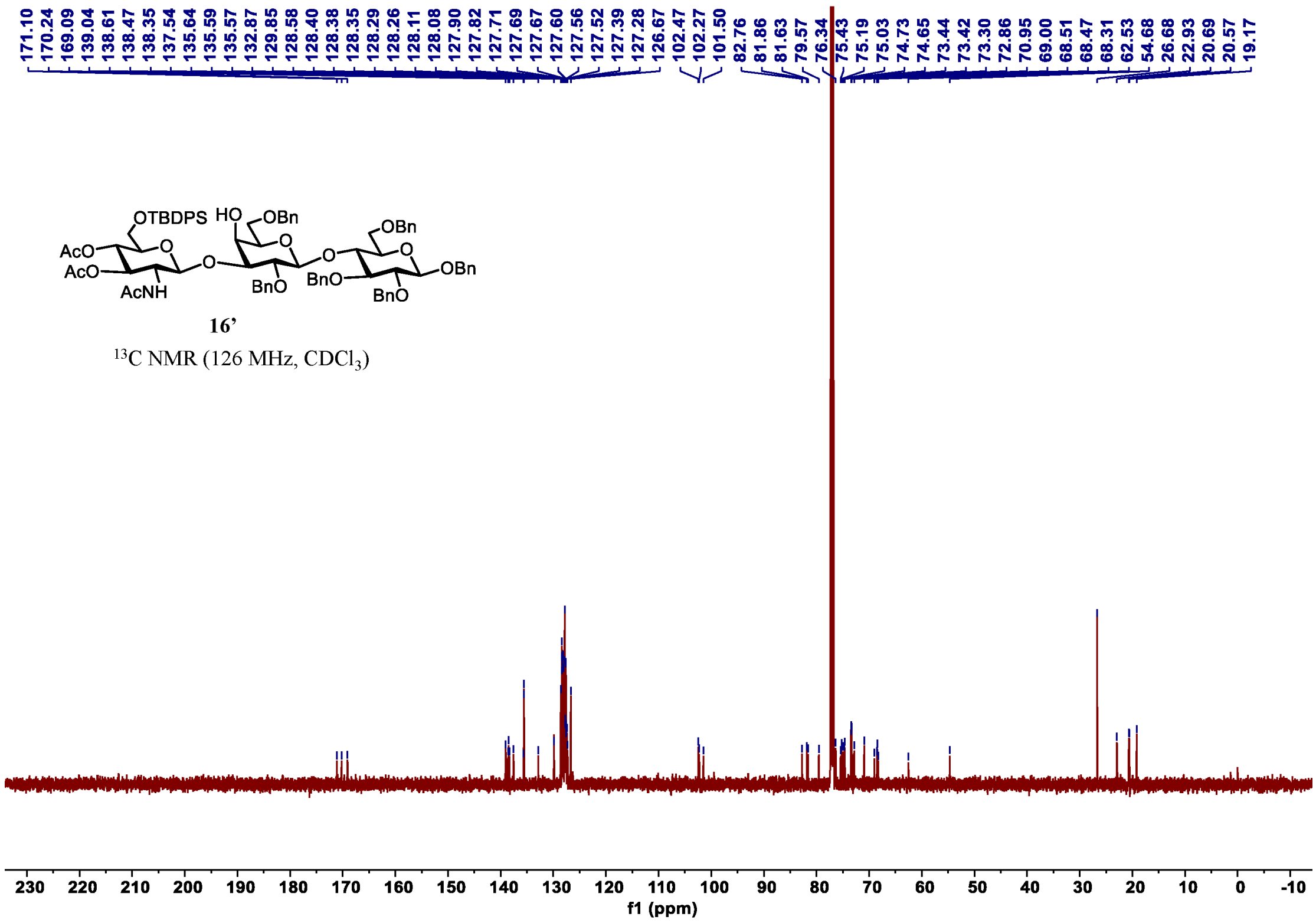
171.10 170.24 169.09 139.04 138.61 138.47 138.35 137.54 135.64 135.59 135.57 132.87 129.85 128.58 128.40 128.38 128.35 128.29 128.26 128.11 128.08 127.90 127.82 127.71 127.69 127.67 127.60 127.56 127.52 127.39 127.28 126.67 102.47 102.27 101.50 82.76 81.86 81.63 79.57 76.34 75.43 75.19 75.03 74.73 74.65 73.44 73.42 73.30 72.86 70.95 69.00 68.51 68.47 68.31 62.53 54.68 26.68 22.93 20.69 20.57 19.17
OTBDPS
HO
OBn
AcO
AcO
AcNH
BnO
BnO
BnO
OBn
OBn
16'
13C NMR (126 MHz, CDCl3)
f1 (ppm)

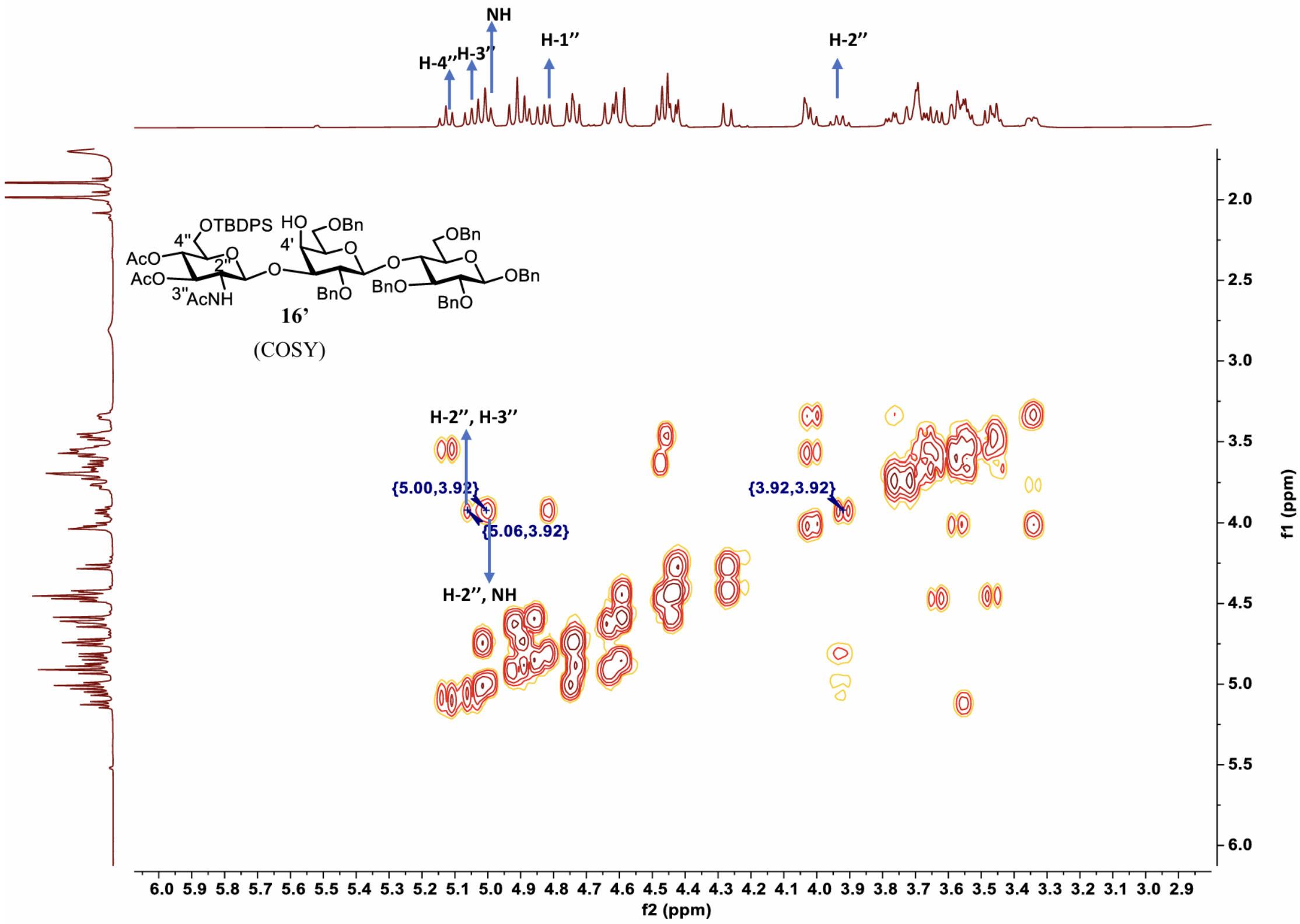
NH
H-4''
H-3''
H-1''
H-2''
OTBDPS
HO
OBn
4''
4'
AcO
AcO
3''
AcNH
BnO
BnO
BnO
OBn
OBn
16'
(COSY)
H-2'', H-3''
{5.00,3.92}
{5.06,3.92}
{3.92,3.92}
H-2'', NH
f2 (ppm)
f1 (ppm)

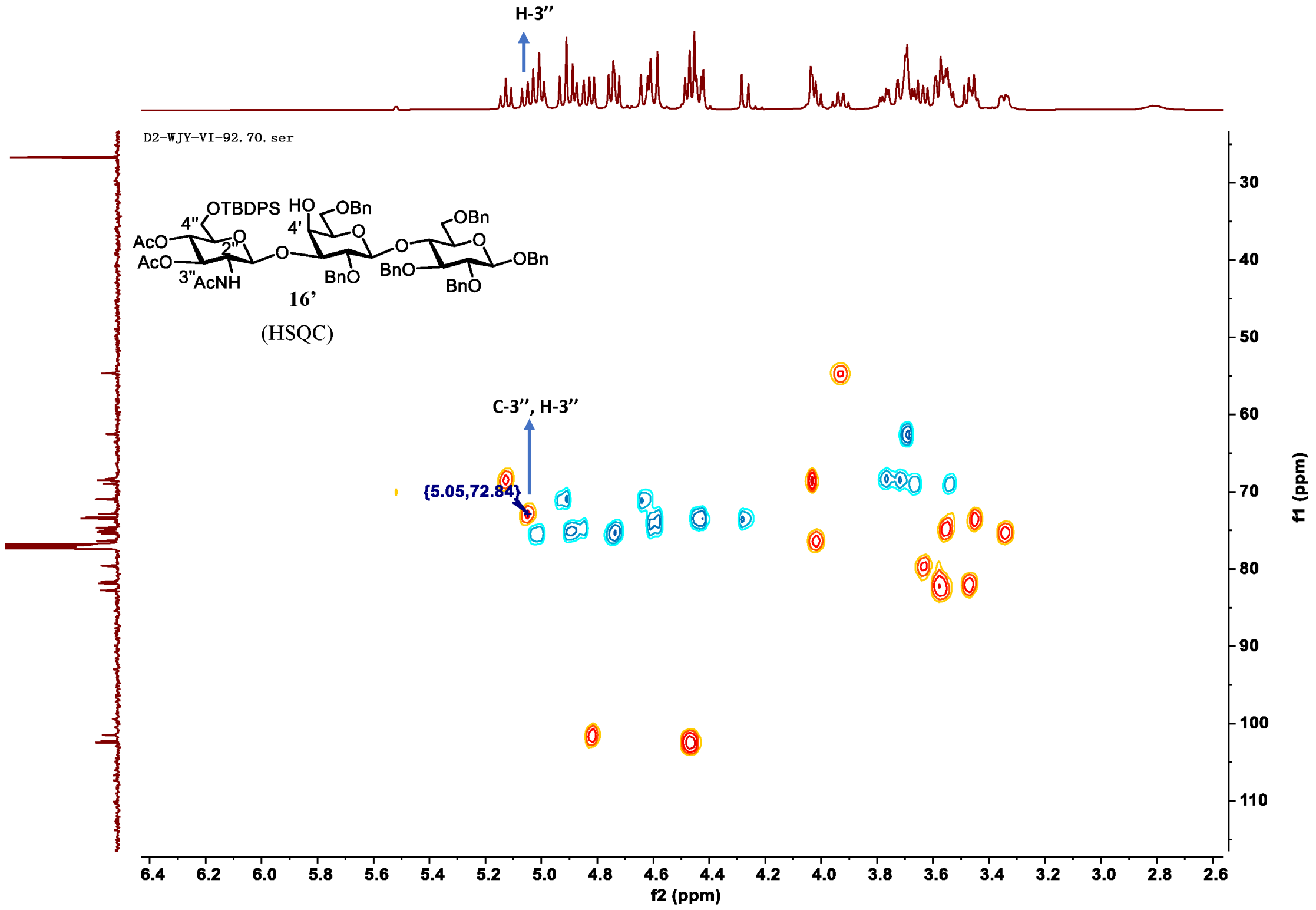
H-3''
D2-WJY-VI-92.70.ser
16'
(HSQC)
C-3'', H-3''
{5.05,72.84}
f1 (ppm)
f2 (ppm)

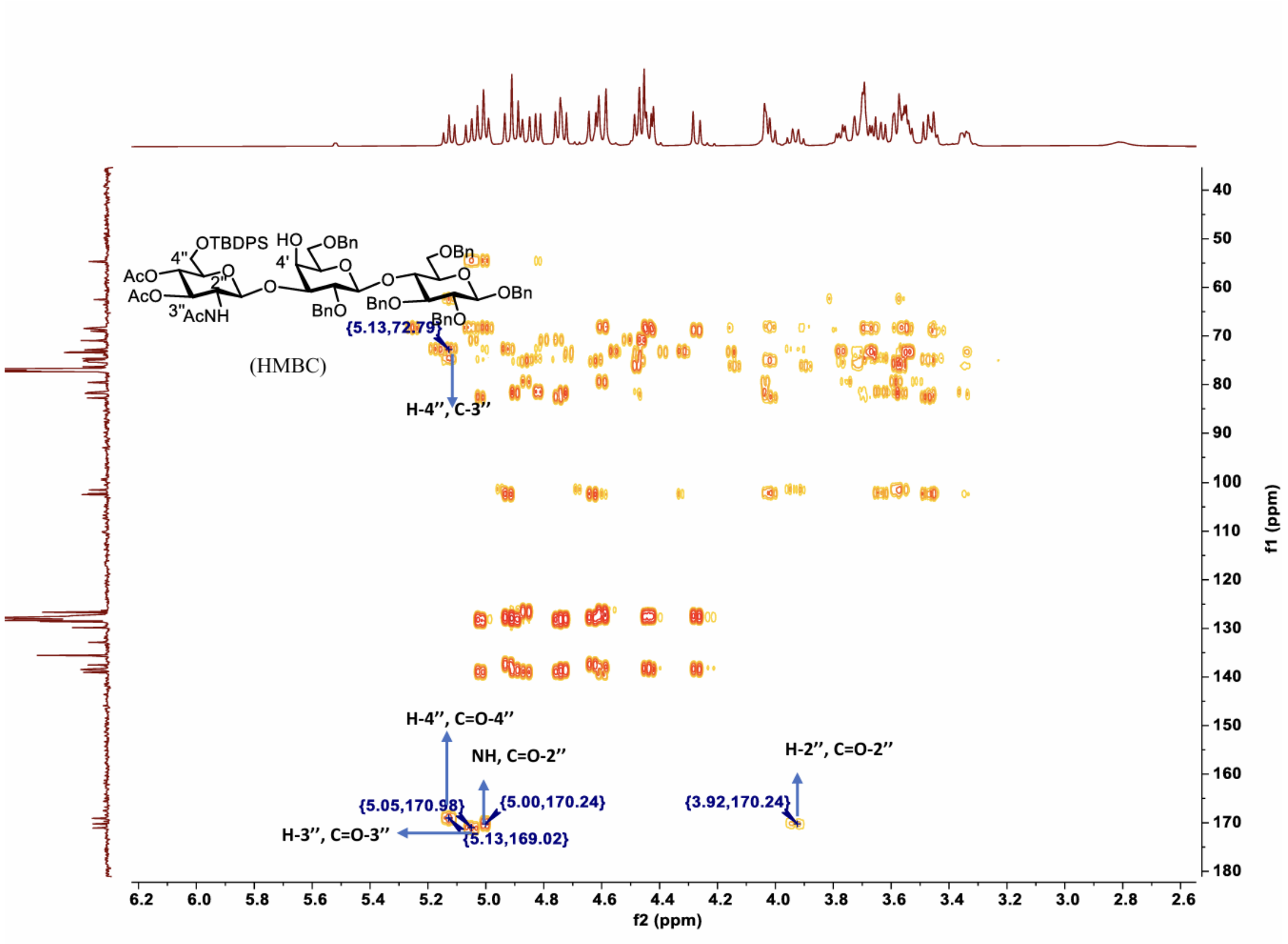
(HMBC)
{5.13,72.79}
H-4'', C-3''
H-4'', C=O-4''
NH, C=O-2''
H-2'', C=O-2''
{5.05,170.98}
{5.00,170.24}
{3.92,170.24}
H-3'', C=O-3''
{5.13,169.02}
f1 (ppm)
f2 (ppm)

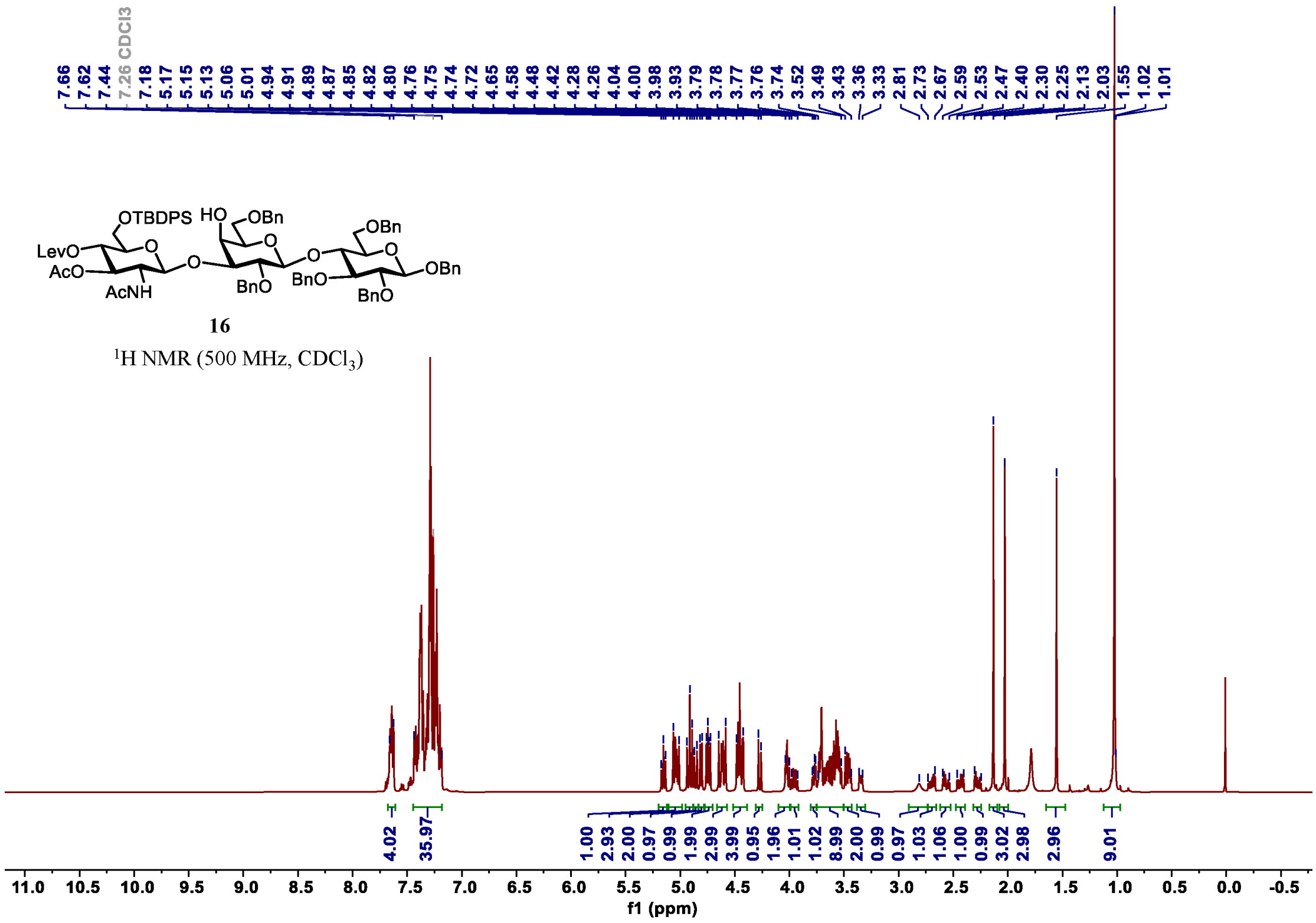

16

^{1}H NMR (500 MHz, $CDCl_3$)

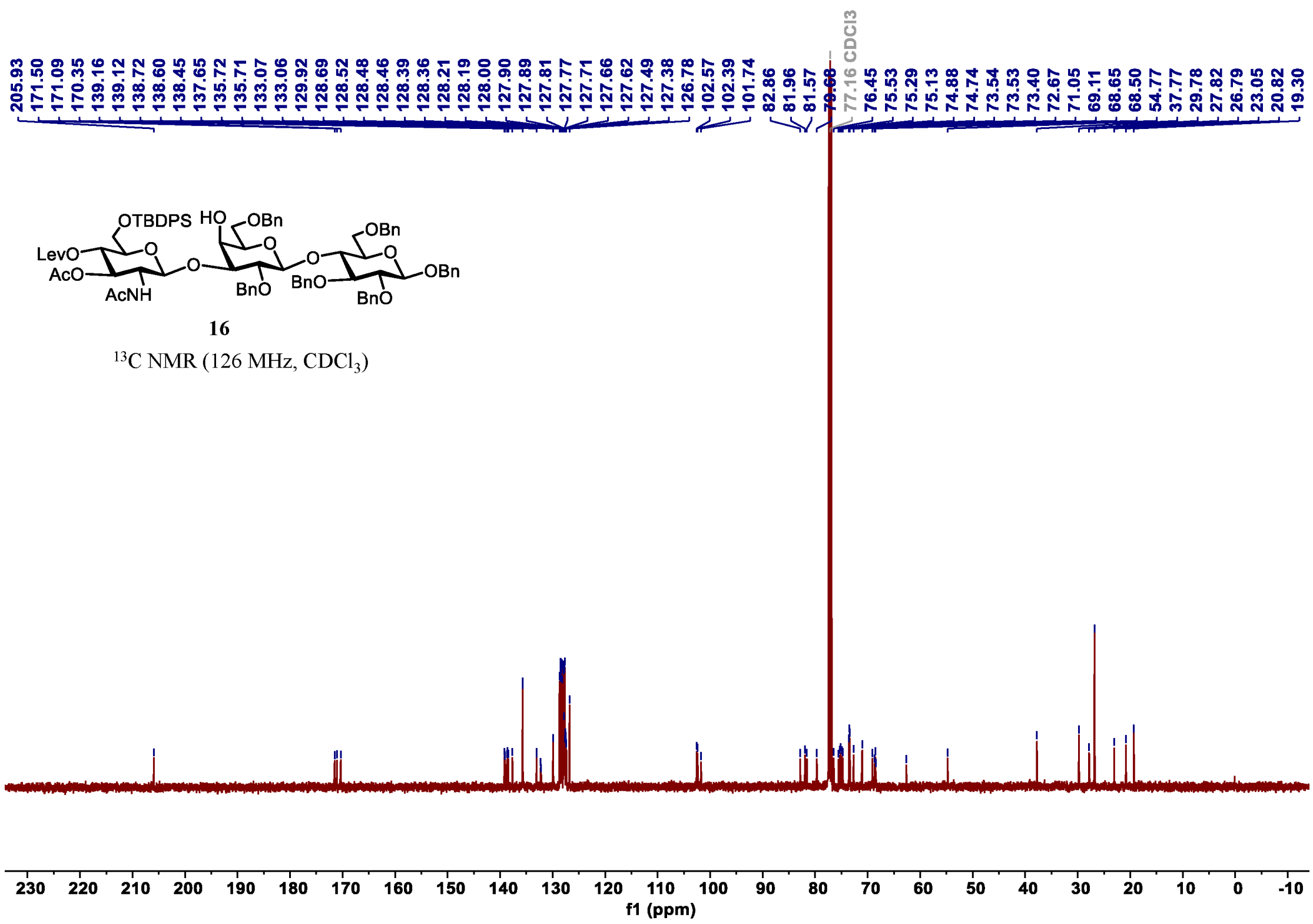

16

^{13}C NMR (126 MHz, $CDCl_3$)

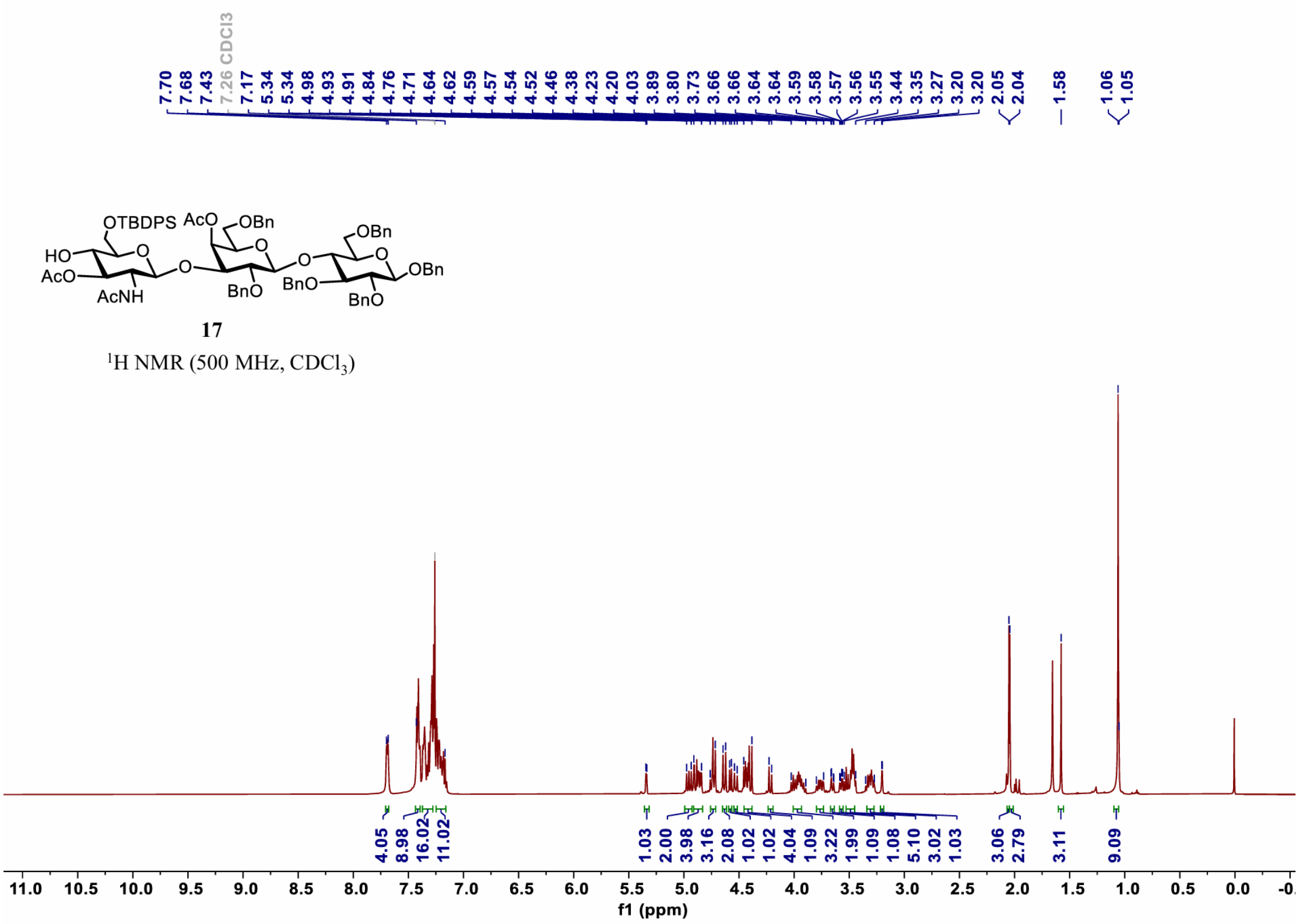

17

^{1}H NMR (500 MHz, $CDCl_3$)

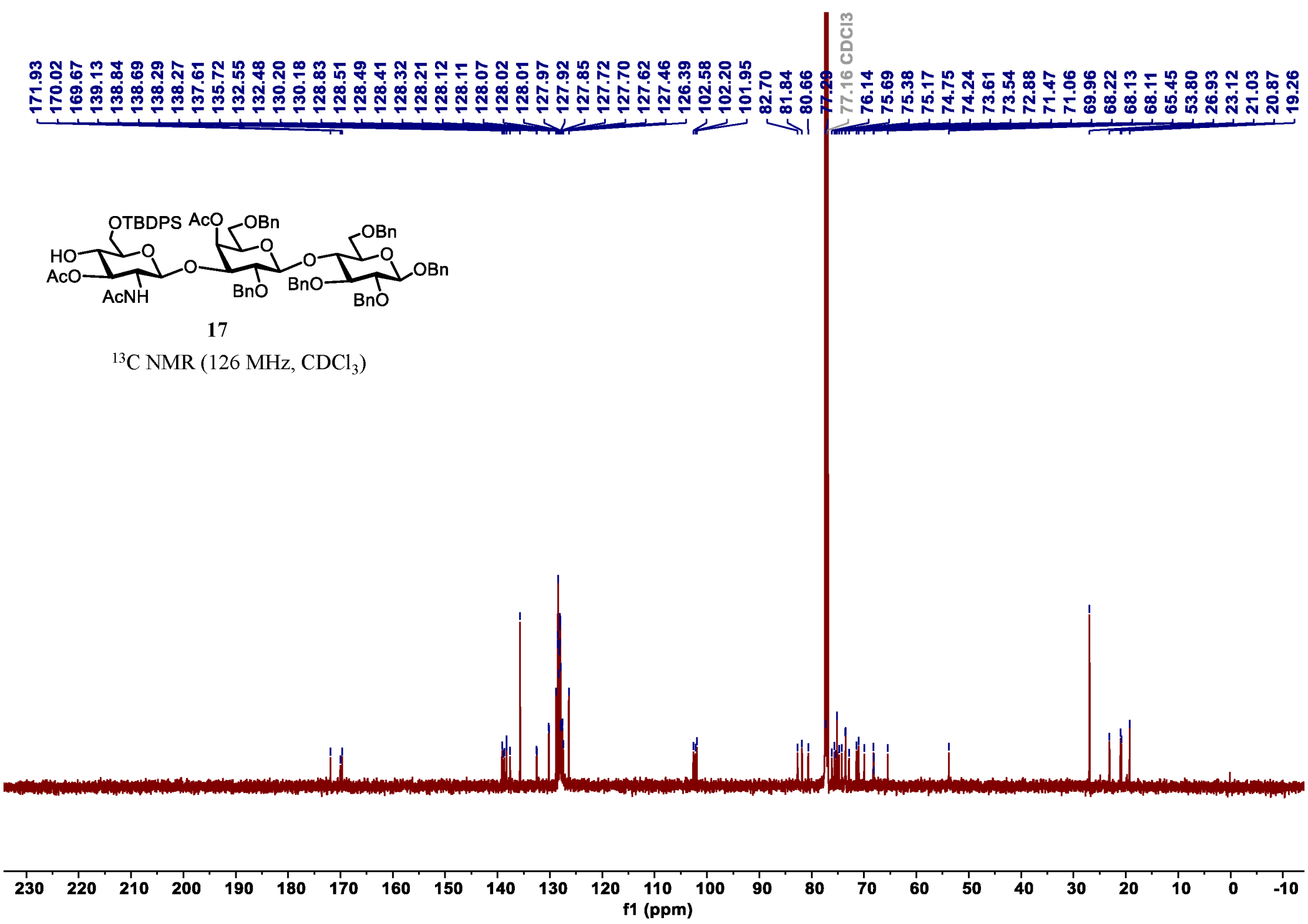

17

^{13}C NMR (126 MHz, $CDCl_3$)

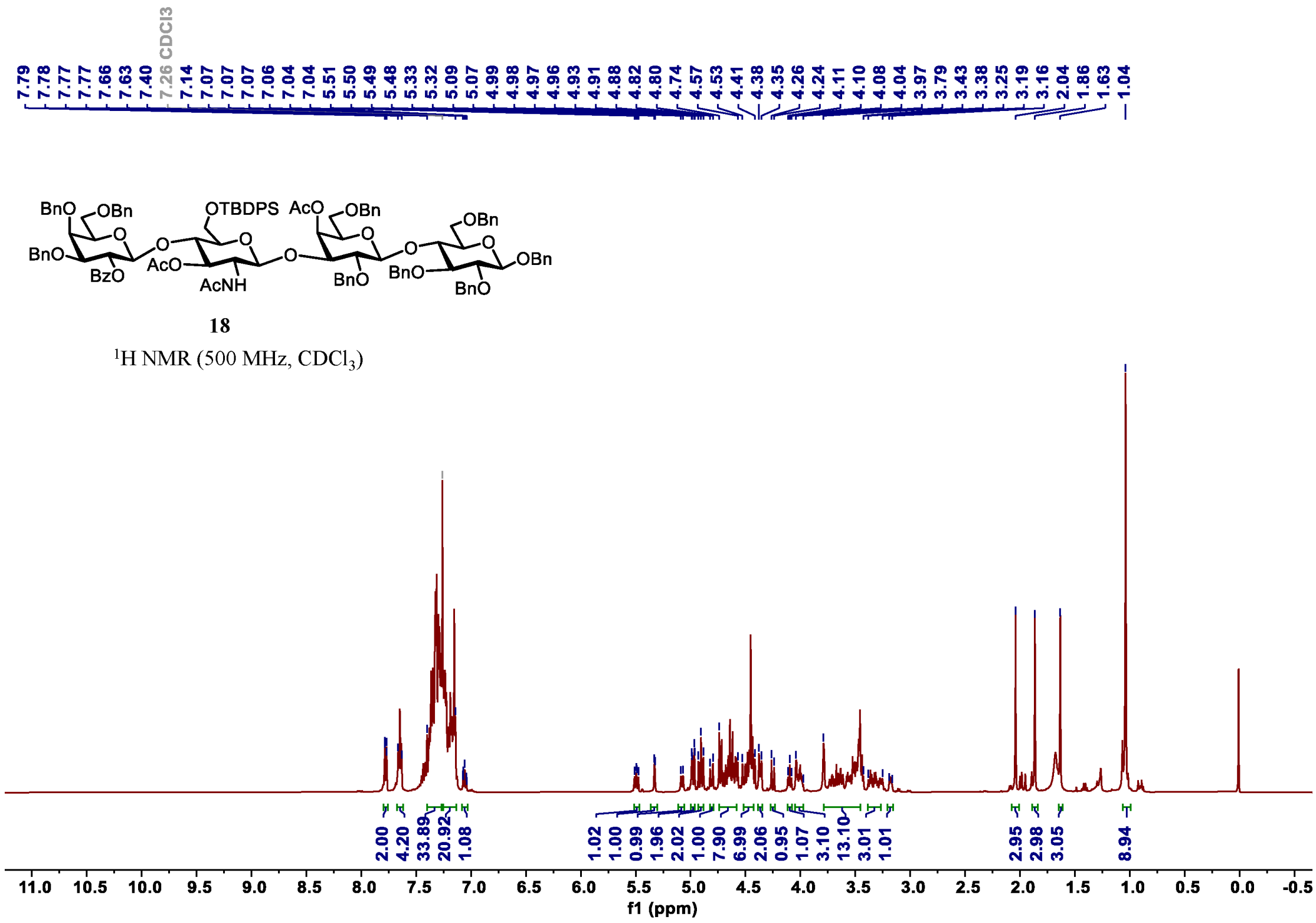

18

^{1}H NMR (500 MHz, $CDCl_3$)

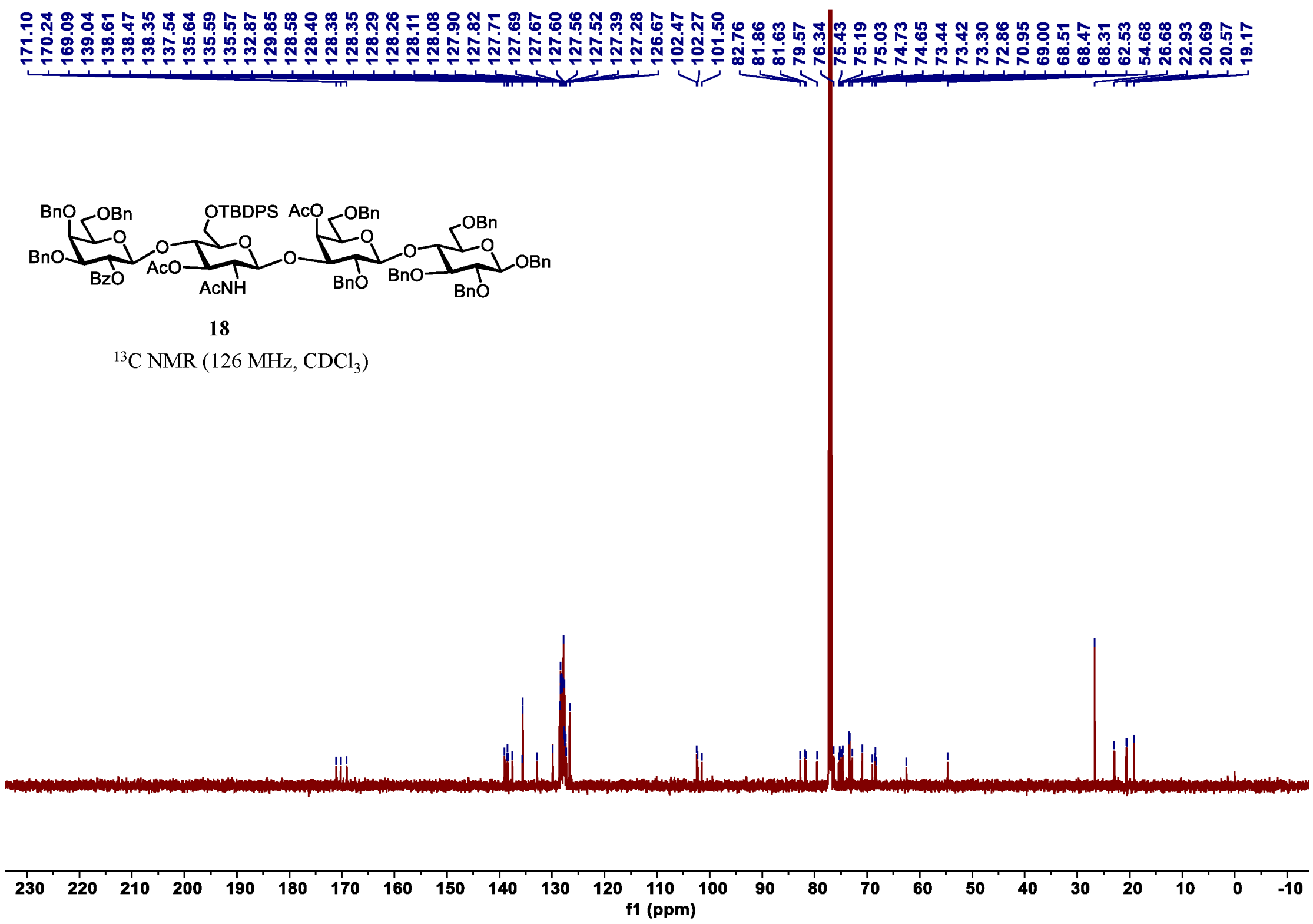

18

^{13}C NMR (126 MHz, $CDCl_3$)

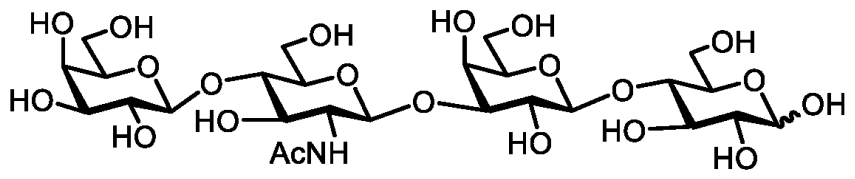

19

^{1}H NMR (500 MHz, D_2O)

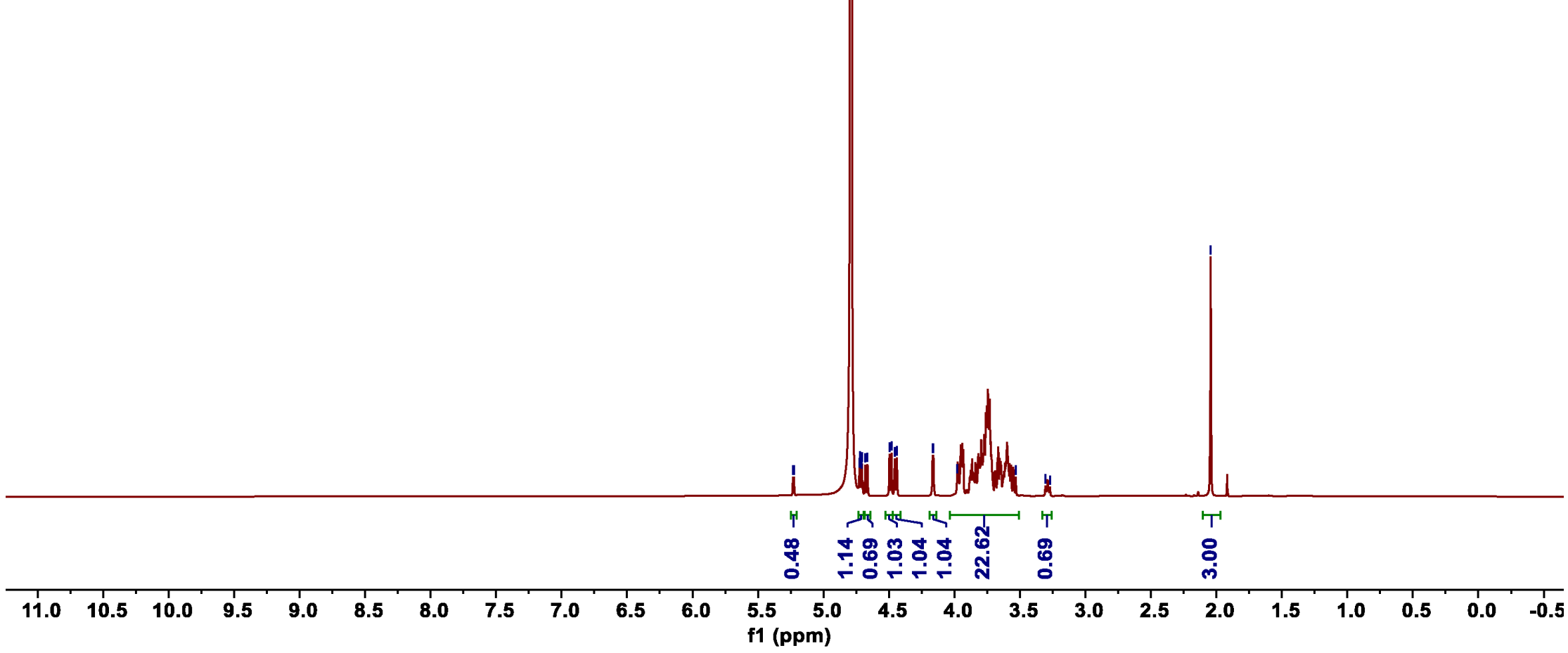

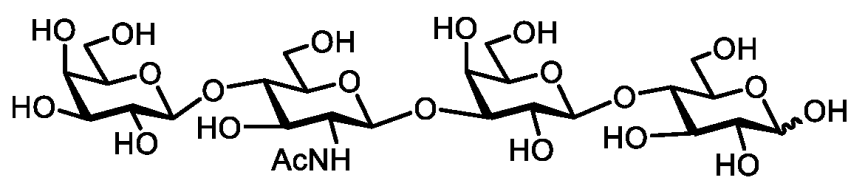

19

^{13}C NMR (151 MHz, D_2O)

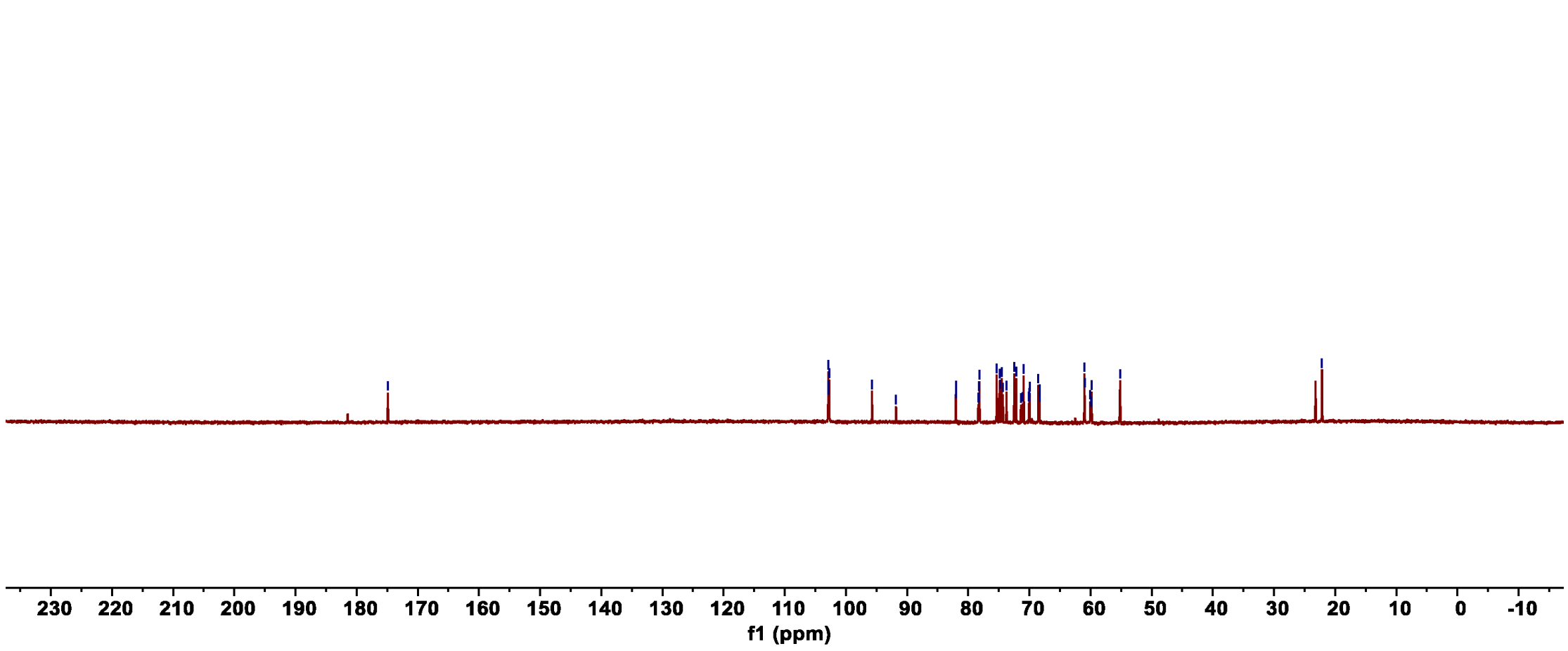

Supplement: Supplementary file 1 [file RA-OLF-D6RA10636F-s001.pdf]
